# Synthesis, ^123^I-Radiolabeling Optimization, and Initial Preclinical Evaluation of Novel Urea-Based PSMA Inhibitors with a Tributylstannyl Prosthetic Group in Their Structures

**DOI:** 10.3390/ijms241512206

**Published:** 2023-07-30

**Authors:** Lutfi A. Hasnowo, Maria S. Larkina, Evgenii Plotnikov, Vitalina Bodenko, Feruza Yuldasheva, Elena Stasyuk, Stanislav A. Petrov, Nikolai Y. Zyk, Aleksei E. Machulkin, Nikolai I. Vorozhtsov, Elena K. Beloglazkina, Valentine G. Nenajdenko, Vladimir Tolmachev, Anna Orlova, Alexander G. Majouga, Mekhman S. Yusubov

**Affiliations:** 1 School of Nuclear Science and Engineering, Tomsk Polytechnic University, Tomsk 634050, Russia or lutf006@brin.go.id (L.A.H.); stasyuk@tpu.ru (E.S.); 2 Polytechnic Institute of Nuclear Technology, National Research and Innovation Agency, Yogyakarta 55281, Indonesia; 3 Research Centrum for Oncotheranostics, Research School of Chemistry and Applied Biomedical Sciences, Tomsk Polytechnic University, Tomsk 634050, Russia; marialarkina@mail.ru (M.S.L.); plotnikov.e@mail.ru (E.P.); bodenkovitalina@gmail.com (V.B.); fsy1@tpu.ru (F.Y.); yusubov@tpu.ru (M.S.Y.); 4 Department of Pharmaceutical Analysis, Siberian State Medical University, Tomsk 634050, Russia; 5 Mental Health Reseach Institute, Tomsk National Research Medical Center, Russian Academy of Sciences, Tomsk 634014, Russia; 6 Department of Chemistry, M.V. Lomonosov Moscow State University Leninskie Gory, 1–3, Moscow 119991, Russia; stanislavpetrovsh1994@gmail.com (S.A.P.); zyknikola@gmail.com (N.Y.Z.); alekseymachulkin@rambler.ru (A.E.M.); nvor@rambler.ru (N.I.V.); nenajdenko@gmail.com (V.G.N.); 7 Department of Immunology, Genetics and Pathology, Uppsala University, 75185 Uppsala, Sweden; vladimir.tolmachev@igp.uu.se; 8 Department of Medicinal Chemistry, Uppsala University, 75183 Uppsala, Sweden; anna.orlova@ilk.uu.se; 9 Faculty of Chemistry, Dmitry Mendeleev University of Chemical Technology of Russia, Miusskaya sq. 9, Moscow 125047, Russia; alexander.majouga@gmail.com

**Keywords:** DCL ligand, iodine radioisotopes, radiolabeled pharmaceuticals, targeted delivery, prostate-specific membrane antigen, prostate cancer

## Abstract

Prostate-specific membrane antigen (PSMA) has been identified as a target for the development of theranostic agents. In our current work, we describe the design and synthesis of novel N-[N-[(S)-1,3-dicarboxypropyl]carbamoyl]-(S)-L-lysine (DCL) urea-based PSMA inhibitors with a chlorine-substituted aromatic fragment at the lysine ε-nitrogen atom, a dipeptide including two phenylalanine residues in the L-configuration as the peptide fragment of the linker, and 3- or 4-(tributylstannyl)benzoic acid as a prosthetic group in their structures for radiolabeling. The standard compounds [^127^I]PSMA-m-IB and [^127^I]PSMA-p-IB for comparative and characterization studies were first synthesized using two alternative synthetic approaches. An important advantage of the alternative synthetic approach, in which the prosthetic group (NHS-activated esters of compounds) is first conjugated with the polypeptide sequence followed by replacement of the Sn(Bu)_3_ group with radioiodine, is that the radionuclide is introduced in the final step of synthesis, thereby minimizing operating time with iodine-123 during the radiolabeling process. The obtained DCL urea-based PSMA inhibitors were radiolabeled with iodine-123. The radiolabeling optimization results showed that the radiochemical yield of [^123^I]PSMA-p-IB was higher than that of [^123^I]PSMA-m-IB, which were 74.9 ± 1.0% and 49.4 ± 1.2%, respectively. The radiochemical purity of [^123^I]PSMA-p-IB after purification was greater than 99.50%. The initial preclinical evaluation of [^123^I]PSMA-p-IB demonstrated a considerable affinity and specific binding to PC-3 PIP (PSMA-expressing cells) in vitro. The in vivo biodistribution of this new radioligand [^123^I]PSMA-p-IB showed less accumulation than [^177^Lu]Lu-PSMA-617 in several normal organs (liver, kidney, and bone). These results warrant further preclinical development, including toxicology evaluation and experiments in tumor-bearing mice.

## 1. Introduction

Prostate cancer is currently one of the most commonly reported oncological diseases in the male population [1]. Although the development and practical use of radiopharmaceuticals mediated by prostate-specific membrane antigen (PSMA) for the diagnosis and endoradiotherapy of prostate cancer have recently shown good results, further development continues in a search of new and more efficient targeting agents capable of enhancing the effect of treatment.

PSMA is also known as the human neuropeptidase glutamate-preferring carboxypeptidase II (GCP II). PSMA has been identified as a target for the development of theranostic agents. PSMA is overexpressed in prostate cancer cells compared with healthy prostate cells. PSMA expression also exists in numerous tissue types, including the testis, ovary, brain, salivary gland, small intestine, colon, liver, spleen, breast, kidney tissue, and normal prostate tissues have the highest expression levels [1,2]. The level of PSMA expression corresponds with tumor aggressiveness. PSMA is a target for delivery, prostate cancer diagnostics, and intraoperative guidance due to its high expression in prostate cancer [3,4].

Small molecule ligands are one of three classes of PSMA-targeting molecules. Small molecules have a number of advantages over antibodies and aptamers, including ease of synthesis and modification, absence of immunogenicity, improved pharmacokinetic properties, and rapid clearance from normal tissues [5,6,7,8,9]. In addition to optimizing the structure of the ligand itself, in recent years, there has been an increase in the number of publications devoted to optimizing the structure of the linker incorporating the vector fragment with a diagnostic agent [10]. As a result, a variety of highly promising therapeutic and diagnostic conjugates have been developed [11,12,13,14,15,16].

Urea-based ligands are the most widely developed at present [10,17,18]. The advantages of urea-based ligands include the significant potential for further modification, adequate bioavailability in comparison with ligands based on phosphinic and phosphonic acids [19], and this type of ligand is more stable than thiol-based ligands [20,21]. N-[N-[(S)-1,3-dicarboxypropyl]carbamoyl]-(S)-L-lysine (DCL) is one of the most developed urea-based ligands due to its promising potential [17,18]. A number of publications confirmed that modification of the DCL ligand structure affects its affinity for PSMA. We assume that the modification of the DCL linker with a dipeptide fragment containing two phenylalanine residues improves the affinity properties of the PSMA ligand by taking advantage of the hydrophobic interaction with the S1 hydrophobic pocket of PSMA [22,23].

^123^I, as a gamma-ray emitter with an energy of 159 keV, is an ideal radionuclide for use in single-photon-emission computerized tomography (SPECT) diagnostics. The gamma emission of ^123^I enables excellent imaging with low background activity (80% efficiency for a 1-inch-thick crystal). It delivers substantially lower absorbed doses to the patients while maintaining comparable activity to ^131^I [24].

The commonly used method for the radioiodination of peptides is direct labeling. Radioiodine is oxidized in situ using an oxidizing agent to form I^+^ ions, which then attack the activated phenolic ring of the amino acid tyrosine in proteins and produce a stable covalent bond. This is not a problem when using prosthetic groups in the labeling of targeting protein or peptide. Radioiodination of a conjugate, in which the peptide is conjugated with a prosthetic group before iodination, is considered an appropriate idea [25].

**Figure 1:**
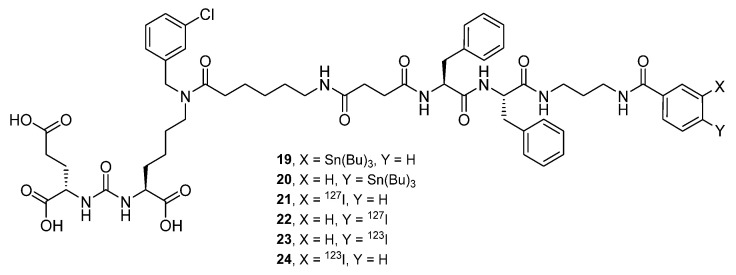
The general structure of the obtained ligands.

Thus, this work aimed to synthesize novel DCL urea-based ligands with a chlorine-substituted aromatic fragment at the lysine ε-nitrogen atom, a dipeptide including two phenylalanine residues in the L-configuration as the peptide fragment of the linker, and 4- or 3-(tributylstannyl)benzoic acid as prosthetic group in its structures. The general structure of the obtained ligands is shown in Figure 1. These ligands were studied as novel PSMA ligands by conducting radiolabeling optimization with iodine-123. The [^123^I]PSMA-p-IB ligand was tested in the initial preclinical evaluation. As a comparison with the biodistribution of [^123^I]PSMA-p-IB in normal mice, we used [^177^Lu]Lu-PSMA-617, which is known to have demonstrated promising results in clinical studies.

## 2. Results

### 2.1. Synthesis

**Scheme 1:**
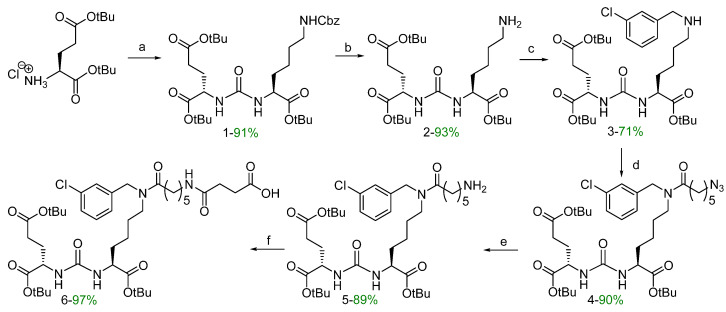
Synthesis of PSMA-vector fragment **6**. Reagents and conditions: (**a**) (1) triphosgene, DCM, −78 °C; (2) H-Lyz(Cbz)-O-tBu·HCl, Et_3_N, 20 °C; (**b**) H_2_, Pd/C (10%), MeOH; (**c**) (1) 3-Cl-C_6_H_4_-CHO, DCM; (2) NaBH_4_; (**d**) PyBOP, DIPEA, DMF, N_3_(CH_2_)_5_COOH; (**e**) THF/H_2_O, Ph_3_P, 50 °C; (**f**) (1) Succinic anhydride, DCM, DIPEA; (2) MeOH; (3) HCl (0.1 M). All amino acids have the L-configuration.

**Scheme 2:**
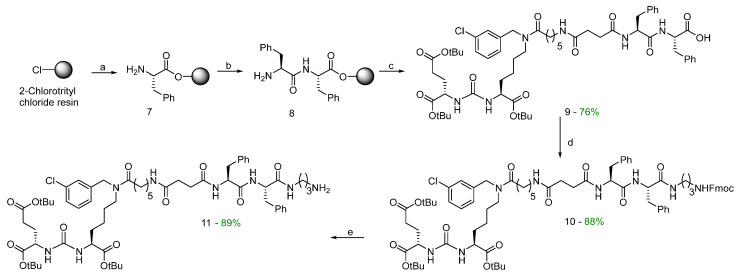
Synthesis of the polypeptide sequence **11**. (**a**) (1) FmocPhe-OH(L), DIPEA, DMF; (2) 4-methylpiperidine/DMF; (**b**) (1) FmocPhe-OH(L), HBTU, HOBt, DIPEA; (2) 4-methylpiperidine/DMF. (**c**) (1) compound **6**, HBTU, HOBt, DIPEA, DMF; (2) DCM/TFA (99.25%/0.75%; *v*/*v*); (**d**) FmocNH(CH_2_)_3_NH_3_^+^TFA^−^, HBTU, HOBt, DIPEA, DMF; (**e**) Et_2_NH/DMF.

**Scheme 3:**
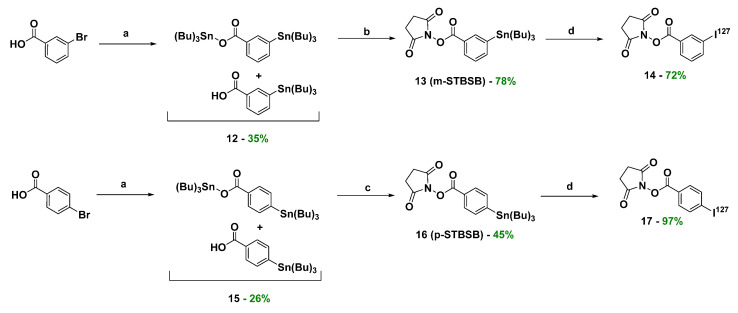
Synthesis of prosthetic groups (compounds **13**, **14**, **16,** and **17**). (**a**) (1) BuLi, THF, −78 °C, Ar; (2) (Bu)_3_SnCl, THF, −78 °C, Ar; (**b**) NHS, EDC_*_HCl, DMAP, DCM, Ar, r.t.; (**c**) NHS, DCC, THF, Ar, r.t.; (**d**) (1) I_2_ in 0.1 NaOH V_1_; (2) AcOH (3%) in CHCl_3_ V_2_; V_1_ = V_2_; (3) TBHP/CHCl_3_ (10% *w*/*v*).

**Scheme 4:**
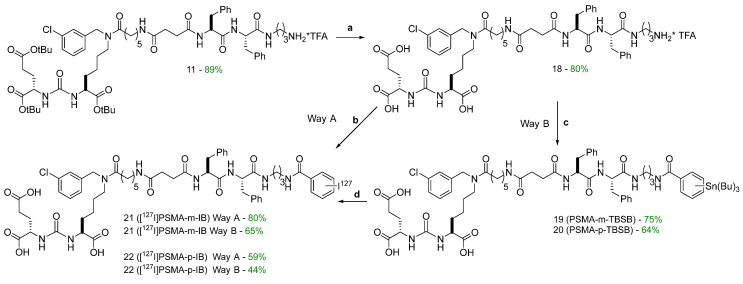
Synthesis of PSMA-m-TBSB (**19**), PSMA-p-TBSB (**20**); [^127^I]PSMA-m-IB (**21**), and [^127^I]PSMA-p-IB (**22**). (**a**) DCM/TFA/TIPS/H_2_O (46.25%/46.25%/5%/2.5% *v*/*v*); (**b**) 14/17, DIPEA, DMF (**c**) 13/16, DIPEA, DMF (**d**) (1) I_2_ in 0.1 NaOH V_1_, (2) AcOH (3%) in CHCl_3_ V_2_; V_1_ = V_2_, (3) TBHP/CHCl_3_ (10% *w*/*v*).

For the synthesis of target compounds **19**–**22** (PSMA-m-TBSB, PSMA-p-TBSB, [^127^I]PSMA-m-IB, and [^127^I]PSMA-p-IB), three schemes (Scheme 1, Scheme 2 and Scheme 3) including the sequential solution of the following five synthetic objectives were chosen:

1. The synthesis of PSMA vector **6**, which is a derivative of DCL urea modified with ε-aminocaproic acid (Ahx) and succinic acid (Suc) (Scheme 1);
2. The formation of a peptide linker (compound **8**) using solid-phase peptide synthesis (SPPS) (Scheme 2) and further connection of the vector fragment (compound **6**) with a dipeptide linker (Scheme 2);
3. Modification of the C-terminal fragment of the polypeptide sequence (compound **11**) to connect it with the prosthetic groups of N-succinimidyl 3-(tributylstannyl)benzoate (m-STBSB)/compound **13** and N-succinimidyl 4-(tributylstannyl)benzoate m-STBSB/compound **16** (Scheme 2);
4. Obtaining NHS-activated esters of m-STBSB (compound **13**) and m-STBSB (compound **16**) prosthetic groups (Scheme 3);
5. Combining a PSMA ligand with m-STBSB or p-STBSB prosthetic groups, as well as the synthesis of [^127^I]PSMA-m-IB (compound **21**) or [^127^I]PSMA-p-IB (compound **22**) conjugates in two alternative ways (Scheme 4).

**Figure 2:**
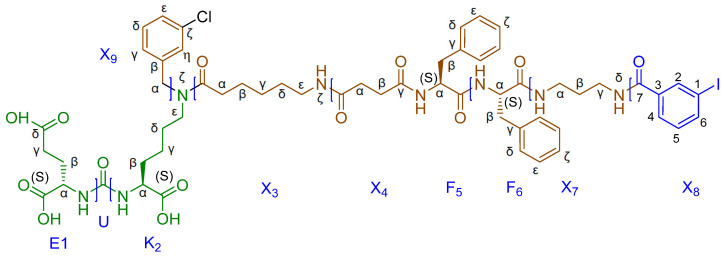
Denotations of structural fragments of synthesized compounds (using the example of [^127^I]PSMA-m-IB (**21**)).

Figure 2 demonstrates the denotations of structural fragments of the synthesized compounds used in the text (using the example of compound **21**, [^127^I]PSMA-m-IB). This denotation of fragment structures is employed in the characterization of the resulting compounds, which is presented in the Materials and Methods section.

The initial stages of the vector fragment **6** synthesis (Scheme 1) were realized by previously described methods [26]. Compound **6** was prepared by coupling succinic anhydrides with the tert-butylated compound **5** (Scheme 1); the resulting products contained a free carboxylic group that was suitable for the further addition of the peptide fragment.

The second stage of the synthesis involved assembling the peptide sequence Phe(L)-Phe(L) to obtain a highly specific PSMA vector (compound **11**), using SPPS on a cross-linked styrene-divinylbenzene (1%) copolymer matrix (2-CTC resin) (Scheme 2). The selected reaction sequence was a classical scheme of peptide synthesis:

1. Immobilization of an N-substituted amino acid onto a solid-phase substrate;
2. Removal of the protective group;
3. Modification of the NH_2_- group of the amino acid (stages 2 and 3 are repeated the required number of times to assemble the necessary peptide sequence);
4. Removal of the modified amino acid sequence from the 2-CTC resin [27].

The use of the 2-CTC resin allows acid-labile functional groups to be kept intact, since the removal of the amino acid sequence from the resin proceeds under mild conditions (in this case, DCM/TFA—99.25%/0.75% *v*/*v*; the reaction does not affect the COOBu^t^ acid-labile groups) [28].

Then, the vector fragment was attached to dipeptide **8** immobilized on a 2-CTC resin, using HOBt/HBTU/DIPEA as activating agents. After that, the modified peptide was removed from the polymer matrix by DCM/TFA treatment (99.25–0.75%, *v*/*v*). As a result, compound **9** was isolated as an individual stereoisomer, which was confirmed by ^1^H and ^13^C NMR spectral data, LCMS, and HRMS.

At the third stage, a fragment of NH_2_(CH_2_)_3_NHFmoc was supposed to be introduced into compound **9** by a peptide synthesis reaction, according to an optimized technique [26], which would then be used to obtain compound **11** by Fmoc deprotection. During the synthesis of compound **10**, it was found that the presence of a Phe-Phe-(CH_2_)_3_-Fmoc fragment in the molecule leads to the appearance of strong gelation properties of the target compound, which greatly complicates its isolation and purification [29]. Nevertheless, product **10** was isolated as an individual stereoisomer with an 88% yield. Next, the Fmoc protection was removed to obtain product **11** (Scheme 2).

The fourth stage was the preparation of NHS-activated esters of the prosthetic groups p-STBSB and p-STBSB (Scheme 3). The synthesis was carried out similarly to the method described in the article [30], with some modifications (see Materials and Methods).

At the final stage, the protective *tert*-butyl groups of compound **11** were removed by TFA action (Scheme 4). During the synthesis, the effectiveness of obtaining the target conjugates **21**, **22** was evaluated by comparing two alternative synthetic approaches (Scheme 4, Table 1). The first approach involved the conjugation of the polypeptide sequence **18** with NHS-activated esters of the m-S [^127^I]IB **14**) and p-S [^127^I]IB (**17**) prosthetic groups (Way A). The second approach involved the reaction of the polypeptide sequence **18** with the obtained NHS-activated esters of compounds m-STBSB (**13**) and p-STBSB (**16**), followed by the replacement of the Sn(Bu)_3_ group with ^127^I (Way B). The pros and cons of each of the approaches as well as the total yield relative to compound **18** are shown in Table 1. From the data presented, it can be seen that the total yield relative to the prosthetic group does not differ significantly between the chosen pathways. It should also be emphasized that the important advantage of Way B relative to Way A is that the radionuclide is introduced at the last stage of synthesis, thus minimizing the time of operation with ^123^I.

### 2.2. Radiolabeling Optimization of PSMA-p-TBSB with ^123^I: Physico-Chemical Study

**Figure 3:**
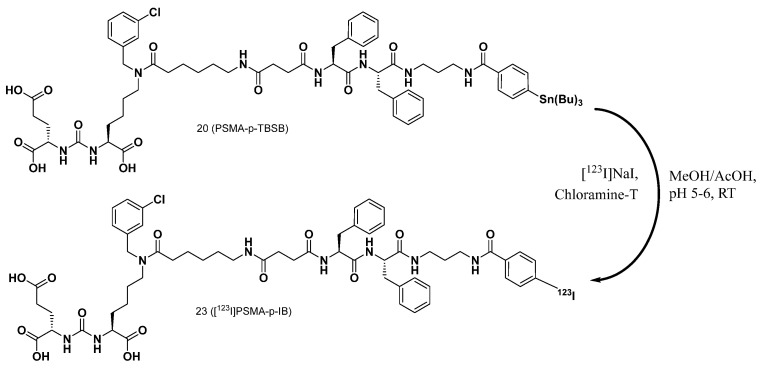
Radiolabeling scheme of the PSMA-p-TBSB (compound **20**) ligand by ^123^I to obtain [^123^I]PSMA-p-IB (compound **23**).

PSMA-p-TBSB (compound **20**) was used as a model for studying the radiolabeling optimization with ^123^I and for performing the initial preclinical evaluation of these novel PSMA-targeting ligands. Radiolabeling of the novel PSMA-targeting ligand by ^123^I was conducted via an electrophilic radioiodination reaction by incubation with [^123^I]NaI in the presence of chloramine-T as an oxidizing agent. This radiolabeling reaction produced [^123^I]PSMA-p-IB (compound **23**). The scheme of the radiolabeling reaction is shown in Figure 3. Radiolabeling optimization of this novel PSMA-targeting ligand with ^123^I was performed by investigating the effect of the PSMA ligand amount, the reaction time, and the oxidizing agent amount.

**Figure 4:**
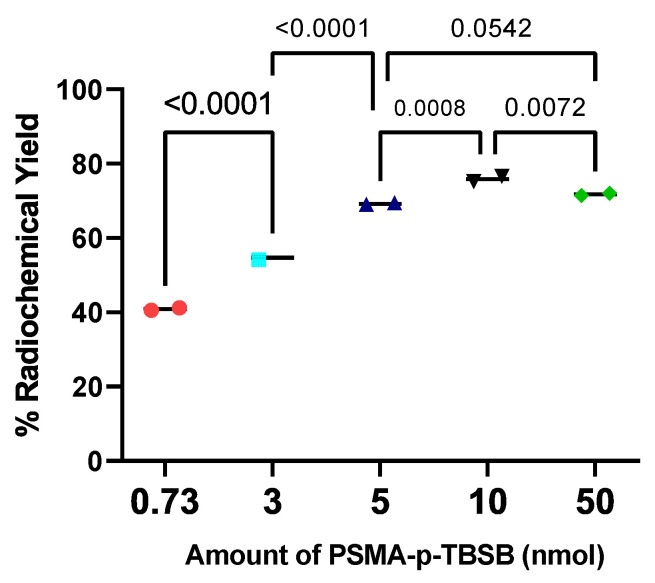
Radiochemical yield as a function of the amount of PSMA ligand. The comparison study was performed by an ANOVA test with Tukey’s post hoc analysis (95% confidence interval).

To study the effect of the PSMA ligand amount to the radiochemical yield, a fixed reaction time of 5 min and an oxidizing agent amount of 40 μg was used. The radiochemical yields (RCYs), determined by radio-iTLC as a function of PSMA ligand amount, are presented in Figure 4. The reaction was quite efficient at low amounts of PSMA ligand (0.73–5 nmol or 1–7 μg). Generally, it appeared that the use of a larger molar amount of PSMA ligand in the radiolabeling reaction improved the labeling yield. However, no increase was found, instead a significant decrease (*p =* 0.0072) in RCY occurred when the amount of PSMA ligand was increased from 10 nmol to 50 nmol. The radiochemical yield from the use of 10 nmol amount of PSMA ligand was 75.9 ± 1.0%.

**Figure 5:**
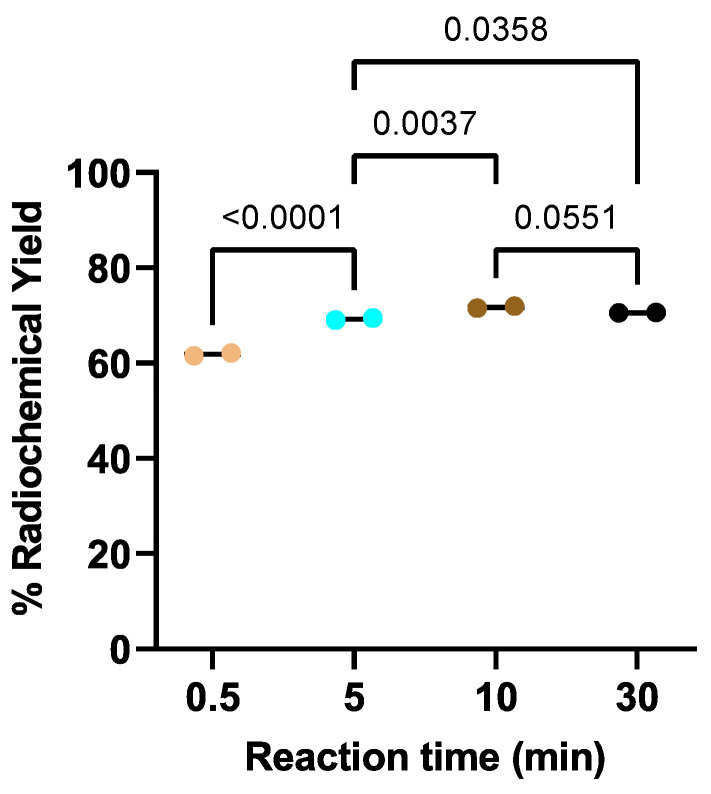
Radiochemical yield as a function of reaction time. The comparison study was performed by an ANOVA test with Tukey’s post hoc analysis (95% confidence interval).

Reaction time of the radiolabeling was studied using fixed amounts of the PSMA ligand (7 μg; 5 nmol) and the oxidizing agent (40 μg; 176 nmol). The 30 s reaction process produced a radiochemical yield of 61.9 ± 0.4%. An increase in the radiochemical yield occurred with the increase in reaction time until the use of a 10 min reaction time produced an RCY of 71.7 ± 0.3%. The radiochemical yield started to slightly decrease when the reaction time was extended to 30 min. The radiochemical yield as a function of reaction time is presented in Figure 5.

**Figure 6:**
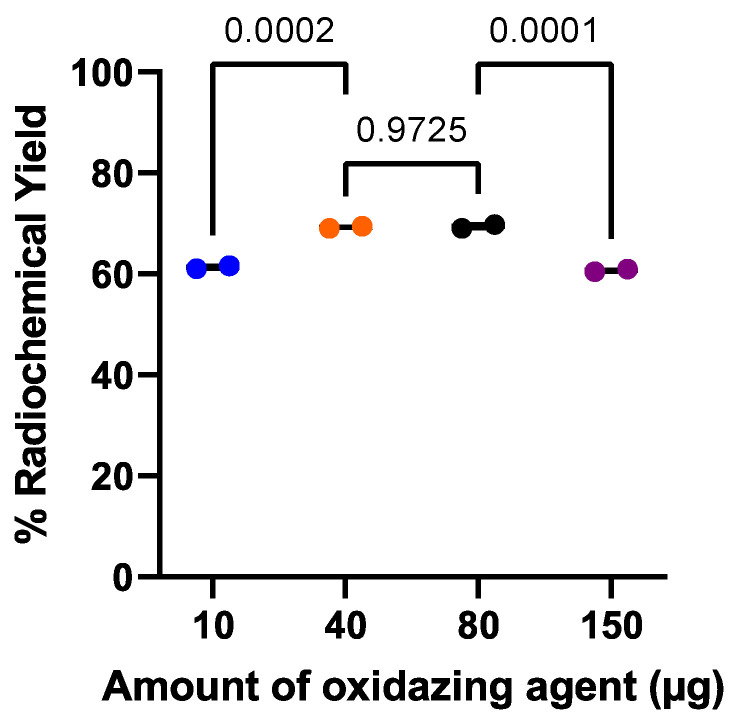
Radiochemical yield as a function of the oxidizing agent amount. The comparison study was performed by an ANOVA test with Tukey’s post hoc analysis (95% confidence interval).

The effect of the amount of oxidizing agent on the yield of [^123^I]PSMA-p-IB radiolabeling was studied as shown in Figure 6. The reactions were carried out using a fixed amount of PSMA ligand (5 nmol) and for a fixed time of 5 min. The data clearly show that increasing the amount of chloramine-T as an oxidizing agent from 10 μg to 40 μg significantly improved the yield from 61.3 ± 0.5% to 69.2 ± 0.2%. Increasing the amount of chloramine-T in the reaction to 80 μg did not increase the radiolabeling results; even further additions up to 150 μg resulted in significantly decreased radiolabeling results. The yields for each radiolabeling condition of PSMA-p-TBSB ligands by ^123^I are presented in Table 2.

**Figure 7:**
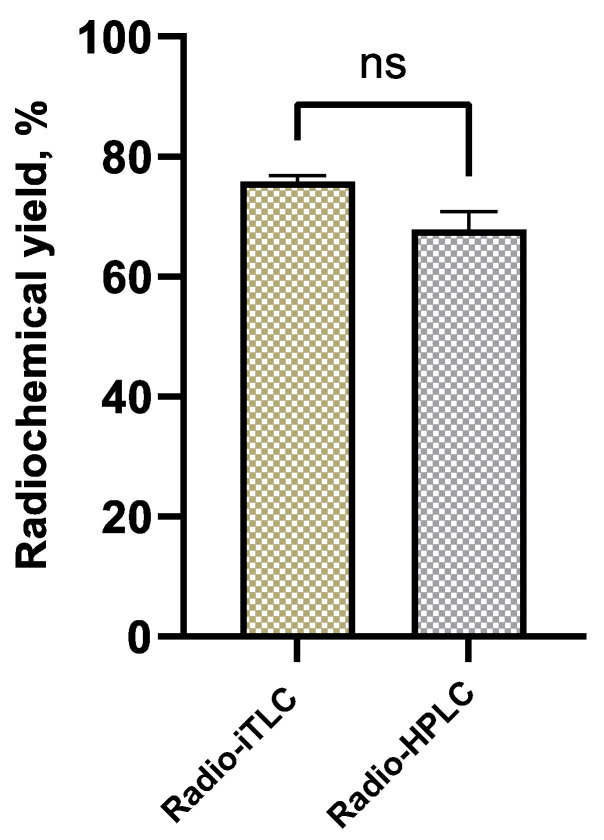
The radiochemical yields as denoted above are the radiochemical yields of [^123^I]PSMA-p-IB as analyzed by different methods. “ns” indicates a not significant difference by the two-tailed *t*-test (99% confidence interval).

Based on the results of the radiolabeling optimization study, the following labeling conditions were considered optimal: an amount of PSMA-p-TBSB ligand of 10 nmol, an amount of oxidizing agent of 40 μg, and a reaction time of 5 min. The [^123^I]PSMA-p-IB radiolabeling efficiency obtained under these conditions was 75.9 ± 1.0%. We also performed radio-HPLC analysis of the radiochemical yield to compare this with the optimum radiochemical yield as determined using the radio-iTLC-SG glass-fiber sheet. The radio-HPLC chromatograms of [^123^I]PSMA-p-IB without purification are displayed in Figure S1 (in Supplementary Materials). The results of the two methods (Figure 7) showed no significant difference with a 99% confidence interval (by two-tailed *t*-test; *p* < 0.01). The radio-iTLC of the [^123^I]PSMA-p-IB ligand based on the results of the radiolabeling optimization is shown in Figure S2 (in Supplementary Materials). As a comparison, we conducted a radiolabeling study of PSMA-m-STB with ^123^I to produce [^123^I]PSMA-m-IB (compound **24**) under the optimum radiolabeling conditions of [^123^I]PSMA-p-IB. The obtained radiochemical yield of [^123^I]PSMA-m-IB was 49.41%. We recognized that the [^123^I]PSMA-p-IB radiochemical yield was higher than [^123^I]PSMA-m-IB. Consequently, [^123^I]PSMA-p-IB was used as a model to perform the initial preclinical evaluations of this new PSMA-targeting ligand.

### 2.3. Radiochemical Purity and Shelf-life Stability

**Figure 8:**
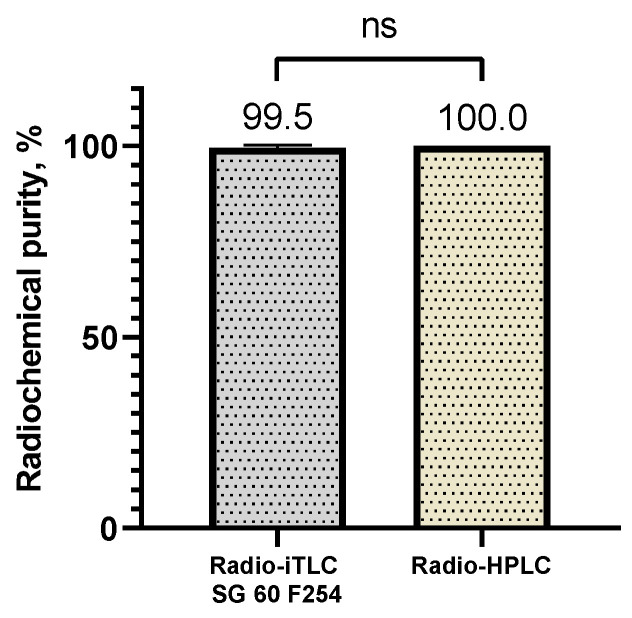
Radiochemical purity of [^123^I]PSMA-p-IB as analyzed by different methods. “ns” indicates a not significant difference by the two-tailed *t*-test (99% confidence interval).

After the radiolabeling process, [^123^I]PSMA-p-IB was separated from the impurities in the reaction mixture. The radiolabeled PSMA was purified using a Sep-Pak^®^ C18 cartridge. The quality control of [^123^I]PSMA-p-IB’s radiochemical purity was performed by radio-iTLC and radio-HPLC methods. The radio-iTLC and radio-HPLC chromatograms of the pure [^123^I]PSMA-p-IB are shown in Figure S3 (in Supplementary Materials). The radiochemical purity of the [^123^I]PSMA-p-IB obtained using the radio-iTLC method was 99.50 ± 0.7%, while radio-HPLC showed an [^123^I]PSMA-p-IB radiochemical purity of 100 ± 0.0%, which was not significantly different (at a 99% confidence level) from the radiochemical purity obtained by radio-iTLC. The radiochemical purities of [^123^I]PSMA-p-IB as analyzed by the different methods are shown in Figure 8.

After three days of storage at a temperature of −20 °C, the shelf-life stability of [^123^I]PSMA-p-IB in ethanol was investigated. Shelf-life stability needs to be evaluated because during storage, radiolysis may cause radiopharmaceuticals to decompose, resulting in radiochemical impurities. The results of the radio-HPLC analysis of [^123^I]PSMA-p-IB showed that 100% purity has not changed at all since the purification process in the previous 3 days. The radio-HPLC chromatogram of [^123^I]PSMA-p-IB after the 3-day storage period is presented in Figure S4 (in Supplementary Materials).

### 2.4. Lipophilicity

The lipophilicity of [^123^I]PSMA-p-IB was determined by its equilibrium distribution after thorough shaking in a two-phase system consisting of n-octanol and water. Small aliquots from both phases were collected and analyzed in an automated gamma-counter in order to calculate the distribution coefficients Log(D). The distribution coefficient Log(D) of the [^123^I]PSMA-p-IB was 0.99. The results obtained indicate that this ligand was lipophilic. The hydrophobic characteristics of this ligand was influenced by the linker structure, which was a dipeptide fragment containing the isotope ^123^I, two phenylalanine residues in the L-configuration, and the presence of chlorine substituents on the aromatic group of the ε-amino group of lysine [10].

### 2.5. In Vitro Cell-Binding Assay

**Figure 9:**
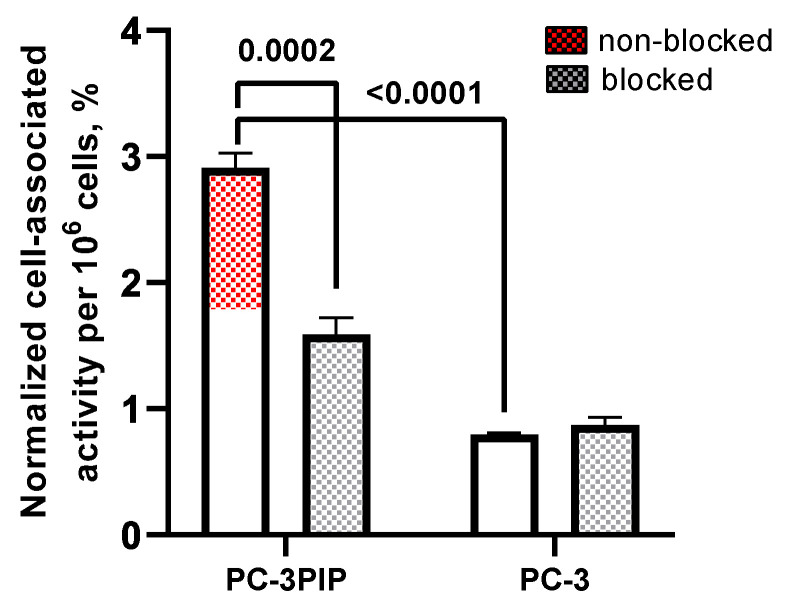
Binding specificity of [^123^I]PSMA-p-IB. For blocking, a 500-fold molar excess of non-labeled PSMA ligand was added to the blocked groups. The final concentration of radiolabeled compound was 1 nM.

An in vitro cell-binding test for [^123^I]PSMA-p-IB was performed using PC-3 PIP (PSMA-positive) and PC-3 (PSMA-negative) cell lines (Figure 9). The level of binding of [^123^I]PSMA-p-IB to PC-3 PIP was significantly (*p* < 0.0001; unpaired two-tailed *t*-test) higher than to PC-3 cells. To investigate the specificity of [^123^I]PSMA-p-IB toward the receptor, blocking of the receptors by adding a 500-fold molar excess of unlabeled PSMA ligand was performed. When the active site of PSMA was partially saturated with unlabeled PSMA ligand, positive PSMA-expressing PC-3 PIP cells showed a significant (*p* = 0.0002) decrease in [^123^I]PSMA-p-IB accumulation. On the other hand, there was no significant (*p* < 0.05) different between non-blocked and blocked negative PSMA-expressing PC-3 cells, which means that [^123^I]PSMA-p-IB demonstrated a slight non-specific accumulation by the negative PSMA-expressing PC-3 cells.

### 2.6. Binding Affinity

**Figure 10:**
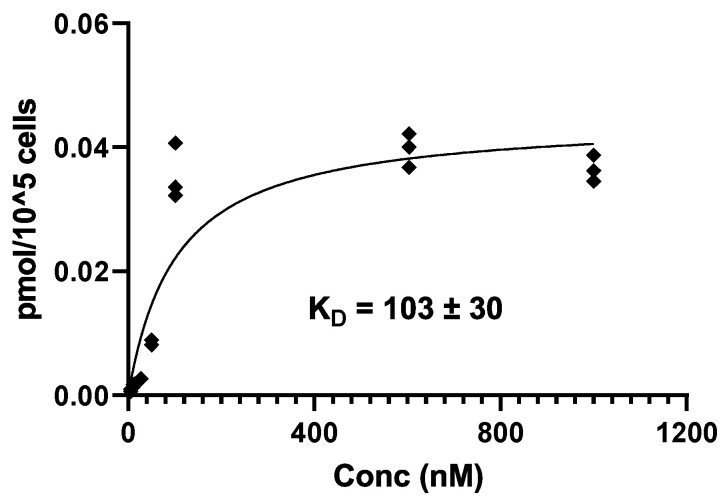
The saturation experiment of [^123^I]PSMA-p-IB on PC-3 PIP cells.

The binding affinity of [^123^I]PSMA-p-IB was evaluated using PC-3 PIP cells. The saturation experiment of [^123^I]PSMA-p-IB on PC-3 PIP cells is presented in Figure 10. The equilibrium dissociation constant (K_D_) for [^123^I]PSMA-p-IB was 103 ± 30 nM.

### 2.7. In Vivo Biodistribution

A comparative biodistribution study was conducted between [^123^I]PSMA-p-IB and [^177^Lu]Lu-PSMA-617 in normal mice. The [^177^Lu]Lu-PSMA-617 used was obtained at high radiochemical purity (>95%). The radio-iTLC and radio-HPLC analyses of the [^177^Lu]Lu-PSMA-617 yield are presented in Figure S5 (in Supplementary Materials).

**Figure 11:**
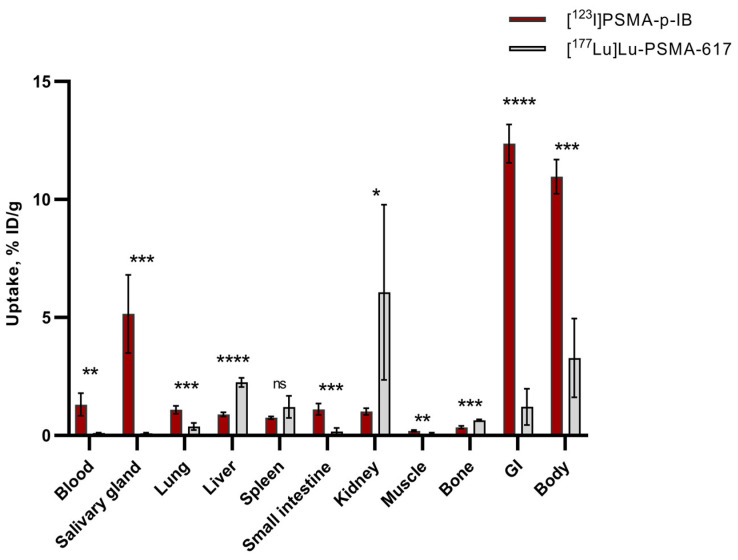
Biodistribution of [^123^I]PSMA-p-IB and [^127^Lu]Lu-PSMA-617 in CD1 mice 4 h post-injection (p.i.). Data for the gastrointestinal tract (including the contents) and the body are presented as the %ID per whole sample. ns indicates *p* > 0.05, * indicates *p* ≤ 0.05, ** indicates *p* ≤ 0.01, *** indicates *p* ≤ 0.001 and **** indicates *p* ≤ 0.0001. The comparison study was performed by unpaired 2-tailed *t*-tests (95% confidence interval).

The mice were sacrificed at 4 h post-injection (p.i.) by cervical dislocation. A side-by-side comparison of the biodistribution of [^123^I]PSMA-p-IB and [^177^Lu]Lu-PSMA-617 in normal mice is shown in Figure 11. Biodistribution of [^123^I]PSMA-p-IB at 4 h p.i. in CD1 mice reflects a low level of accumulation in almost all normal organs, except in the salivary gland, gastrointestinal tract, and the rest of the body. There were significant differences between the uptake of [^123^I]PSMA-p-IB and [^177^Lu]Lu-PSMA-617 in several normal organs. Low accumulation of both ligands was detected in muscle and bone. However, it was determined that the uptake of [^177^Lu]Lu-PSMA-617 in muscle (0.1 ± 0.1 %ID/g) was noticeably lower than that of [^123^I]PSMA-p-IB (0.2 ± 0.0 %ID/g). Meanwhile, [^123^I]PSMA-p-IB accumulation in bone was significantly lower than [^177^Lu]Lu-PSMA-617 accumulation, being 0.3 ± 0.1 and 0.6 ± 0.1 %ID/g, respectively. The activity of [^123^I]PSMA-p-IB in blood was 1.3 ± 0.5 %ID/g. This uptake was significantly higher than the [^177^Lu]Lu-PSMA-617 uptake, which was 0.1 ± 0.0 %ID/g. The activity in the blood for [^123^I]PSMA-p-IB experiment was associated with PSMA-p-TBSB labeled with ^123^I, which is non-residualizing and is thought to result in the rapid excretion of radiometabolites. Additionally, this ligand lipophilicity might lead it to bind to proteins in the blood. An [^123^I]PSMA-p-IB accumulation of 5.2 ± 1.7 %ID/g was observed in the salivary glands. This value was clearly higher than the [^177^Lu]Lu-PSMA-617 accumulation in the same organ of 0.1 ± 0.0 %ID/g. This [^123^I]PSMA-p-IB accumulation could be due to radioiodine catabolites. The activity in the whole gastrointestinal tract (with contents) and small intestine for [^123^I]PSMA-p-IB was 12.4 ± 0.8 and 1.1 ± 0.2 %ID/g, respectively. Meanwhile, the activity of [^177^Lu]Lu-PSMA-617 in the whole gastrointestinal tract (with contents) and small intestine was 1.2 ± 0.8 and 0.2 ± 0.1 %ID/g, respectively.

Remarkable results were observed whereby the accumulation of [^123^I]PSMA-p-IB (0.9 ± 0.1 %ID/g) in the liver was substantially lower than that of [^177^Lu]Lu-PSMA-617 (2.3 ± 0.2 %ID/g). In addition, there was also a difference in the accumulation of radioligands in the kidney organ, where the uptake of [^123^I]PSMA-p-IB (1.0 ± 0.1 %ID/g) was significantly less than that of [^177^Lu]Lu-PSMA-617 (6.1 ± 3.7 %ID/g).

## 3. Discussion

A number of publications have confirmed that modification of the DCL ligand structure affects its affinity for PSMA. We assume that modifying the linker in DCL with a dipeptide fragment containing two phenylalanine residues improves the affinity properties of the PSMA ligand by taking advantage of the hydrophobic interaction with the S1 hydrophobic pocket of PSMA [22,23]. Other evidence showing that modification of the aromatic moiety of the ε-amino group of lysine with halogen has an effect on the affinity [10]. Thus, for the synthesis, we choose a novel DCL urea-based PSMA-ligand with a chlorine-substituted aromatic fragment at the lysine ε-nitrogen atom and a dipeptide including two phenylalanine residues in the L-configuration as the peptide fragment of the linker.

The assembly of the Phe(L)-Phe(L) peptide sequence to obtain highly specific PSMA vectors was carried out using SPPS on the cross-linked styrene-divinylbenzene copolymer matrix (2-CTC resin) (Scheme 2). The 2-CTC resin was chosen for the solid-phase synthesis as it allows the concept of Fmoc\Bu^t^ SPPS to be applied while minimizing possible side reactions. In addition, it allows acid-labile functional groups to be kept intact, since the removal of amino acid sequences from the resin proceeds under mild conditions (in this case, DCM/TFA—99.25%/0.75% *v*/*v*; the reaction does not affect the acid-labile COOBu^t^ groups) [28].

The preparation of NHS-activated esters of the prosthetic groups p-STBSB and m-STBSB (Scheme 3) was carried out similarly to the method described in the article [30], with some modifications. The most significant of these modifications were (1) the method of the compound **13** synthesis, which allowed the yield to be significantly increased; and (2) the methods of isolating compounds **13**–**17**.

At the final stage of the synthesis (Scheme 4), the protective *tert*-butyl groups of compound **11** were removed. According to the literature data [31], the Bu*^t^* group can be removed by TFA (or HCl in AcOH or in dioxane) with acidolysis of the carboxylate esters -COOBu*^t^*. When TFA acts on *tert*-Bu-containing compounds, *tert*-Bu^+^ cations are formed, which are capable of being captured by TFA to form the strong alkylating agent CF_3_COOBu*^t^*, which can cause side alkylation reactions. A possible way to avoid alkylation side reactions is to add scavenger molecules to the reaction mixture. The best absorbers of *tert*-butyl cations are trialkylsilanes (R_3_SiH), such as triethyl- and triisopropylsilane (TIPS). Water is also an effective scavenging agent for *tert*-butyl cations [32].

It should be noted that the possible side processes can not only be alkylation and acylation reactions of the peptide sequence but also reactions of intramolecular condensation of the DCL ligand, leading to the formation of three isomeric five-membered heterocycles [33].

Compounds containing non-radioactive iodine (compound **21** and **22**) were obtained in the study as standard compounds for comparison and characterization, and these are therefore applicable regardless of other techniques used. Meanwhile, in studying these novel ligands as candidates for radiopharmaceuticals, the radioiodination process was carried out through the electrophilic radioiodination method in the presence of chloramine-T as an oxidizing agent.

As a model for studying the radiolabeling optimization and initial preclinical evaluation of these new ligands as radiopharmaceutical candidates, a ligand with a prosthetic group containing 4-tributylstannyl was used. Radiolabeling was accomplished through an electrophilic radioiodination reaction in the presence of [^123^I]NaI and chloramine-T. The electrophilic species (HO*I, H_2_O*I), generated from radioiodide and the oxidant, react directly with the aromatic moiety of the prosthetic group to be labelled [34]. The iododestannylation reactions was used because they usually give high product yields [35]. Chloramine-T was used as an oxidizing agent in this experiment because it allows radiolabeling to occur under mild conditions, so the peptides will not be damaged due to the influence of the reaction temperature.

The study on the effect of the amount of PSMA ligand on radiolabeling yields in general indicates that the use of a larger molar amount of PSMA-p-TBSB in a radiolabeling reaction increases the labeling yield until it reached its optimum point when using 10 nmol of the PSMA-p-TBSB ligand. Findings from time-effect studies demonstrate that when using oxidizing agents in the iododestannylation process of a peptide, the correct reaction time is critical. Longer reaction times can diminish the RCY due to undesired overoxidation, which results in chlorination and oxidative denaturation [35]. In addition, the optimal yield of the [^123^I]PSMA-p-IB ligand is dependent upon an appropriate amount of chloramine-T as the oxidizing agent in this radiolabeling procedure. Elemental iodine is formed by the oxidation of sodium iodide with oxidizing agents to produce H_2_OI^+^ and HOI from sodium iodide. An excess amount of chloramine-T in the reaction causes a decrease in yield, which may be due to the formation of undesirable oxidative side polymerization [35]. The combination of 10 nmol PSMA ligand and 40 μg chloramine-T and a reaction time of 5 min was considered the optimum condition in the PSMA-p-TBSB radiolabeling process with ^123^I. The labeling yield obtained under this method was 75.9 ± 1.0%.

Following the purification procedure, [^123^I]PSMA-p-IB with a high radiochemical purity of >99.50% was achieved. This value was validated utilizing radio-iTLC and radio-HPLC techniques. During storage, radiolysis may cause radiopharmaceuticals to decompose, resulting in radiochemical impurities. Radiolysis generates free radicals, which is one of the primary causes of radiolabeled preparation degradation. Radiolysis can result in the breakdown of chemical bonds between the radionuclide and the molecule, leading to the formation of radiochemical impurities [36]. Shelf-life stability needs to be evaluated because an impurity can become the main tracer circulating in the bloodstream, resulting in excessive body background, obscuring the diagnosis of disease and increasing the patient’s radiation exposure. The investigation revealed that the purity of [^123^I]PSMA-p-IB did not change at all during a shelf-life of three days following the purification procedure.

The major purpose of the in vitro investigation was the evaluation of the ability of [^123^I]PSMA-p-IB to bind to prostate cancer cells dependent on PSMA surface representation. The binding of [^123^I]PSMA-p-IB to PSMA-expressing PC-3 PIP cells was receptor-mediated. The equilibrium dissociation constant was still at the nanomolar level. This affinity measurement revealed that [^123^I]PSMA-p-IB binds to PSMA-expressing cells with considerable affinity.

There were numerous variables that could have impacted the acquisition of this K_D_ value. The introduction of hydrophobic functional groups in the linkers in these new ligands is thought to increase the binding affinity of PSMA in addition to the binding characteristics of glutamate or glutamate-like residues to the S1′ pocket and the simultaneous inhibition of the zinc binuclear active site, which are indispensable and minimum requirements for PSMA inhibitors. We suspect that the presence of chlorine substituents on the aromatic group of the ε-amino lysine group increased the lipophilic properties of the ligand, which could be advantageous when interacting with the S1 hydrophobic pocket of PSMA. Chlorine has a higher π value relative to hydrogen [37]. In addition, the chemical structure size of this ligand that interact with the S1 hydrophobic pocket is crucial. [^123^I]PSMA-p-IB contains quite large substituents at the linker, namely two phenylalanine residues in the L-configuration and chlorine on the aromatic group of the ε-amino group of lysine. As reported by Lundmark et al., the size of the substituent in the linker in a PSMA ligand has a significant effect on the value of K_D_ [38].

To examine the biodistribution characteristics of this new ligand, the biodistribution of [^123^I]PSMA-p-IB in normal mice was compared with that of [^177^Lu]Lu-PSMA-617, which has demonstrated promising results in clinical studies. The uptake of [^123^I]PSMA-p-IB in normal organs, except in the salivary gland, gastrointestinal tract, and body, were low. At 4 h after injection, the activity of [^123^I]PSMA-p-IB in the blood and salivary glands was significantly higher than the [^177^Lu]Lu-PSMA-617 uptake. This value was correlated with PSMA-p-IB that had been ^123^I-labeled, which is non-residualizing and is thought to cause radiometabolites to be excreted quickly. This advantage has the potential to be exploited to increase the ratio between activity concentrations in tumors and normal organs. The lipophilic nature of this ligand may also predispose it to bind to proteins in the blood. Meanwhile, the high [^123^I]PSMA-p-IB accumulation in salivary glands could be due to radioiodine catabolites. Radioactive accumulation was observed in several organs capable of concentrating iodine by a Na/I symporter. This experiment was carried out without giving potassium iodide to the drinking water of mice in the days before the experiment. It is useful for evaluating the true biodistribution. The network that expresses the Na/I symporter, which plays a role in the processing of radioiodinated peptide metabolites, was not blocked, so this might have affected the uptake value of radioiodinated peptide in this organ [39]. The accumulation of [^123^I]PSMA-p-IB in bone tissue was lower than the accumulation of [^177^Lu]Lu-PSMA-617 in bone. This phenomenon can be explained by the fact that Lu^3+^, as a lanthanoid, exhibits some chemical similarities with Ca^2+^, which has a high uptake in bone tissue. Despite the presence of endogenous PSMA expression in the kidney, the [^123^I]PSMA-p-IB biodistribution pattern suggested a low uptake in this organ. This value was significantly lower than that of [^177^Lu]Lu-PSMA-617. It was also discovered that the accumulation of [^123^I]PSMA-p-IB in the liver was significantly less than that of [^177^Lu]Lu-PSMA-617. According to other previous studies, high uptake in these organs is a known concern. Hillier et al. [40] have also attempted to develop radiolabeled ligands with prosthetic groups as molecular imaging pharmaceuticals for prostate cancer, namely [^123^I]MIP-1072 and [^123^I]MIP-1095. Those ligands have high affinity toward PSMA-expressing cells. However, the results of preclinical evaluation of the two ligands showed high accumulation in the liver and kidney of NCr nude mice bearing LNCaP xenografts. In the liver, the accumulation of [^123^I]MIP-1072 and [^123^I]MIP-1095 at 4 h after injection was 2.17 ± 0.67 and 7.82 ± 1.01 %ID/g, respectively. Meanwhile in the kidney, the accumulation of [^123^I]MIP-1072 and [^123^I]MIP-1095 after 4 h was 35.7 ± 18.7 and 77.5 ± 16.5 %ID/g, respectively [40]. The low [^123^I]PSMA-p-IB accumulation in the kidney and liver is an encouraging finding. The accumulation of [^123^I]PSMA-p-IB in the liver and kidney at 4 h p.i. was 0.9 ± 0.1 and 1.0 ± 0.1 %ID/g, respectively. This will undoubtedly be a benefit of the [^123^I]PSMA-p-IB ligand when used as a diagnostic for prostate cancer, as the location of the kidney is close to the prostate gland, thereby optimizing the prostate image during imaging due to the low accumulation of [^123^I]PSMA-p-IB in the kidney. It was therefore hypothesized that [^123^I]PSMA-p-IB provides low retention of activity in excretory organs.

PSMA ligands can potentially be used for other therapeutic strategies such as image-guided surgery of prostate cancer lesions [41]. The gamma-emitting [^123^I]PSMA-p-IB ligand can be employed as a radiotracer for PSMA radio-guided operation because of its advantageous half-life of 13 h. Comparable to medical targeted therapy, PSMA radio-guided surgery facilitates targeted molecular surgery, as it allows for the specific intraoperative detection of PSMA-expressing prostate-cancer deposits, especially for the intraoperative detection of atypically positioned lesions and small subcentimeter metastatic lymph nodes [41,42]. We propose that the [^123^I]PSMA-p-IB ligand might also have implications for radio-guided surgery, and a feasibility study of radio-guided surgery with [^123^I]PSMA-p-IB should be carried out in the future.

## 4. Materials and Methods

All starting compounds are commercially available reagents. The initial stages of the synthesis of vector fragments **1**–**5** (Scheme 1) were carried out using methods previously developed by our scientific group [26]. For compounds **12** and **15**, the reaction and purification conditions are given in [30]. ^1^H NMR was measured with a Bruker Avance spectrometer (Bruker Corporation, Billerica, MA, USA) operating at 400 MHz for ^1^H using CDCl_3_ and DMSO-d_6_ as solvents. Chemical shifts are reported in δ units to 0.01 ppm precision, with coupling constants reported to 0.1 Hz precision using residual solvent as an internal reference. ^13^C NMR was measured with a Bruker Avance spectrometer operating at 100 MHz using DMSO-d_6_ as a solvent. Chemical shifts are reported in δ units to 0.1 ppm precision using residual solvent as an internal reference. NMR spectra were processed and analyzed using Mnova software 5.2.5-5780, Mestrelab Research, Santiago de Compostela, Spain). High-resolution mass spectra were recorded on the Orbitrap Elite high-resolution mass spectrometer. Solutions of samples in acetonitrile with 1% formic acid were introduced into the ionization source by electrospray. For HPLC analysis, a system with a Shimadzu Prominence LC-20 column (Shimadzu, Kyoto, Japan) and a convection fraction collector connected to a single quadrupole mass spectrometer Shimadzu LCMS-2020 (Shimadzu, Kyoto, Japan) with dual ionization source DUIS-ESI-APCI were used. The analytical and preparative column was Phenomenex Luna 3 μm C18 100 Å. Preparative chromatographic separation of substances was carried out using the INTERCHIM puriFlash 430 chromatograph (INTERCHIM, Montluçon, France).

The radionuclidic purity of ^123^I was measured by high-purity Canberra Ge detector (type GC1020, diameter 46.5 mm, and length 32 mm) coupled to a multi-channel analyzer Canberra InSpector 2000 and the acquisition/analyzing software Genie 2000 (Canberra Industries Inc., Meriden, CT, USA). The detector was previously calibrated using a standard point source. Solutions with activity values of more than 25 MBq were measured by an ionization chamber using the dose calibrator RIS-1A, Amplituda, Saint Petersburg, Russia. Radio-iTLC was performed using the miniGITA Single (Elysia Raytest, Straubenhardt, Germany) iTLC scanner. The in vitro and vivo test samples were measured by a thallium-activated sodium iodide (NaI(Tl)) detector using the automated gamma-counter Wizard 1480 (Pelkin Elmer, Waltham, MA, USA).

Purification of the radiolabeling yield was performed with a Sep-Pak^®^ C18 cartridge (catalogue number WAT036815, Waters, Milford, MA, USA). Thin-layer chromatography (TLC) analysis was performed using an iTLC glass microfiber chromatography sheet impregnated with a silica gel (iTLC SG fiber sheet) (Agilent Technologies, Inc., Folsom, CA, USA) and iTLC silica gel 60 F_254_ aluminium plates (iTLC SG 60 F_254_) (Merck KGaA, Darmstadt, Germany)

Data on radiolabeling, binding specificity, and biodistribution were analyzed by ANOVA tests with Tukey’s post hoc analysis and two-tailed *t*-tests to determine any significant difference using GraphPad Prism (version 9.5.0 for Windows; GraphPad Software, La Jolla, CA, USA).

### 4.1. Chemistry

**Compound 6.** DIPEA (1.4 eq; 244 μL; 1.4 mmol) and succinic anhydride (1.02 eq; 102 mg; 1.02 mmol) were added to a solution of compound **5** (1 eq; 725 mg; 1.0 mmol) in 20 mL of DCM. The mixture was stirred for 12 h, then MeOH (2 eq.) was added, and the resulting mixture was stirred for 1 h. Then, the solvent was removed under reduced pressure, and the residue was dissolved in DCM and extracted, first with 0.1 M HCl (2 × 30 mL) and then with brine (2 × 30 mL). Then, the organic fraction was dried over Na_2_SO_4_ and concentrated under reduced pressure to finally obtain compound **6** as a yellow oil (801 mg, yield 97%).

^1^HNMR (400 MHz, DMSO-*d6*, δ): 12.06 (br.s., 1H, X4C(O)OH), 7.81 (t, J = 5.2Hz, *m*) and 7.77 (t, J = 5.2Hz, *n*) (1H, X3NHk, *m + n*, *m*/*n* = 3/2), 7.40 (t, J = 7.7Hz, X8He, *n*), 7.37–7.27 (m, X8Hd + X8He(*m*)), 7.26–7.21 (m, 1H, X8Ht, *m + n*), 7.19–7.10 (m, 1H, X8Hg, *m + n*), 6.34–6.20 (m, 2H, K2NH + E1NH, *m + n*), 4.56 (s, *n*) and 4.48 (s, *m*) (2H, X8Ha, *m + n*, *m*/*n* = 3/2), 4.07–4.00 (m, 1H, E1Ha, *m + n*), 4.00–3.90 (m, 1H, K2Ha, *m + n*), 3.22 (t, J = 7.3 Hz, *n*) and 3.19 (t, J = 7.3Hz, *m*) (2H, K2He, *m + n, m*/*n* = 3/2), 3.01 (q, J = 6.4, 12.7 Hz, *m*) and 2.96 (q, J = 6.4, 12.7 Hz, *n*) (2H, X3He, *m + n*, *m*/*n* = 3/2), 2.44–2.38 (m, 2H, X4Hb, *m + n*), 2.36 (t, J = 7.4Hz, X3Ha, *m*), 2.31–2.25 (m, 2H, X4Ha, *m + n*), 2.25–2.15 (m, E1Hg + X3Ha(*n*)), 1.91–1.80 (m, 1H, E1Hb(a)), 1.72–1.63 (m, 1H, E1Hb(b)), 1.63–1.56 (m, 1H, K2Hb(a)), 1.40–1.35 (m, 27H, tBu), 1.56–1.15 (m, 11H, K2Hb(b) + X3Hb + X3Hd+ K2Hd + K2Hg + X3Hg, *m + n*). The ^1^H NMR spectrum of compound **6** is presented in Figure S6 (in Supplementary Materials).

^13^C NMR (100 MHz, DMSO-*d_6_*, δ): 173.93 (X4Cg), 172.26 (K2C(*n*)), 172.23 (K2C(*m*)), 172.22 (X3C(*n*)), 172.19 (X3C(*m*)), 171.95 (E1C), 171.47 (E1Cg), 170.76 (X4C(*m*)), 170.73 (X4C(*n*)), 157.18 (U(*m*)), 157.16 (U(*n*)), 141.20 (X9Cb(*m*)), 140.80 (X9Cb(*n*)), 133.45 (X9Ce(*n*)), 133.10 (X9Ce(*m*)), 130.63 (X9Cd(*n*)), 130.26 (X9Cd(*m*)), 127.24 (X9Ct(*m*)), 127.17 (X9Ck(*n*)), 126.88 (X9Ck(*m*)), 126.34 (X9Ct(*n*)), 126.08 (X9Cg(*m*)), 124.99 (X9Cg(*n*)), 80.59 (E1tBu), 80.42 (K2tBu(*m*)), 80.33 (K2tBu(*n*)), 79.77 (E1dtBu), 53.01 (K2Ca(*n*)), 52.88 (K2Ca(*m*)), 52.20 (E1Ca(*m*)), 52.18 (E1Ca(*n*)) 49.63 (X9Ca(*n*)), 47.11 (X9Ca(*m*)), 46.83 (K2Ce(*m*)), 45.20 (K2Ce(*n*)), 38.49 (X3Ce(*m*)), 38.43 (X3Ce(*n*)), 32.34 (X3Ca(*n*)), 31.95 (X3Ca(*m*)), 31.83 (K2Cb), 30.93 (E1Cg), 30.06 (X4Ca), 29.25 (X4Cb), 29.13 (X3Cd(*m*)), 29.04 (X3Cd(*n*)), 27.75 (tBuE1), 27.69 (K2Cd(*m*)), 27.66 (tBuK2), 27.64 (tBuE1g + E1Cb), 26.72 (K2Cd(*n*)), 26.23 (X3Cg(*m*)), 26.15 (X3Cg(*n*)), 24.76 (X3Cb(*m*)), 24.63 (X3Cb(*n*)), 22.45 (K2Cg(*n*)), 22.27 (K2Cg(*m*)). The ^13^C NMR spectrum of compound **6** is presented in Figure S7 (in Supplementary Materials).

ESI-MS C_41_H_65_ClN_4_O_11_: *m*/*z* calcd. for [M + H^+^]^+^: 825.44; found: 825.45. The ESI-MS spectrum of compound **6** is presented in Figure S8 (in Supplementary Materials).

**Compound 8. *Activation of 2-CTC***. The suspension of 2-CTC (1 eq.; 1 g; 1.2–1.4 mmol/g; 100–200 mesh) in DCM (10 mL) was stirred for 10 min; after that, the mixture was purged with Ar, then SOCl_2_ (3 eq.; 305 μL; 4.2 mmol) was added dropwise, and then DMF (16 μL; 5% *v*/*v* to SOCl_2_) was added and stirred at 40 °C for 4 h. After that, the resin was filtered off and transferred to a polypropylene reactor, washed with DMF (3 × 10 mL, 1 min) and DCM (3 × 10 mL, 1 min).

***The addition of FmocPhe(L)-OH***. To the mixture of CTC-2 (1 eq.; 1 g; 1.2–1.4 mmol/g; 100–200 mesh) in DMF (10 mL), FmocPhe(L)-OH (2 eq.; 1.085 g; 2.8 mmol) and DIPEA (10 eq.; 2.44 mL; 14 mmol) were added, and the mixture was stirred for 2 h. Then the resin was filtered off, washed with MeOH (3 × 10 mL, 5 min), DCM (3 × 10 mL, 1 min), DMF (3 × 10 mL, 1 min), and DCM (3 × 10 mL, 1 min).

***Fmoc-deprotection***. FmocPhe(L) on a 2-CTC resin (1 eq.) was washed with DMF (2 × 15 mL, 1 min), then 4-methylpiperidine in DMF (20%/80% *v*/*v*, 15 mL) was added, and the mixture was stirred for 15 min. After that, the resin was filtered off, washed with DMF (3 × 15 mL, 1 min), and 4-methylpiperidine in DMF (20%/80% *v*/*v*, 15 mL) was added and the mixture stirred for 15 min. After the resin was filtered off, it was washed with DMF (3 × 15 mL, 1 min) and DCM (3 × 15 mL, 1 min).

***The addition of FmocPhe(L)-OH***. To the mixture of NH_2_-Phe(L) on a CTC-2 resin (1 eq.) in DMF (15 mL), FmocPhe(L)-OH (2 eq.; 1.085 g; 2.8 mmol), HOBt (0.5 eq.; 95 mg; 0.7 mmol), HBTU (2 eq.; 1.062 g; 2.8 mmol) and DIPEA (3 eq.; 0.73 mL; 4.2 mmol) were added, and the mixture was stirred for 2 h. Then the resin was filtered off and washed with DMF (3 × 15 mL, 1 min) and DCM (3 × 15 mL, 1 min).

***Fmoc-deprotection***. Fmoc-Phe(L)Phe(L) on a CTC-2 resin (1 eq.) was washed with DMF (2 × 15 mL, 1 min), then 4-methylpiperidineine in DMF (20%/80% *v*/*v*, 15 mL) was added and the mixture was stirred for 15 min. Then, the resin was filtered off and washed with DMF (3 × 15 mL, 1 min). 4-methylpiperidine in DMF (20%/80% *v*/*v*, 15 mL) was added, and the mixture was stirred for 15 min. After the resin was filtered off, it was washed with DMF (3 × 15 mL, 1 min) and DCM (3 × 15 mL, 1 min). Thus, the NH_2_-Phe(L)Phe(L) dipeptide was obtained on a 2-CTC resin (~1.4 mmol).

**Compound 9.** To the NH_2_-Phe(L)Phe(L) dipeptide on a 2-CTC resin (1 eq; 0.54 mmol) in 7 mL DMF, compound **6** (1.2 eq; 535 mg; 0.648 mmol), HOBt (0.5 eq; 37 mg; 0. 27 mmol), HBTU (2 eq; 410 mg; 1.08 mmol), and DIPEA (3 eq; 282 μL; 1.62 mmol) were added. The mixture was stirred for 4 h. The solvent was removed by filtration, and the resin was washed three times with DMF (7 mL), three times with DCM (7 mL), then dried of traces of solvent. A DCM/TFA mixture (99.25%/0.75%, 11 mL) was added to the resin and left stirring for 15 min, after which the resin was filtered off from the solution. The solvent was removed under reduced pressure, and the residue was re-evaporated with DCM and then purified by column chromatography (Puriflash on a PF-15C18AQ-F0025 column (15 μ 40 g): H_2_O (80%)/MeCN (20%) => H_2_O (0%)/MeCN (100%) for 15 min followed by MeCN (100%) for 5 min. Compound **9** was obtained as a white amorphous substance (460 mg, 76% yield).

^1^HNMR (400 MHz, DMSO-*d6*, δ): 12.71 (br.s., 1H, F6COOH), 8.30–8.22 (br.d., 1H, F5NH*mn*), 8.09–8.03 (br.d., 1H, F6NH*mn*), 7.79 (t, J = 5.4 Hz, *m*) and 7.75 (t, J = 5.4 Hz, *n*) (1H, X3NHk), 7.40 (t, J = 7.7Hz, X8Hd*n*), 7.36–7.29 (m, X8He*n* + X8Hd*m* + X8He*m*), 7.29–7.08 (m, 12H, F6He + F6Hd + X8Ht*mn* + F5He + F6Hk + F5Hk + F5Hd + X8Hg*mn*), 6.37–6.18 (m, 2H, K2NH + E1NH, *m* + *n*), 4.55 (s, X8Ha*n*), 4.53–4.44 (m, F6Ha + X8Ha*m*), 4.44–4.36 (m, 1H, F5Ha), 4.08–3.99 (m, 1H, E1Ha), 3.99–3.90 (m, 1H, K2Ha), 3.22 (t, J = 7.3 Hz, *n*) and 3.17 (t, J = 7.3 Hz, *m*) (2H, K2He), 3.11–3.02 (m, 1H, F6Hb(a)), 3.02–2.90 (m, 4H, F6Hb(b) + X3He*mn* + F5Hb(a)), 2.71–2.60 (m, 1H, F5Hb(b)), 2.35 (t, J = 7.4 Hz, X3Ha*m*), 2.30–2.10 (m, X4Hb*mn* + E1Hg + X4Ha*mn* + X3Ha*n*), 1.92–1.80 (m, 1H, E1Hb(a)), 1.71–1.62 (m, 1H, E1Hb(b)), 1.62–1.54 (m, 1H, K2Hb(a)), 1.54–1.10 (m, 11H, X3Hb + K2Hb(b) + X3Hd + K2Hd + K2Hg + X3Hg, *m* + *n*), 1.40–1.34 (m, 27H, tBu). The ^1^H NMR spectrum of compound **9** is presented in Figure S9 (in Supplementary Materials).

^13^C NMR (100 MHz, DMSO-*d_6_*, δ): 172.77 (F6C), 172.25 (K2C(*n*)), 172.21 (K2C(*m*)), 172.14 (X3C(*n*)), 172.12 (X3C(*m*)), 171.93 (E1C), 171.45 (E1Cd), 171.42 (X4Cg(*mn*)), 171.37 (F5C(*mn*)), 171.16 (X4C(*m*)), 171.15 (X4C(*n*)) 157.15 (U(*m*)), 157.14 (U(*n*)), 141.19 (X8Cb(*m*)), 140.78 (X8Cb(*n*)), 138.13 (F5Cg), 137.57 (F6Cg), 133.44 (X8Ck(*n*)), 133.09 (X8Ck(*m*)), 130.61 (X8Cd(*n*)), 130.25 (X8Cd(*m*)), 129.18 (F6Cd + F5Cd), 128.23 (F6Ce), 127.99 (F5Ce), 127.22 (X8Ct(*m*)), 127.16 (X8Ce(*n*)), 126.87 (X8Ce(*m*)), 126.45 (F6Ck), 126.32 (X8Ct(*n*)), 126.18 (F5Ck), 126.07 (X8Cg(*m*)), 124.96 (X8Cg(*n*)), 80.58 (E1tBu), 80.41 (K2tBu(*m*)), 80.32 (K2tBu(*n*)), 79.76 (E1dtBu), 53.67 (F5Ca), 53.63 (F6Ca), 53.00 (K2Ca(*n*)), 52.87 (K2Ca(*m*)), 52.19 (E1Ca), 49.61 (X8Ca(*n*)), 47.10 (X8Ca(*m*)), 46.80 (K2Ce(*m*)), 45.20 (K2Ce(*n*)), 38.53 (X3Ce(*m*)), 38.47 (X3Ce(*n*)), 37.34 (F5Cb), 36.61 (F6Cb), 32.33 (X3Ca(*n*)), 31.95 (X3Ca(*m*)), 31.83 (K2Cb), 30.93 (E1Cg), 30.82 (X4Ca), 30.75 (X4Cb), 29.12 (X3Cd(*m*)), 29.02 (X3Cd(*n*)), 27.75 (tBuE1), 27.66 (tBuK2 + K2Cd(*m*)), 27.63 (tBuE1d + E1Cb), 26.72 (K2Cd(*n*)), 26.29 (X3Cg(*m*)), 26.19 (X3Cg(*n*)), 24.76 (X3Cb(*m*)), 24.62 (X3Cb(*n*)), 22.44 (K2Cg(*n*)) 22.27 (K2Cg(*m*)). The ^13^C NMR spectrum of compound **9** is presented in Figure S10 (in Supplementary Materials).

ESI-MS C_59_H_83_ClN_6_O_13_: m/z calcd. for [M + H^+^]^+^: 1119.58; found: 1119.45. The ESI-MES spectrum of compound **9** is presented in Figure S11 (in Supplementary Materials).

HRMS (*m*/*z*, ESI): calcd. for C_59_H_83_ClN_6_O_13_–[M + H^+^]^+^: 1119.5779; found: 1119.5746. The HRMS spectrum of compound **9** is presented in Figure S12 (in Supplementary Materials).

**Compound 10.** To a solution of compound **9** (1 eq.; 300 mg; 0.268 mmol) in 10 mL of DMF, TFA*NH_2_(CH_2_)_3_NHFmoc (1.1 eq.; 121 mg; 0.294 mmol), DIPEA (2.5 eq.; 117 μL; 0.67 mmol) in 10 mL DMF, followed by HOBt (1 eq.; 36 mg; 0.268 mmol) and HBTU (1.5 eq.; 152 mg; 0.402 mmol), were added. The mixture was stirred for 12 h under an inert atmosphere. The solvent was then removed under reduced pressure and the residue was re-evaporated twice with DCM and dissolved in 30 mL of DCM. The extraction was then carried out, first with H_2_O (2 × 30 mL) and then with saturated NaCl solution (2 × 30 mL). Then, the organic fraction was dried over Na_2_SO_4_, the solvent was removed, and the residue was purified using a column chromatography method (Puriflash on a column (15 μ 40 g)): DCM (100%)/MeOH (0%) => DCM (90%)/MeOH (10%) for 30 min, followed by MeOH (100%) for 5 min. Compound **10** was obtained as a pale-yellow amorphous substance (330 mg, 88% yield).

^1^HNMR (400 MHz, DMSO-*d6*, δ): 8.35–8.25 (br.d., 1H, F5NH*mn*), 8.22–8.13 (m, 1H, F6NH*mn*), 7.93 (t, J = 5.4 Hz, X3NHk*m*), 7.91–7.84 (m, X3NHk*n* + FmocHt), 7.67 (d, J = 7.5 Hz, 2H, FmocHd), 7.60–7.52 (m, 1H, X7NH), 7.45–7.35 (m, FmocHk + X8Hd*n*), 7.35–7.08 (m, X8He*n* + FmocHe + X8Hd*m* + X8He*m* + X7NHd + F6He + F6Hd + X8Ht*mn* + F5He + F6Hk + F5Hk + F5Hd + X8Hg*mn*), 6.36–6.21 (m, 2H, K2NH + E1NH, *m* + *n*), 4.54 (s, *n*) and 4.47 (s, *m*) (2H, X8Ha, *m* + *n*), 4.44–4.36 (m, 1H, F6Ha), 4.36–4.25 (m, 3H, F5Ha + FmocHa), 4.20 (t, J = 6.9 Hz, 1H, FmocHb), 4.08–3.99 (m, 1H, E1Ha), 3.99–3.90 (m, 1H, K2Ha*mn*), 3.21 (t, J = 7.3 Hz, *n*) and 3.16 (t, J = 7.3 Hz, *m*) (2H, K2He, *m + n*), 3.11–2.84 (m, 9H, F6Hb(a) + X7Hg + X7Ha + X3He(*mn*) + F6Hb(b) + F5Hb(a)), 2.70–2.60 (m, 1H, F5Hb(b)), 2.40–2.10 (m, 8H, X3Ha*m* + X4Hb*mn* + E1Hg + X4Ha*mn* + X3Ha*n*), 1.92–1.80 (m, 1H, E1Hb(a)), 1.72–1.62 (m, 1H, E1Hb(b)), 1.62–1.55 (m, 1H, K2Hb(a)), 1.54–1.10 (m, 13H, X7Hb + X3Hb + K2Hb(b) + X3Hd + K2Hd + K2Hg + X3Hg, *m* + *n*), 1.40–1.34 (m, 27H, tBu). The ^1^H NMR spectrum of compound **10** is presented in Figure S13 (in Supplementary Materials).

^13^C NMR (100 MHz, DMSO-*d_6_*, δ): 172.79 (X4Cg(*n*)), 172.76 (X4Cg(*m*)), 172.24 (K2C(*n*)), 172.20 (K2C(*m*)), 172.15 (X3C(*n*)), 172.13 (X3C(*m*)), 171.92 (E1C), 171.57 (X4C(*mn*)), 171.45 (E1Cd), 171.11 (F5C), 170.67 (F6C) 157.15 (U(*mn*)), 156.12 (C(O)Fmoc), 143.94 (FmocCg), 141.16 (X8Cb(*m*)), 140.77 (X8Cb(*n*) + FmocCte), 138.12 (F6Cg), 138.02 (F5Cg), 133.44 (X8Ck(*n*)), 133.09 (X8Ck(*m*)), 130.59 (X8Cd(*n*)), 130.23 (X8Cd(*m*)), 129.04 (F6Cd + F5Cd), 128.16 (F6Ce), 128.06 (F5Ce), 127.63 (FmocCk), 127.21 (X8Ct(*m*)), 127.15 (X8Ce(*n*)), 127.09 (FmocCt), 126.86 (X8Ce(*m*)), 126.29 (F6Ck + X8Ct(*n*)), 126.24 (F5Ck), 126.05 (X8Cg(*m*)), 125.17 (FmocCe), 124.95 (X8Cg(*n*)), 120.13 (FmocCd), 80.59 (E1tBu), 80.42 (K2tBu(*m*)), 80.33 (K2tBu(*n*)), 79.77 (E1dtBu), 65.32 (FmocCa), 54.94 (F5Ca), 54.40 (F6Ca), 53.01 (K2Ca(*n*)), 52.87 (K2Ca(*m*)), 52.20 (E1Ca), 49.62 (X8Ca(*n*)), 47.11 (X8Ca(*m*)), 46.80 (FmocCb + K2Ce(*m*)), 45.19 (K2Ce(*n*)), 38.65 (X3Ce(*m*)), 38.60 (X3Ce(*n*)), 37.90 (X7Cg), 37.11 (F5Cb), 36.82 (F6Cb), 36.39 (X7Ca), 32.32 (X3Ca(*n*)), 31.95 (X3Ca(*m*)), 31.82 (K2Cb), 30.93 (E1Cg), 30.69 (X4Ca), 30.58 (X4Cb), 29.20 (X7Cb), 29.05 (X3Cd(*m*)), 28.96 (X3Cd(*n*)), 27.74 (tBuE1), 27.65 (tBuK2 + K2Cd(*m*)), 27.62 (tBuE1d + E1Cb), 26.69 (K2Cd(*n*)), 26.31 (X3Cg(*m*)), 26.22 (X3Cg(*n*)), 24.73 (X3Cb(*m*)), 24.60 (X3Cb(*n*)), 22.43 (K2Cg(*n*)) 22.25 (K2Cg(*m*)). The ^13^C NMR spectrum of compound **10** is presented in Figure S14 (in Supplementary Materials).

ESI-MS C_77_H_101_ClN_8_O_14_: *m*/*z* calcd. for [M + H^+^]^+^: 1397.72; found: 1397.65. The ESI-MS spectrum of compound **10** is presented in Figure S15 (in Supplementary Materials).

HRMS (m/z, ESI): calcd. for C_77_H_101_ClN_8_O_14_–[M + H]^+^ 1397.7199; found: 1397.7210. The HRMS of compound **10** is presented in Figure S16 (in Supplementary Materials).

**Compound 11.** Compound 10 (1 eq; 200 mg; 0.143 mmol) was dissolved in an Et_2_NH/DMF (20 eq Et_2_NH, 10 mL DMF) mixture and stirred for 20 min; then, the solvent was removed under reduced pressure and the residue was re-evaporated with DCM three times. The product was precipitated with Et_2_O and washed twice with Et_2_O (2 mL). The residue was purified by reverse-phase column chromatography (Puriflash PF-15C18AQ-F0025 (15 μ 35 g): H_2_O*TFA (0.1%) (80%)/MeCN (20%) => H_2_O*TFA (0.1%) (0%)/MeCN (100%) for 30 min after MeCN (100%) for 5 min. Compound **11** was obtained as a *TFA salt as a white amorphous solid (160 mg, 89% yield).

^1^HNMR (400 MHz, DMSO-*d6*, δ): 8.37 (d, J = 7.3 Hz, 1H, F5NH*mn*), 8.23–8.15 (br.d, 1H, F6NH*mn*), 8.00 (t, J = 5.4 Hz, *m*) and 7.97 (t, J = 5.4 Hz, *n*) (1H, X3NHk, *m* + *n*), 7.82 (br.s, 3H, X7NHd), 7.77–7.69 (m, 1H, X7NH), 7.42–7.09 (m, 14H, X8Hd*n* + X8He*n* + X8Hd*m* + X8He*m* + F6He + F6Hd + X8Ht*mn* + F5He + F6Hk + F5Hk + F5Hd + X8Hg*mn*), 6.39–6.23 (m, 2H, K2NH*m* + K2NH*n* + E1NH*m* + E1NH*n*), 4.55 (s, *n*) and 4.47 (s, *m*) (2H, X8Ha, *m* + *n*), 4.43–4.33 (m, 1H, F6Ha), 4.33–4.23 (m, 1H, F5Ha), 4.08–3.99 (m, 1H, E1Ha), 3.99–3.90 (m, 1H, K2Ha*m* + K2Ha*n*), 3.26–3.11 (m, 3H, K2He*mn* + X7Hg(a)), 3.11–2.85 (m, 6H, F6Hb(a) + X7Hg(b) + X3He(*mn*) + F6Hb(b) + F5Hb(a)), 2.78–2.61 (m, 3H, X7Ha + F5Hb(b)), 2.40–2.10 (m, 8H, X3Ha*m* + X4Hb*mn* + E1Hg + X4Ha*mn* + X3Ha*n*), 1.92–1.80 (m, 1H, E1Hb(a)), 1.73–1.61 (m, 3H, X7Hb + E1Hb(b)), 1.62–1.55 (m, 1H, K2Hb(a)), 1.54–1.10 (m, 11H, X3Hb + K2Hb(b) + X3Hd + K2Hd + K2Hg + X3Hg, *m* + *n*), 1.40–1.34 (m, 27H, tBu). The ^1^H NMR spectrum of compound **11** is presented in Figure S17 (in Supplementary Materials).

^13^C NMR (100 MHz, DMSO-*d_6_*, δ): 172.91 (X4Cg(*n*)), 172.88 (X4Cg(*m*)), 172.24 (K2C(*n*)), 172.20 (K2C(*m*)), 172.15 (X3C(*n*)), 172.13 (X3C(*m*)), 171.92 (E1C), 171.61 (X4C(*mn*)), 171.46 (E1Cd), 171.23 (F5C), 171.09 (F6C), 157.18 (U(*m*)), 157.16 (U(*n*)), 141.18 (X8Cb(*m*)), 140.78 (X8Cb(*n*)), 138.08 (F6Cg), 138.02 (F5Cg), 133.41 (X8Ck(*n*)), 133.07 (X8Ck(*m*)), 130.61 (X8Cd(*n*)), 130.25 (X8Cd(*m*)), 129.05 (F6Cd + F5Cd), 128.20 (F6Ce), 128.07 (F5Ce), 127.20 (X8Ct(*m*)), 127.15 (X8Ce(*n*)), 126.86 (X8Ce(*m*)), 126.34 (F6Ck), 126.31 (X8Ct(*n*)), 126.25 (F5Ck), 126.06 (X8Cg(*m*)), 124.97 (X8Cg(*n*)), 80.55 (E1tBu), 80.38 (K2tBu(*m*)), 80.30 (K2tBu(*n*)), 79.77 (E1dtBu), 55.10 (F5Ca), 54.53 (F6Ca), 53.01 (K2Ca(*n*)), 52.88 (K2Ca(*m*)), 52.19 (E1Ca), 49.62 (X8Ca(*n*)), 47.10 (X8Ca(*m*)), 46.81 (K2Ce(*m*)), 45.22 (K2Ce(*n*)), 38.64 (X3Ce(*m*)), 38.58 (X3Ce(*n*)), 36.92 (F5Cb), 36.79 (F6Cb), 36.58 (X7Cg), 35.81 (X7Ca), 32.33 (X3Ca(*n*)), 31.95 (X3Ca(*m*)), 31.79 (K2Cb), 30.92 (E1Cg), 30.66 (X4Ca), 30.57 (X4Cb), 29.05 (X3Cd(*m*)), 28.97 (X3Cd(*n*)), 27.75 (tBuE1), 27.66 (tBuK2 + K2Cd(*m*)), 27.63 (tBuE1d), 27.57 (E1Cb), 27.09 (X7Cb), 26.70 (K2Cd(*n*)), 26.32 (X3Cg(*m*)), 26.23 (X3Cg(*n*)), 24.74 (X3Cb(*m*)), 24.60 (X3Cb(*n*)), 22.45 (K2Cg(*n*)) 22.26 (K2Cg(*m*)). The ^13^C NMR spectrum of compound **11** is presented in Figure S18 (in Supplementary Materials).

ESI-MS C_62_H_91_ClN_8_O_12_: *m*/*z* calcd. for [M + H^+^]^+^: 1175.65; found: 1175.6. The ESI-MS of compound **11** is presented in Figure S19 (in Supplementary Materials).

HRMS (*m*/*z*, ESI): calcd. for C_62_H_91_ClN_8_O_12_–[M + H]^+^ 1175,6518; found: 1175.6520. The HRMS of compound **11** is presented in Figure S20 (in Supplementary Materials).

**Compound 13.** Compound **12** (1 eq; 1800 mg; 4.376 mmol, calculated assuming that **12** is only a mono-stannylated derivative) was dissolved in 40 mL dry DCM. Then, NHS (1.2 eq; 604 mg; 5.25 mmol), DMAP (0.1 eq; 53 mg; 0.438 mmol), and EDC*HCl (1.1 eq; 924 mg; 4.82 mmol) in DMF (4 mL) were added dropwise. The mixture was stirred overnight. After the reaction proceeded, the solvent was removed using a rotary evaporator, and the residue was dissolved in DCM (100 mL) and transferred to a separating funnel, where it was washed twice with H_2_O and then with saturated NaCl solution. The organic layer was dried over Na_2_SO_4_. The solvent was removed using a rotary evaporator and the residue was purified using column chromatography (Puriflash on a column (40–60 μ 120 g)): P.E. (97%)/E.A. (3%) for 7 min, then P.E. (97%)/E.A. (3%) => P.E. (60%)/E.A. (40%) for 40 min, then P.E. (60%)/E.A. (40%) => P.E. (0%)/E.A. (100%) for 5 min, then E.A. (100%) for 10 min. As a result, a fraction was isolated, which was a pale-yellow transparent oily substance (m = 1729 mg, 78%).

^1^HNMR (400 MHz, DMSO-*d6*, δ): 8.18–8.06 (m., 1H, 2), 8.05–7.97 (m, 1H, 4), 7.96–7.82 (m, 1H, 6), 7.65–7.55 (m, 1H, 5), 2.89 (s, 4H, 9), 1.63–1.39 (m, 6H, 11), 1.35–1.21 (m, 6H, 12), 1.20–1.00 (m, 6H, 10), 0.84 (t, J = 7.3 Hz, 9H, 13). The ^1^H NMR spectrum of compound **13** is presented in Figure S21 (in Supplementary Materials).

^13^C NMR (100 MHz, DMSO-*d_6_*, δ): 170.33 (8), 162.14 (7), 143.46 (6), 143.24 (1), 137.05 (2), 129.64 (3), 128.84 (4), 124.07 (5), 28.52 (11), 26.67 (12), 25.57 (9), 13.48 (10), 9.36 (13). The ^13^C NMR spectrum of compound **13** is presented in Figure S22 (in Supplementary Materials).

ESI-MS C_23_H_35_NO_4_^118^Sn: *m*/*z* calcd. for [M + Na^+^]^+^: 530,15; found: 530.10. The ESI-MS spectrum of compound **13** is presented in Figure S23 (in Supplementary Materials).

HRMS (*m*/*z*, ESI): calcd for C_23_H_35_NO_4_^120^Sn–[M + Na^+^]^+^ 532,1480; found: 532,1487. The HRMS of compound **13** is presented in Figure S24 (in Supplementary Materials).

**Compound 14.** I_2_ (1 eq; 97.5 mg; 0.384 mmol) was dissolved in 0.1N NaOH (2300 μL = V1), and then AcOH (3%) in CHCl_3_ (2300 μL = V2) was added (V1 = V2) followed by tert-butyl hydroperoxide (TBHP) in CHCl_3_ (this solution is prepared in advance by adding 1800 μL of 70% TBHP in water to 11209 μL of CHCl_3_, after which Na_2_SO_4_ is added to the prepared mixture to bind water) (11513 μL). Then, compound **13** (1 eq; 195 mg; 0.383 mmol) in CHCl_3_ (3900 μL) was added. The mixture was stirred for 30 min, and the solvent was then removed under reduced pressure. The product was precipitated with H_2_O and washed twice with H_2_O (2 mL) and twice with P.E. (2 mL). The residue was purified by reverse-phase column chromatography (Puriflash PF-15C18AQ-F0025 (15μ 40g): H_2_O (90%)/MeCN (10%) => H_2_O (0%)/MeCN (100%) for 30 min followed by MeCN (100%) for 15 min. As a result, a fraction was isolated in the form of a white powder (m = 95 mg, 72%), which is the target substance.

^1^H NMR (400 MHz, DMSO-d6, δ): 8.34 (t, J = 1.7 Hz, 1H, 2), 8.21 (ddd, J = 7.9, 1.7, 1.0 Hz, 1H, 6), 8.10 (ddd, J = 7.9, 1.7, 1.0 Hz, 1H, 4), 7.45 (t, J = 7.9 Hz, 1H, 5), 2.90 (s, 4H, 9). The ^1^H NMR spectrum of compound **14** is presented in Figure S25 (in Supplementary Materials).

^13^C NMR (100 MHz, DMSO-*d_6_*, δ): 170.22 (8), 160.60 (7), 144.11 (6), 137.87 (2), 131.62 (3), 129.30 (5), 126.41 (4), 95.53 (1), 25.57 (9). The ^13^C NMR spectrum of compound **14** is presented in Figure S26 (in Supplementary Materials).

**Compound 16.** Compound **15** (1 eq; 1380 mg; 3.35 mmol, calculated assuming that 15 is only a mono-stannylated derivative) was dissolved in 15 mL dry THF. Then, NHS (1.2 eq; 463 mg; 4.02 mmol) and DCC (1 eq; 691 mg; 3.35 mmol) in THF (15 mL) were added dropwise. The mixture was stirred overnight. The precipitated dicyclohexylurea was removed by filtration through a fritted funnel. The precipitate was washed with 2 × 6 mL of THF, and then the solvent was removed using a rotary evaporator. The residue was purified using column chromatography (Puriflash on a column (40–60 μ 120 g)): P.E. (97%)/E.A. (3%) for 7 min, then P.E. (97%)/E.A. (3%) => P.E. (60%)/E.A. (40%) for 40 min, then P.E. (60%)/E.A. (40%) => P.E. (0%)/E.A. (100%) for 5 min, then E.A. (100%) for 10 min. As a result, a fraction was isolated, which is a pale-yellow transparent oily substance (m = 772 mg, 45%).

^1^HNMR (400 MHz, DMSO-*d6*, δ): 8.05–7.93 (m, 1H, 3), 7.82–7.66 (m, 1H, 4), 2.89 (s, 4H, 7), 1.65–1.39 (m, 6H, 9), 1.36–1.21 (m, 6H, 10), 1.20–1.00 (m, 6H, 8), 0.84 (t, J = 7.3 Hz, 9H, 11). The ^1^H NMR spectrum of compound **16** is presented in Figure S27 (in Supplementary Materials).

^13^C NMR (100 MHz, DMSO-*d_6_*, δ): 170.33 (6), 162.10 (5), 153.06 (1), 137.16 (2), 128.53 (3), 124.01 (4), 28.52 (9), 26.67 (10), 25.56 (7), 13.51 (8), 9.37 (11). The ^13^C NMR spectrum of compound **16** is presented in Figure S28 (in Supplementary Materials).

HRMS (*m*/*z*, ESI): calcd for C_23_H_35_NO_4_^120^Sn–[M + Na^+^]^+^ 532,1480; found: 532,1485. The HRMS of compound **16** is presented in Figure S29 (in Supplementary Materials).

**Compound 17.** I_2_ (1 eq; 75 mg; 0.295 mmol) was dissolved in 0.1N NaOH (1770 μL = V1), and then AcOH (3%) in CHCl_3_ (1770 μL = V2) was added (V1 = V2) followed by tert-butyl hydroperoxide (TBHP) in CHCl_3_ (this solution is prepared in advance by adding 1264 μL of 70% TBHP in water to 7872 μL of CHCl_3_, after which Na_2_SO_4_ is added to the prepared mixture to bind water) (8856 μL). Then, compound **16** (1 eq; 150 mg; 0.295 mmol) in CHCl_3_ (3000 μL) was added. The mixture was stirred for 30 min, and the solvent was removed under reduced pressure. The product was precipitated with H_2_O and washed twice with H_2_O (2 mL) and twice with P.E./E.A. (80/20) (2 mL). As a result, a fraction was isolated in the form of a white powder (m = 98.5 mg, 97%), which is the target substance.

^1^HNMR (400 MHz, DMSO-*d6*, δ): 8.06 (d, J = 8.5 Hz, 2H, 3), 7.83 (d, J = 8.5 Hz, 1H, 2), 2.89 (s, 4H, 7). The ^1^H NMR spectrum of compound **17** is presented in Figure S30 (in Supplementary Materials).

^13^C NMR (100 MHz, DMSO-*d_6_*, δ): 170.28 (6), 161.69 (5), 138.65 (2), 131.39 (3), 123.88 (4), 105.01 (1), 25.58 (7). The ^13^C NMR spectrum of compound **17** is presented in Figure S31 (in Supplementary Materials).

**Compound 18.** Compound **11** (1 eq.; 228.5 mg; 177.1 mmol) was dissolved in a mixture of DCM/TFA/TIPS/H_2_O (46.25%/46.25%/2.5%/5%; *v*/*v* respectively, 8 mL). The mixture was stirred for 3 h. Then, the solvent was removed under reduced pressure, and the residue was re-evaporated with DCM three times. The product was precipitated with Et_2_O and washed twice with Et_2_O (1 mL). After that, the compound was purified by column chromatography (Puriflash on the column PF-15C18HP-F0012 (15 μ 20 g), eluent: H_2_O (90%)/MeCN (10%) => H_2_O (0%)/MeCN (100%) for 30 min, then MeCN (100%) for 5 min. The individual compound **18** was obtained as a white amorphous solid (159 mg, yield 80%).

^1^HNMR (400 MHz, DMSO-*d6*, δ): 8.36 (d, J = 7.3 Hz, 1H, F5NH*mn*), 8.24–8.16 (m, 1H, F6NH*m* + F6NH*n*), 7.96 (t, J = 5.4 Hz, *m*) and 7.93 (t, J = 5.4 Hz, *n*) (1H, X3NHk, *m* + *n*), 7.75–7.58 (m, 4H, X7NH + X7NH_3_^+^d), 7.42–7.09 (m, 14H, X8Hd*n* + X8He*n* + X8Hd*m* + X8He*m* + F6He + F6Hd + X8Ht*mn* + F5He + F6Hk + F5Hk + F5Hd + X8Hg*mn*), 6.39–6.25 (m, 2H, K2NH*m* + K2NH*n* + E1NH*m* + E1NH*n*), 4.55 (s, *n*) and 4.47 (s, *m*) (2H, X8Ha, *m* + *n*), 4.42–4.33 (m, 1H, F6Ha), 4.33–4.23 (m, 1H, F5Ha), 4.14–3.98 (m, 2H, E1Ha + K2Ha*m* + K2Ha*n*), 3.25–3.10 (m, 3H, K2He*mn* + X7Hg(a)), 3.10–2.85 (m, 6H, F6Hb(a) + X7Hg(b) + X3He(*mn*) + F6Hb(b) + F5Hb(a)), 2.78–2.58 (m, 3H, X7Ha + F5Hb(b)), 2.40–2.10 (m, 8H, X3Ha*m* + X4Hb*mn* + E1Hg + X4Ha*mn* + X3Ha*n*), 1.97–1.85 (m, 1H, E1Hb(a)), 1.76–1.56 (m, 4H, E1Hb(b) + X7Hb + K2Hb(a)), 1.56–1.11 (m, 11H, X3Hb + K2Hb(b) + X3Hd + K2Hd + K2Hg + X3Hg, *m* + *n*). The ^1^H NMR spectrum of compound **18** is presented in Figure S32 (in Supplementary Materials).

^13^C NMR (100 MHz, DMSO-*d_6_*, δ): 174.61 (K2C(*n*)), 174.57 (K2C(*m*)), 174.27 (E1C(*mn*)), 173.84 (E1Cd), 173.06 (X4Cg(*n*)), 173.03 (X4Cg(*m*)), 172.23 (X3C), 171.70 (X4C(*mn*)), 171.33 (F5C), 171.24 (F6C), 159.03 (C(O)TFA), 158.66 (C(O)TFA), 158.30 (C(O)TFA), 157.92 (C(O)TFA), 157.36 (U), 141.27 (X8Cb(*m*)), 140.87 (X8Cb(*n*)), 138.09 (F6Cg), 138.03 (F5Cg), 133.46 (X8Ck(*n*)), 133.11 (X8Ck(*m*)), 130.66 (X8Cd(*n*)), 130.30 (X8Cd(*m*)), 129.07 (F6Cd + F5Cd), 128.28 (F6Ce), 128.16 (F5Ce), 127.24 (X8Ct(*m*)), 127.19 (X8Ce(*n*)), 126.90 (X8Ce(*m*)), 126.44 (F6Ck), 126.34 (X8Ct(*n*)) + F5Ck), 126.14 (X8Cg(*m*)), 125.02 (X8Cg(*n*)), 117.00 (CF_3_), 114.17 (CF_3_), 55.15 (F5Ca), 54.58 (F6Ca), 52.33 (K2Ca(*n*)), 52.20 (K2Ca(*m*)), 51.72 (E1Ca), 49.67 (X8Ca(*n*)), 47.21 (X8Ca(*m*)), 46.93 (K2Ce(*m*)), 45.39 (K2Ce(*n*)), 38.72 (X3Ce(*m*)), 38.65 (X3Ce(*n*)), 36.94 (F5Cb), 36.82 (F6Cb), 36.72 (X7Cg), 35.79 (X7Ca), 32.34 (X3Ca(*n*)), 31.93 (X3Ca(*m*)), 31.85 (K2Cb), 30.72 (X4Ca), 30.59 (X4Cb), 29.97 (E1Cg), 29.11 (X3Cd(*m*)), 29.01 (X3Cd(*n*)), 27.85 (K2Cd(*m*)), 27.59 (E1Cb), 27.23 (X7Cb), 26.80 (K2Cd(*n*)), 26.34 (X3Cg(*m*)), 26.26 (X3Cg(*n*)), 24.77 (X3Cb(*m*)), 24.63 (X3Cb(*n*)), 22.56 (K2Cg(*n*)) 22.40 (K2Cg(*m*)). The ^13^C NMR spectrum of compound **18** is presented in Figure S33 (in Supplementary Materials).

ESI-MS C_50_H_67_ClN_8_O_12_: *m*/*z* calcd. for [M + H^+^]^+^: 1007.46; found: 1007.55. The ESI-MS spectrum of compound **18** is presented in Figure S34 (in Supplementary Materials).

HRMS (*m*/*z*, ESI): calcd. for C_50_H_67_ClN_8_O_12_–[M + H]^+^ 1007.4640; found: 1007.4622. The HRMS of compound **18** is presented in Figure S35 (in Supplementary Materials).

**Compound 19.** Compound **18** (1 eq.; 30 mg; 26.75 μmol) and DIPEA (6 eq.; 28 μL; 160.5 μmol) were dissolved in DMF (2 mL). Compound **13** (1 eq.; 14 mg; 26.75 μmol) was added to the obtained mixture, and the system was purged with argon. The mixture was stirred for 6 h, and the solvent was evaporated under reduced pressure. The residue was then purified by column chromatography (Puriflash on a column of PF-15C18HP-F0012 (15 μ 20 g), eluent: H_2_O (90%)/MeCN (10%) => H_2_O (0%)/MeCN (100%) for 20 min, then MeCN (100%) for 5 min. Compound **19** was obtained as a white powder (28 mg, yield 75%).

^1^HNMR (400 MHz, DMSO-*d6*, δ): 13.30–11.30 (br.s, 3H, COOH), 8.45–8.36 (m, 1H, X7NHδ), 8.33–8.25 (m, 1H, F5NH*mn*), 8.24–8.14 (m, 1H, F6NH*m* + F6NH*n*), 7.97–7.83 (m, 2H, X3NHζ*mn* + 2), 7.78–7.70 (m, 1H, 6), 7.69–7.59 (m, 1H, X7NH), 7.59–7.50 (m, 1H, 4), 7.45–7.08 (m, 15H, 5 + X8Hδ*n* + X8Hε*n* + X8Hδ*m* + X8Hε*m* + F6Hε + F6Hδ + X8Hη*mn* + F5Hε + F6Hζ + F5Hζ + F5Hδ + X8Hγ*mn*), 6.38–6.24 (m, 2H, K2NH*m* + K2NH*n* + E1NH*m* + E1NH*n*), 4.54 (s, *n*) and 4.47 (s, *m*) (2H, X8Hα, *m* + *n*), 4.44–4.37 (m, 1H, F6Hα), 4.37–4.27 (m, 1H, F5Hα), 4.14–3.98 (m, 2H, E1Hα + K2Hα*m* + K2Hα*n*), 3.26–2.84 (m, 11H, K2Hε*mn* + X7Hγ(a)) + F6Hβ(a) + X7Hγ(b) + X3Hε(*mn*) + F6Hβ(b) + X7Hα + F5Hβ(a)), 2.70–2.60 (m, 1H, F5Hβ(b)), 2.40–2.10 (m, 8H, X3Hα*m* + X4Hβ*mn* + E1Hγ + X4Hα*mn* + X3Hα*n*), 1.97–1.85 (m, 1H, E1Hβ(a)), 1.76–1.67 (m, 1H, E1Hβ(b)), 1.67–1.13 (m, 26H, X7Hβ + K2Hβ(a) + 8 + X3Hβ + K2Hβ(b + X3Hδ + 9 + K2Hδ + K2Hγ + X3Hγ, *m* + *n*), 1.13–0.95 (m, 6H, 10), 0.83 (t, J = 7.1 Hz, 9H, 11). The ^1^H NMR spectrum of compound **19** is presented in Figure S36 (in Supplementary Materials).

^13^C NMR (100 MHz, DMSO-*d_6_*, δ): 174.54 (K2C(*n*)), 174.51 (K2C(*m*)), 174.22 (E1C(*mn*)), 173.80 (E1Cδ), 172.74 (X4Cγ(*n*)), 172.68 (X4Cγ(*m*)), 172.12 (X3C), 171.50 (X4C(*mn*)), 171.12 (F5C), 170.78 (F6C), 166.60 (7), 157.26 (U), 141.50 (6), 141.22 (X8Cβ(*m*)), 140.80 (X8Cβ(*n*)), 138.90 (1), 138.10 (F6Cγ), 138.00 (F5Cγ), 135.53 (2), 134.73 (3), 133.92 (5), 133.40 (X8Cζ(*n*)), 133.04 (X8Cζ(*m*)), 130.59 (X8Cδ(*n*)), 130.23 (X8Cδ(*m*)), 129.04 (F6Cδ + F5Cδ), 128.16 (F6Cε), 128.04 (F5Cε), 127.75 (4), 127.20 (X8Cη(*m*)), 127.12 (X8Cε(*n*)), 126.83 (X8Cε(*m*)), 126.29 (F6Cζ + X8Cη(*n*)), 126.22 (F5Cζ), 126.08 (X8Cγ(*m*)), 124.95 (X8Cγ(*n*)), 54.86 (F5Cα), 54.41 (F6Cα), 52.25 (K2Cα(*n*)), 52.14 (K2Cα(*m*)), 51.68 (E1Cα), 49.61 (X8Cα(*n*)), 47.13 (X8Cα(*m*)), 46.85 (K2Cε(*m*)), 45.31 (K2Cε(*n*)), 38.64 (X3Cε(*m*)), 38.57 (X3Cε(*n*)), 37.09 (F5Cβ), 36.86 (F6Cβ), 36.69 (X7Cγ), 35.48 (X7Cα), 32.28 (X3Cα(*n*)), 31.86 (X3Cα(*m*)), 31.81 (K2Cβ), 30.67 (X4Cα), 30.56 (X4Cβ), 29.97 (E1Cγ), 29.07 (X3Cδ(*m*) + X7Cβ), 28.95 (X3Cδ(*n*)), 28.60 (9), 27.80 (K2Cδ(*m*)), 27.60 (E1Cβ), 26.70 (K2Cδ(*n*) + 10), 26.28 (X3Cγ(*m*)), 26.21 (X3Cγ(*n*)), 24.72 (X3Cβ(*m*)), 24.57 (X3Cβ(*n*)), 22.53 (K2Cγ(*n*)) 22.34 (K2Cγ(*m*)), 13.58 (8), 9.20 (11). The ^13^C NMR spectrum of compound **19** is presented in Figure S37 (in Supplementary Materials).

HRMS (m/z, ESI): calcd. for C_69_H_97_ClN_8_O_13_Sn–[M + H]^+^ 1401,5958; found: 1401,5990. The HRMS of compound **19** is presented in Figure S38 (in Supplementary Materials).

**Compound 20.** Compound **18** (1 eq.; 38 mg; 34 μmol) and DIPEA (6 eq.; 30 μL; 174.36 μmol) were dissolved in DMF (2 mL). Compound **16** (1 eq.; 17.3 mg; 34 μmol) was added to the obtained mixture, the system was purged with argon, and the mixture was stirred for 6 h. The solvent was evaporated under reduced pressure, and the residue was then purified by column chromatography (Puriflash on a column of PF-15C18HP-F0012 (15 μ 20 g), eluent: H_2_O (90%)/MeCN (10%) => H_2_O (0%)/MeCN (100%) for 20 min, then MeCN (100%) for 5 min. Compound **20** was obtained as a white powder (30.5 mg, yield 64%).

^1^HNMR (400 MHz, DMSO-*d6*, δ): 12.95–11.50 (br.s, 3H, COOH), 8.44–8.34 (m, 1H, X7NHδ), 8.34–8.26 (m, 1H, F5NH*mn*), 8.25–8.15 (m, 1H, F6NH*m* + F6NH*n*), 7.92 (t, J = 5.4 Hz, *m*) and 7.89 (t, J = 5.4 Hz, *n*) (1H, X3NHζ, *m* + *n*), 7.75 (d, J = 7.5 Hz, 2H, 3), 7.70–7.60 (m, 1H, X7NH), 7.51 (d, J = 7.5 Hz, 2H, 2), 7.41–7.07 (m, 14H, X8Hδ*n* + X8Hε*n* + X8Hδ*m* + X8Hε*m* + F6Hε + F6Hδ + X8Hη*mn +* F5Hε + F6Hζ + F5Hζ + F5Hδ + X8Hγ*mn*), 6.39–6.22 (m, 2H, K2NH*m* + K2NH*n* + E1NH*m* + E1NH*n*), 4.54 (s, *n*) and 4.46 (s, *m*) (2H, X8Hα, *m* + *n*), 4.44–4.37 (m, 1H, F6Hα), 4.37–4.27 (m, 1H, F5Hα), 4.14–3.98 (m, 2H, E1Hα + K2Hα*m* + K2Hα*n*), 3.25–2.84 (m, 11H, K2Hε*mn* + X7Hγ(a + F6Hβ(a) + X7Hγ(b) + X3Hε(*mn*) + F6Hβ(b) + X7Hα + F5Hβ(a)), 2.70–2.60 (m, 1H, F5Hβ(b)), 2.40–2.10 (m, 8H, X3Hα*m* + X4Hβ*mn* + E1Hγ + X4Hα*mn* + X3Hα*n*), 1.96–1.84 (m, 1H, E1Hβ(a)), 1.77–1.67 (m, 1H, E1Hβ(b)), 1.67–1.13 (m, 26H, X7Hβ + K2Hβ(a) + 6 + X3Hβ + K2Hβ(b) + X3Hδ + 7 + K2Hδ + K2Hγ + X3Hγ, *m* + *n*), 1.13–0.95 (m, 6H, 8), 0.83 (t, J = 7.1 Hz, 9H, 9). The ^1^H NMR spectrum of compound **20** is presented in Figure S39 (in Supplementary Materials).

^13^C NMP (100 MHz, DMSO-*d_6_*, δ): 174.56 (K2C(*mn*)), 174.26 (E1C(*mn*)), 173.85 (E1Cδ), 172.77 (X4Cγ(*n*)), 172.72 (X4Cγ(*m*)), 172.16 (X3C), 171.53 (X4C(*mn*)), 171.18 (F5C), 170.81 (F6C), 166.50 (5), 157.29 (U), 145.70 (4), 141.23 (X8Cβ(*m*)), 140.83 (X8Cβ(*n*)), 138.12 (F6Cγ), 138.02 (F5Cγ), 136.10 (3), 134.29 (1), 133.42 (X8Cζ(*n*)), 133.06 (X8Cζ(*m*)), 130.60 (X8Cδ(*n*)), 130.25 (X8Cδ(*m*)), 129.06 (F6Cδ + F5Cδ), 128.19 (F6Cε), 128.06 (F5Cε), 127.21 (X8Cη(*m*)), 127.13 (X8Cε(*n*)), 126.84 (X8Cε(*m*)), 126.36 (2), 126.31 (F6Cζ + X8Cη(*n*)), 126.23 (F5Cζ), 126.10 (X8Cγ(*m*)), 124.97 (X8Cγ(*n*)), 54.89 (F5Cα), 54.47 (F6Cα), 52.29 (K2Cα(*n*)), 52.20 (K2Cα(*m*)), 51.76 (E1Cα), 49.65 (X8Cα(*n*)), 47.16 (X8Cα(*m*)), 46.88 (K2Cε(*m*)), 45.33 (K2Cε(*n*)), 38.65 (X3Cε(*m*)), 38.59 (X3Cε(*n*)), 37.09 (F5Cβ), 36.87 (F6Cβ), 36.71 (X7Cγ), 35.48 (X7Cα), 32.30 (X3Cα(*n*)), 31.89 (X3Cα(*m*)), 31.83 (K2Cβ), 30.69 (X4Cα), 30.58 (X4Cβ), 30.08 (E1Cγ), 29.07 (X3Cδ(*m*)), 29.01 (X7Cβ), 28.97 (X3Cδ(*n*)), 28.62 (7), 27.82 (K2Cδ(*m*)), 27.73 (E1Cβ), 26.71 (K2Cδ(*n*) + 8), 26.29 (X3Cγ(*m*)), 26.22 (X3Cγ(*n*)), 24.74 (X3Cβ(*m*)), 24.59 (X3Cβ(*n*)), 22.55 (K2Cγ(*n*)) 22.36 (K2Cγ(*m*)), 13.59 (6), 9.23 (9). The ^13^C NMR spectrum of compound **20** is presented in Figure S40 (in Supplementary Materials).

HRMS (m/z, ESI): calcd. for C_69_H_97_ClN_8_O_13_Sn–[M + H]^+^ 1401; 5958; found: 1401, 5978. The HRMS of compound **20** is presented in Figure S41 (in Supplementary Materials).

**Compound 21. Way A.** Compound **18** (1 eq.; 32.5 mg; 29 μmol) and DIPEA (6 eq.; 30 μL; 174.36 μmol) were dissolved in DMF (4 mL). Compound **14** (1 eq.; 10 mg; 29 μmol) was added to the obtained mixture and the system was purged with argon. The mixture was stirred for 6 h. The solvent was evaporated under reduced pressure and the residue was then purified by column chromatography (Puriflash on a column of PF-15C18HP-F0012 (15 μ 20 g), eluent: H_2_O (90%)/MeCN (10%) => H_2_O (0%)/MeCN (100%) for 20 min, then MeCN (100%) for 5 min. Compound **21** was obtained as a white powder (28.6 mg, yield 80%).

**Way B.** I_2_ (1 eq; 3.4 mg; 13.136 μmol) was dissolved in 0.1N NaOH (79 μL = V1), and then AcOH (3%) in CHCl_3_ (79 μL = V2) was added (V1 = V2), followed by tert-butyl hydroperoxide (TBHP) in CHCl_3_ (this solution is prepared in advance by adding 1800 μL of 70% TBHP in water to 11209 μL of CHCl_3_, after which Na_2_SO_4_ is added to the prepared mixture to bind water) (394 μL). Then, compound **19** (1 eq; 18.4 mg; 13.136 μmol L) in DMF (131 μL) was added. The mixture was stirred for 30 min. Next, the solvent was removed under reduced pressure and the residue was purified by reverse-phase column chromatography (Puriflash PF-15C18AQ-F0012 (15 μ 20 g), eluent: H_2_O (90%)/MeCN (10%) => H_2_O (0%)/MeCN (100%) for 30 min, then MeCN (100%) for 5 min. Compound **21** was obtained as a white powder (10.5 mg, yield 65%).

^1^HNMR (400 MHz, DMSO-*d6*, δ): 12.74–11.94 (br.s, 3H, COOH), 8.55–8.47 (m, 1H, X7NHδ), 8.29 (d, J = 7.4 Hz, 1H, F5NH), 8.22–8.13 (m, 1H, F6NH*mn* + 2), 7.93–7.80 (m, 3H, X3NHζ*mn* + 4 + 6), 7.67–7.58 (m, 1H, X7NH), 7.42–7.08 (m, 15H, X8Hδ*n* + 5 + X8Hε*n* + X8Hδ*m* + X8Hε*m* + F6Hε + F6Hδ + X8Hη*mn* + F5Hε + F6Hζ + F5Hζ + F5Hδ + X8Hγ*mn*), 6.37–6.23 (m, 2H, K2NH*m* + K2NH*n* + E1NH*m* + E1NH*n*), 4.55 (s, *n*) and 4.47 (s, *m*) (2H, X8Hα, *m* + *n*), 4.44–4.37 (m, 1H, F6Hα), 4.37–4.28 (m, 1H, F5Hα), 4.14–3.98 (m, 2H, E1Hα + K2Hα*m* + K2Hα*n*), 3.26–2.84 (m, 11H, K2Hε*mn* + X7Hγ(a)) + F6Hβ(a) + X7Hγ(b) + X3Hε(*mn*) + F6Hβ(b) + X7Hα + F5Hβ(a)), 2.70–2.60 (m, 1H, F5Hβ(b)), 2.40–2.10 (m, 8H, X3Hα*m* + X4Hβ*mn* + E1Hγ + X4Hα*mn* + X3Hα*n*), 1.97–1.85 (m, 1H, E1Hβ(a)), 1.76–1.67 (m, 1H, E1Hβ(b)), 1.67–1.11 (m, 14H, X7Hβ + K2Hβ(a) + X3Hβ + K2Hβ(b) + X3Hδ + K2Hδ + K2Hγ + X3Hγ, *m* + *n*). The ^1^H NMR spectrum of compound **21** is presented in Figure S42 (in Supplementary Materials).

^13^C NMR (100 MHz, DMSO-*d_6_*, δ): 174.56 (K2C(*n*)), 174.53 (K2C(*m*)), 174.25 (E1C(*mn*)), 173.81 (E1Cδ), 172.82 (X4Cγ(*n*)), 172.77 (X4Cγ(*m*)), 172.16 (X3C), 171.55 (X4C(*mn*)), 171.16 (F5C), 170.75 (F6C), 164.71 (7), 157.30 (U), 141.24 (X8Cβ(*m*)), 140.82 (X8Cβ(*n*)), 139.68 (6), 138.13 (F6Cγ), 138.02 (F5Cγ), 136.59 (3), 135.63 (2), 133.42 (X8Cζ(*n*)), 133.07 (X8Cζ(*m*)), 130.61 (X8Cδ(*n*)), 130.54 (5), 130.25 (X8Cδ(*m*)), 129.06 (F6Cδ + F5Cδ), 128.19 (F6Cε), 128.08 (F5Cε), 127.21 (X8Cη(*m*)), 127.14 (X8Cε(*n*)), 126.85 (X8Cε(*m*)), 126.64 (4), 126.31 (F6Cζ + X8Cη(n)), 126.25 (F5Cζ), 126.09 (X8Cγ(*m*)), 124.97 (X8Cγ(*n*)), 94.74 (1), 54.96 (F5Cα), 54.45 (F6Cα), 52.29 (K2Cα(*n*)), 52.16 (K2Cα(*m*)), 51.68 (E1Cα), 49.65 (X8Cα(*n*)), 47.17 (X8Cα(*m*)), 46.89 (K2Cε(*m*)), 45.33 (K2Cε(*n*)), 38.67 (X3Cε(*m*)), 38.60 (X3Cε(*n*)), 37.09 (F5Cβ), 36.97 (X7Cγ), 36.86 (F6Cβ), 36.55 (X7Cα), 32.28 (X3Cα(*n*)), 31.86 (X3Cα(*m*)), 31.81 (K2Cβ), 30.67 (X4Cα), 30.56 (X4Cβ), 29.97 (E1Cγ), 29.07 (X3Cδ(*m*)), 28.95 (X3Cδ(*n*)), 28.87 (X7Cβ), 27.80 (K2Cδ(*m*)), 27.60 (E1Cβ), 26.70 (K2Cδ(*n*), 26.28 (X3Cγ(*m*)), 26.21 (X3Cγ(*n*)), 24.72 (X3Cβ(*m*)), 24.57 (X3Cβ(*n*)), 22.53 (K2Cγ(*n*)) 22.34 (K2Cγ(*m*)). The ^13^C NMR spectrum of compound **21** is presented in Figure S43 (in Supplementary Materials).

ESI-MS C_57_H_70_ClIN_8_O_13_: m/z calcd. for [M + H^+^]^+^: 1237.38; found: 1237.55. The ESI-MS of compound **21** are presented in Figure S44 (in Supplementary Materials).

HRMS (*m*/*z*, ESI): calcd. for C_57_H_70_ClIN_8_O_13_–[M + H]^+^ 1237.3868; found: 1237.3873. The HRMS of compound **21** is presented in Figure S45 (in Supplementary Materials).

**Compound 22. Way A.** Compound **18** (1 eq.; 20 mg; 18 μmol) and DIPEA (6 eq.; 19 μL; 107 μmol) were dissolved in DMF (4 mL). Compound **17** (1 eq.; 6.1 mg; 18 μmol) was added to the obtained mixture and the system was purged with argon. The mixture was stirred for 6 h, and the solvent was then evaporated under reduced pressure. The residue was purified by column chromatography (Puriflash on a column of PF-15C18HP-F0012 (15 μ 20 g), eluent: H_2_O (90%)/MeCN (10%) => H_2_O (0%)/MeCN (100%) for 20 min, then MeCN (100%) for 5 min. Compound **22** was obtained as a white powder (13 mg, yield 59%).

**Way B.** I_2_ (1 eq; 5.4 mg; 21.42 μmol) was dissolved in 0.1N NaOH (129 μL = V1), and AcOH (3%) in CHCl_3_ (129 μL = V2) was added (V1 = V2) followed by tert-butyl hydroperoxide (TBHP) in CHCl_3_ (this solution is prepared in advance by adding 1800 μL of 70% TBHP in water to 11209 μL of CHCl_3_, after which Na_2_SO_4_ is added to the prepared mixture to bind water) (643 μL). Then, compound **20** (1 eq; 30 mg; 21.42 μmol L) in DMF (214 μL) was added. The mixture was stirred for 30 min, and the solvent was then removed under reduced pressure. The residue was purified by reverse-phase column chromatography (Puriflash PF-15C18AQ-F0012 (15 μ 20 g), eluent: H_2_O (90%)/MeCN (10%) => H_2_O (0%)/MeCN (100%) for 30 min, then MeCN (100%) for 5 min. Compound **22** was obtained as a white powder (11.7 mg, yield 44%).

^1^HNMR (400 MHz, DMSO-*d6*, δ): 12.74–11.94 (br.s, 3H, COOH), 8.54–8.45 (m, 1H, X7NHδ), 8.38–8.28 (m, 1H, F5NH), 8.26–8.15 (m, 1H, F6NH*mn* + 2), 7.93 (t, J = 5.4 Hz, *m*) and 7.90 (t, J = 5.4 Hz, *n*) (1H, X3NHζ, *m* + *n*), 7.86–7.81 (m, 2H, 3), 7.68–7.58 (m, 3H, X7NH + 2), 7.41–7.09 (m, 14H, X8Hδ*n* + X8Hε*n* + X8Hδ*m* + X8Hε*m* + F6Hε + F6Hδ + X8Hη*mn* + F5Hε + F6Hζ + F5Hζ + F5Hδ + X8Hγ*mn*), 6.37–6.23 (m, 2H, K2NH*m* + K2NH*n* + E1NH*m* + E1NH*n*), 4.54 (s, *n*) and 4.47 (s, *m*) (2H, X8Hα, *m* + *n*), 4.44–4.35 (m, 1H, F6Hα), 4.35–4.27 (m, 1H, F5Hα), 4.14–3.98 (m, 2H, E1Hα + K2Hα*m* + K2Hα*n*), 3.26–2.84 (m, 11H, K2Hε*mn* + X7Hγ(a)) + F6Hβ(a) + X7Hγ(b) + X3Hε(*mn*) + F6Hβ(b) + X7Hα + F5Hβ(a)), 2.70–2.60 (m, 1H, F5Hβ(b)), 2.40–2.10 (m, 8H, X3Hα*m* + X4Hβ*mn* + E1Hγ + X4Hα*mn* + X3Hα*n*), 1.97–1.85 (m, 1H, E1Hβ(a)), 1.76–1.11 (m, 15H, E1Hβ(b) + X7Hβ + K2Hβ(a) + X3Hβ + K2Hβ(b) + X3Hδ + K2Hδ + K2Hγ + X3Hγ, *m* + *n*). The ^1^H NMR spectrum of compound **22** is presented in Figure S46 (in Supplementary Materials).

^13^C NMR (100 MHz, DMSO-*d_6_*, δ): 174.57 (K2C(*n*)), 174.54 (K2C(*m*)), 174.25 (E1C(*mn*)), 173.82 (E1Cδ), 172.80 (X4Cγ(*n*)), 172.75 (X4Cγ(*m*)), 172.16 (X3C), 171.53 (X4C(*mn*)), 171.17 (F5C), 170.76 (F6C), 165.51 (5), 157.28 (U), 141.23 (X8Cβ(*m*)), 140.83 (X8Cβ(*n*)), 139.68 (6), 138.11 (F6Cγ), 138.01 (F5Cγ), 137.19 (3), 133.97 (2), 133.42 (X8Cζ(*n*)), 133.07 (X8Cζ(*m*)), 130.61 (X8Cδ(*n*)), 130.25 (X8Cδ(*m*)), 129.06 (F6Cδ + F5Cδ), 128.19 (F6Cε), 128.08 (F5Cε), 127.21 (X8Cη(*m*)), 127.14 (X8Cε(*n*)), 126.85 (X8Cε(*m*)), 126.31 (F6Cζ + X8Cη(*n*)), 126.25 (F5Cζ), 126.09 (X8Cγ(*m*)), 124.97 (X8Cγ(*n*)), 98.73 (1), 54.96 (F5Cα), 54.45 (F6Cα), 52.29 (K2Cα(*n*)), 52.16 (K2Cα(*m*)), 51.68 (E1Cα), 49.65 (X8Cα(*n*)), 47.17 (X8Cα(*m*)), 46.89 (K2Cε(*m*)), 45.33 (K2Cε(*n*)), 38.67 (X3Cε(*m*)), 38.60 (X3Cε(*n*)), 37.09 (F5Cβ), 36.88 (X7Cγ + F6Cβ), 36.55 (X7Cα), 32.28 (X3Cα(*n*)), 31.86 (X3Cα(*m*)), 31.81 (K2Cβ), 30.67 (X4Cα), 30.56 (X4Cβ), 30.01 (E1Cγ), 29.07 (X3Cδ(*m*)), 28.95 (X3Cδ(*n*)), 28.87 (X7Cβ), 27.80 (K2Cδ(*m*)), 27.60 (E1Cβ), 26.70 (K2Cδ(*n*), 26.28 (X3Cγ(*m*)), 26.21 (X3Cγ(*n*)), 24.72 (X3Cβ(*m*)), 24.57 (X3Cβ(*n*)), 22.53 (K2Cγ(*n*)) 22.34 (K2Cγ(*m*)). The ^13^C NMR spectrum of compound **22** is presented in Figure S47 (in Supplementary Materials).

ESI-MS C_57_H_70_ClIN_8_O_13_: *m*/*z* calcd. for [M + H^+^]^+^: 1237.38; found: 1237.55. The ESI-MS of compound **22** is presented in Figure S48 (in Supplementary Materials).

HRMS (*m*/*z*, ESI): calcd. for C_57_H_70_ClIN_8_O_13_–[M + H]^+^ 1237.3868; found: 1237.3890. The HRMS of compound **22** is presented in Figure S49 (in Supplementary Materials).

### 4.2. Iodine-123 Production

The ^123^I was obtained from Tosmk Polytechnic University R-7M cyclotron facility. ^123^I was produced by irradiating a [^122^Te]TeO_2_ target by 13.6 MeV deuterons with a current of 20 μA. For the target, 99.6% of enriched-[^122^Te]TeO_2_ and 1% of Al_2_O_3_ were deposited on a platinum backing plate. The irradiation was carried out for 3 h. After irradiation, the ^123^I was isolated from the target by dry distillation methods. The target was heated, then ^123^I was distilled out of the matrix and carried by the sweep gas to a receiving vessel filled with alkaline and iodine solutions, where it was trapped as [^123^I]NaI.

### 4.3. Radiolabeling Optimization of PSMA-TBSB with ^123^I

The ^123^I was available in 0.01 M NaOH with an average batch activity of approx. 732 MBq/mL. The average ^123^I activity used for each experiment was 25 MBq. An amount of PSMA solution (1 mg/mL previously dissolved in CH_3_OH/CH_3_COOH; 95/5 (*v*/*v*)) was added to the ^123^I solution. Chloramine-T (10 μL in Milli-Q water) was used as an oxidizing agent. The reaction was performed at room temperature. The reaction time was calculated from the time chloramine-T was added to the mixed solution and vortexed carefully. To quench the reaction, 10 μL of sodium metabisulfite solution was added to the reaction mixture. The amount of sodium metabisulfite used was 2 times that of the oxidizing agent used. After that, 5 μL of NaI solution 10 mg/mL was added to the reaction mixture. To test the radiolabeling yield, a radio-iTLC was performed by spotting 2 μL onto iTLC glass microfiber chromatography sheet impregnated with a silica gel (iTLC-SG fiber sheet) and eluted with a developing solution of CH_3_CN/H_2_O, 95/5 (*v*/*v*). Under these conditions, the [^123^I]PSMA-p-IB had a Rf = 0.1–0.3, while free radioiodine moved with the front of the developing solution (R_f_ ≥ 0.75). The percent radiolabeling efficiency of the radioligand was calculated. All data are expressed as mean ± SD.

In order to investigate the optimal radiolabeling condition, the study was performed by varying the peptide amount, the reaction time, and the oxidizing agent amount. The influence of the PSMA ligand (PSMA-p-TBSB) amount (0.73 nmol, 3 nmol, 5 nmol, 10 nmol, and 50 nmol) was investigated at a fixed reaction time of 5 min and an oxidizing agent amount of 40 μg. The effect of the reaction time was studied by applying time variations of 0.5 min, 5 min, 10 min, and 30 min to the reaction process, with the peptide and the oxidizing agent amount used fixed at 5 nmol and 40 μg, respectively. Meanwhile, the study of the effect of the oxidizing agent amount on the radiolabeling optimization was conducted by using 10 μg, 40 μg, 80 μg, and 150 μg in variation with a fixed reaction time of 5 min and a peptide amount of 5 nmol. All reactions were carried out at room temperature, and the pH was adjusted to a value of 5–6. With each 1% addition to the CH_3_COOH solution, as much as one tenth of the volume of [^123^I]NaI was used.

### 4.4. Radiolabeling of [^177^Lu]Lu-PSMA-617

Ultrapure and metal-free buffers for radiosynthesis were prepared using high-quality Milli-Q water and pretreated with Chelex 100 resin sodium form (Sigma-Aldrich, St. Louis, MO, USA). The compound of PSMA-617 used herein was synthesized by the method as published by Benesova et al. [43]. Ammonium acetate buffer (0.2 M, pH 5.5, Merck, Darmstadt, Germany) in an amount of 80 μL was added in a LoBind Eppendorf tube containing 5 nmol of PSMA-617 in Milli-Q water (1 nmol/μL). After the addition of ^177^Lu (5 μL, 25 MBq, IRT-T Nuclear Research Reactor of Tomsk Polytechnic University), the reaction mixture was vortexed and incubated for 30 min at 80 °C. Radiochemical yield and purity were determined by using radio-iTLC and radio-HPLC methods. The iTLC method used glass-fiber sheets (Agilent Technologies, Inc., Folsom, CA, USA) eluted in 0.2 M citric acid with a pH of 2.0. Performing radio-iTLC analysis in this system provides retention of the ^177^Lu-labeled PSMA ligand molecules at the point of application, while free ^177^Lu^3+^ ions migrate with the solvent front. The HPLC technique employed for [^177^Lu]Lu-PSMA-617 was identical to that used to analyze [^123^I]PSMA-p-IB.

### 4.5. Radiochemical Purity and Shelf-Life Stability

After the optimal radiolabeling condition was achieved, the radiolabeled PSMA was purified using a Sep-Pak^®^ C18 cartridge. The cartridge was previously pre-equilibrated with 10 mL ethanol and then ethanol/water 9/10 (*v*/*v*), which was followed by passing through 10 mL Milli-Q water. Then, the radiolabeled mixture was loaded into the C18 cartridge. The cartridge was rinsed by passing through 10 mL of Milli-Q water 3 times. The [^123^I]PSMA-p-IB was purified by passing through 1 mL of ethanol, and the drops coming from the cartridge were collected.

The purity of the [^123^I]PSMA-p-IB was determined by performing radio-iTLC and radio-HPLC. Radio-iTLC was performed using TLC silica gel 60 F_254_ aluminium plates (iTLC-SG 60 F_254_) with a developing solution of CH_3_CN/H_2_O, 95/5 (*v*/*v*). Radio-HPLC was performed using Agilent 1200 Series HPLC Systems (Agilent Technologies, Santa Clara, CA, USA) with a Luna C18(2) column 5 μm, 100 Å, 250 × 4.6 mm. The radio detector was the Raytest Gabi Star 20.04.09 Firmw with Serial No. 30685. The concentration gradient was 0 min, 95% A (5% B); 5 min, 80% A (20% B); 10 min, 65% A (35% B); 15 min, 50% A (50% B); 25 min, 20% A (80% B); 30 min, 0% A (100% B); 32 min, and 95% A (5% B); where system A = 0.1% TFA in water, and system B = 0.1% TFA in acetonitrile, with a flow rate of 1 mL/min. The [^177^Lu]Lu-PSMA-617 purity was also determined by radio-iTLC and radio-HPLC.

The shelf-life stability of [^123^I]PSMA-p-IB with ethanol solvent was examined by storing it in a fridge for three days. After three days of storage, the sample’s stability was assessed using radio-HPLC.

### 4.6. Lipophilicity Assay: Log(D)

Lipophilicity of ^123^I-PSMA was determined as the logarithm of the partition coefficient, log(D), of the ^123^I-PSMA compound between n-octanol and water [44]. A volume of 500 μL of n-octanol was added to an Eppendorf tube containing the same volume of Milli-Q water. An amount of 10 pmol of ^123^I-PSMA was added to the Eppendorf tube containing n-octanol and Milli-Q water. The mixture was vigorously vortexed for 3 min and then centrifuged for 5 min. The activity concentration of 100 μL of both phases was then measured by a gamma counter. Each measurement was repeated in triplicate.

### 4.7. In Vitro Cell-Binding Assay

The binding-specificity test for [^123^I]PSMA-p-IB was performed against two human prostate cancer cell lines, PSMA-positive PC-3 PIP and PSMA-negative PC-3. The PSMA-expressing isogenic human prostate carcinoma PC-3-PIP cell line was obtained from Dr. Warren Heston, Cleveland Clinic. The PC-3 cell line was purchased from the American Type Culture Collection (ATCC; LGC Promochem, Borås, Sweden). The cell lines were cultured as published by Lundmark et al. [38].

Two sets (six wells) of dishes were used for each cell line. One day prior to the experiment, three dishes containing approximately 0.7 × 10^6^ cells per dish were seeded. To one set of dishes (three dishes) for each cell line, a 500-fold molar excess of unlabeled PSMA ligand was added to saturate PSMA receptors 30 min before adding the [^123^I]PSMA-p-IB. An equal volume of complete media was added to another set of dishes for each cell line. Then, all cells were incubated with a 1 nM concentration of [^123^I]PSMA-p-IB for 1 h at 37 °C. After incubation, the medium and 1 mL of PBS solution that was used to wash the dish were collected. The cells were detached by treatment with 500 μL trypsin and incubated for 10 min at 37 °C, and these were then collected into tubes. Cell-associated radioactivity was measured using a gamma counter and displayed as a percentage of cell-associated activity.

### 4.8. Affinity Measurements Using Saturation-Binding Experiments

The binding kinetics of [^123^I]PSMA-p-IB to living PC-3 PIP cells was measured by the equilibrium dissociation constant (K_D_) using saturation-binding experiments. Several concentrations in the range of ~0.1 × K_D_ to ~10 × K_D_ were used in this experiment. The radioactive ligand was added from the same stock solution. To achieve a lower concentration, less of the radioactive solution was added to the dishes, and media was added to compensate for the rest of the volume. Four dishes (3 non-blocked and 1 blocked) were used for each concentration. The blocked dish was used to account for non-specific binding. Twenty (20) μM of unlabeled ligand was added to each of the blocked dishes. Incubations were conducted at 4 °C for 4 h. The solution was removed from the cell after incubation, followed by rinsing. Then, 500 μL of trypsin was added to the cell dish, and discharge of the cells was awaited. Following the discharge of all cells, 1 mL of medium was added to each dish. Afterwards, one-third of the sample volume was taken to the cell counter, and the remaining two-thirds of the sample volume was measured using the gamma counter. The K_D_ was determined using a nonlinear regression analysis implemented in GraphPad Prism.

### 4.9. In Vivo Biodistribution

All applicable international and national guidelines of the Russian Federation for the care and the use of the animals were followed during the planning and execution of the animal experiments. The animal study protocol was approved by the Ethics Committee of Siberian State Medical University, Tomsk, Russia (protocol code 2, 20220927).

The biodistribution of [^123^I]PSMA-p-IB and [^177^Lu]Lu-PSMA-617 in normal mice was evaluated in 8 female CD1 mice (2 groups) of 6 weeks of age with an average weight of 31.8 ± 3.6 g. The mice were housed and cared for under standard conditions prior to use. Four mice were each injected intravenously through the tail vein with 40 kBq (80 pmol) of [^123^I]PSMA-p-IB in 100 μL PBS with 10% ethanol. The remaining 4 mice were intravenously (i.v.) injected with 130 kBq (80 pmol) of [^177^Lu]Lu-PSMA-617 in 100 μL PBS with 1% BSA per mouse. The mice were sacrificed 4 h after injection. Cervical dislocation was employed to sacrifice the anesthetized mice. The blood, tissues, and organs (salivary gland, lung, liver, spleen, small intestine, kidney, muscle, and bone) were excised, collected, and weighed. The ^123^I and ^177^Lu activities were simultaneously measured using a gamma-counter. The activity uptake was expressed as the percentage of injected activity per gram of organ (%ID/g).

## 5. Conclusions

Two novel DCL urea-based PSMA inhibitors with a chlorine-substituted aromatic fragment at the lysine ε-nitrogen atom, an L-Phe-L-Phe dipeptide linker, and 3- or 4-(tributylstannyl)benzoic acid as prosthetic groups for radioiodination were synthesized using two alternative synthesis schemes. These inhibitors were studied as novel PSMA ligands by conducting radiolabeling optimization with iodine-123. The [^123^I]PSMA-p-IB ligand was tested in the initial preclinical evaluation. The novel PSMA-targeting radioligand [^123^I]PSMA-p-IB demonstrated a considerable affinity and specific binding to PSMA-expressing cells in vitro. Low accumulation in normal organs during an in vivo test indicates that this novel PSMA inhibitor has the potential to be a promising novel PSMA-targeting radioligand that warrants further study.

## Figures and Tables

**Table 1:** Comparison of alternative methods for obtaining conjugate **21** (Scheme 4).

Parameters	Way A	Way B
Yield of iodized product by prosthetic group (based on m-STBSB)	57.6%	48.8%
Number of stages with peptidomimetic/number of stages with ^123^I	1/2	2/1

**Table 2:** Radiochemical yield from radiolabeling optimization studies of the [^123^I]PSMA-p-IB ligand. Radiochemical yields were based on iTLC SG fiber-sheet analysis.

Variation of PSMA-p-TBSB Amounts *		Variation of Reaction Time **		Variation of Oxidizing Agent Amount ***	
Amount of Peptide (nmol)	RCY (%)	Time (min)	RCY (%)	Amount of Chloramine-T (μg)	RCY (%)
0.73	40.9 ± 0.5	0.5	61.9 ± 0.4	10	61.3 ± 0.5
3	54.7 ± 0.8	5	69.2 ± 0.2	40	69.2 ± 0.2
5	69.2 ± 0.2	10	71.7 ± 0.3	80	69.4 ± 0.5
10	75.9 ± 1.0	30	70.6 ± 0.1	150	60.7 ± 0.4
50	72.8 ± 0.4				

* Reactions were carried out using a fixed amount of chloramine-T (40 μg) and for a fixed time of 5 min. ** Reactions were carried out using a fixed amount of PSMA-p-TBSB (5 nmol) and chloramine-T (40 μg). *** Reactions were carried out using a fixed amount of PSMA-p-TBSB (5 nmol) and for a fixed time of 5 min.

## Data Availability

The data generated during the current study are available from the corresponding author upon reasonable request.

## Supplementary Materials

The following supporting information can be downloaded at: https://www.mdpi.com/article/10.3390/ijms241512206/s1.
