# Supplementary material for: Synthesis, 123I-Radiolabeling Optimization, and Initial Preclinical Evaluation of Novel Urea-Based PSMA Inhibitors with a Tributylstannyl Prosthetic Group in Their Structures
Source: Int J Mol Sci. 2023 Jul 30;24(15):12206. doi: 10.3390/ijms241512206 (PMC10418939; doi:10.3390/ijms241512206)
Supplement: Supplementary file 1 [file ijms-24-12206-s001.zip › ijms-2506591-supplementary.pdf]

Synthesis, ^{123}I-radiolabeling optimization, and initial preclinical evaluation of novel urea-based PSMA inhibitors with a tributylstannyl prosthetic group in their structures

Lutfi A. Hasnowo [1,2], Maria S. Larkina [3,4], Evgenii Plotnikov [3,5], Vitalina Bodenko [3,4], Feruza Yuldasheva [3], Elena Stasyuk [1], Stanislav A. Petrov [6], Nikolai Y. Zyk [6], Aleksei E. Machulkin [6], Nikolai I. Vorozhtsov [6], Elena K. Beloglazkina [6,*], Valentine G. Nenajdenko [6], Vladimir Tolmachev [7], Anna Orlova [8], Alexander G. Majouga [9], and Mekhman S. Yusubov [3]

1 School of Nuclear Science and Engineering, Tomsk Polytechnic University, Tomsk, 634050, Russia
2 Polytechnic Institute of Nuclear Technology, National Research and Innovation Agency, Yogyakarta, 55281, Indonesia
3 Research Centrum for Oncotheranostics, Research School of Chemistry and Applied Biomedical Sciences, Tomsk Polytechnic University, Tomsk, 634050, Russia
4 Department of Pharmaceutical Analysis, Siberian State Medical University, Tomsk, 634050, Russia
5 Mental Health Reseach Institute, Tomsk National Research Medical Center, Russian Academy of Sci-ences, 634014, Tomsk, Russia
6 Department of Chemistry, M.V. Lomonosov Moscow State University Leninskie Gory, 1-3, 119991 Moscow, Russian Federation
7 Department of Immunology, Genetics and Pathology, Uppsala University, 75185 Uppsala, Sweden
8 Department of Medicinal Chemistry, Uppsala University, 751 83 Uppsala, Sweden
9 Dmitry Mendeleev University of Chemical Technology of Russia, Miusskaya sq. 9, Moscow, 125047, Russian Federation
* Correspondence: beloglazki@mail.ru

Supplementary Materials

Table of contents

1. Radio-HPLC chromatograms of [^{123}I]PSMA-p-IB yield …… 4
2. Radio-iTLC chromatogram of [^{123}I]PSMA-p-IB yield …… 5
3. Chromatograms of [^{123}I]PSMA-p-IB after purification …… 6
4. Radio-HPLC chromatogram of [^{123}I]PSMA-p-IB after 3 days storage period …… 7
5. Radio-iTLC and radio-HPLC chromatograms of [^{123}I]PSMA-m-IB …… 8
6. ^{1}H NMR spectrum of compound № **6** …… 9
7. ^{13}C NMR spectrum of compound № **6** …… 10
8. ESI-MS of compound № **6** …… 11
9. ^{1}H NMR spectrum of compound № **9** …… 12
10. ^{13}C NMR spectrum of compound № **9** …… 13
11. ESI-MS of compound № 9 …… 14

12. HRMS (m/z, ESI) of compound № **9** 15
13. ^{1}H NMR spectrum of compound № **10** 16
14. ^{13}C NMR spectrum of compound № **10** 17
15. ESI-MS of compound № **10** 18
16. HRMS (m/z, ESI) of compound № **10** 19
17. ^{1}H NMR spectrum of compound № **11** 20
18. ^{13}C NMR spectrum of compound № **11** 21
19. ESI-MS of compound № **11** 22
20. HRMS (m/z, ESI) of compound № **11** 23
21. ^{1}H NMR spectrum of compound № **13** 24
22. ^{13}C NMR spectrum of compound № **13** 25
23. ESI-MS of compound № **13** 26
24. HRMS (m/z, ESI) of compound № **13** 27
25. ^{1}H NMR spectrum of compound № **14** 28
26. ^{13}C NMR spectrum of compound № **14** 29
27. ^{1}H NMR spectrum of compound № **16** 30
28. ^{13}C NMR spectrum of compound № **16** 31
29. HRMS (m/z, ESI) of compound № **16** 32
30. ^{1}H NMR spectrum of compound № **17** 33
31. ^{13}C NMR spectrum of compound № **17** 34
32. ^{1}H NMR spectrum of compound № **18** 35
33. ^{13}C NMR spectrum of compound № **18** 36
34. ESI-MS of compound № **18** 37
35. HRMS (m/z, ESI) of compound № **18** 38
36. ^{1}H NMR spectrum of compound № **19** 39
37. ^{13}C NMR spectrum of compound № **19** 40
38. HRMS (m/z, ESI) of compound № **19** 41
39. ^{1}H NMR spectrum of compound № **20** 42
40. ^{13}C NMR spectrum of compound № **20** 43

41. HRMS (m/z, ESI) of compound № **20** 44
42. ^{1}H NMR spectrum of compound № **21** 45
43. ^{13}C NMR spectrum of compound № **21** 46
44. ESI-MS of compound № **21** 47
45. HRMS (m/z, ESI) of compound № **21** 48
46. ^{1}H NMR spectrum of compound № **22** 49
47. ^{13}C NMR spectrum of compound № **22** 50
48. ESI-MS of compound № **22** 51
49. HRMS (m/z, ESI) of compound № **22** 52

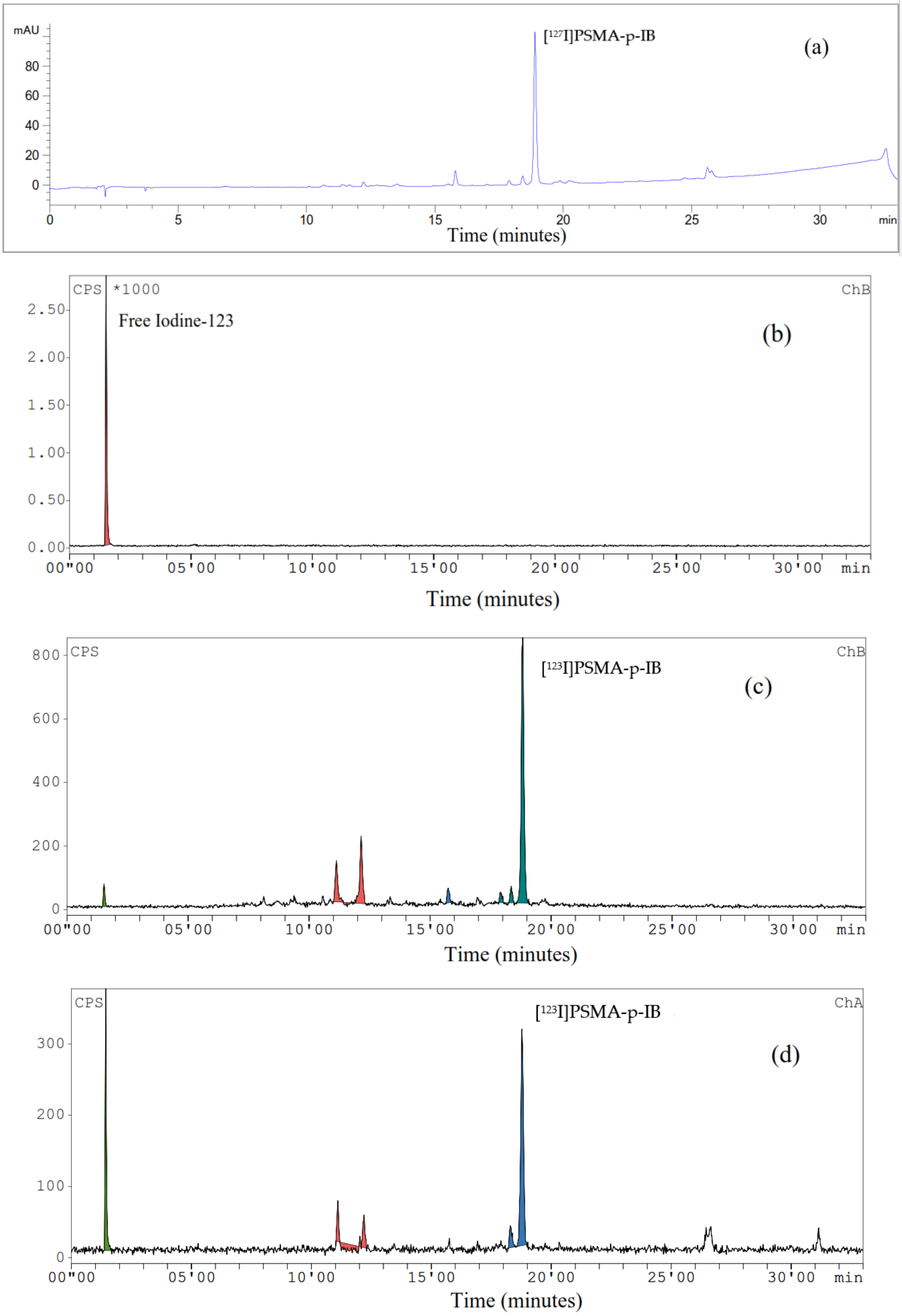

Figure S1. Radio-HPLC chromatograms of (a) [^{127}I]PSMA-p-IB reference sample, (b) blank solution, (c) radiolabeling yield with [^{123}I]PSMA-p-IB yield of 69.99%, and (d) radiolabeling yield with [^{123}I]PSMA-p-IB yield of 65.73%.

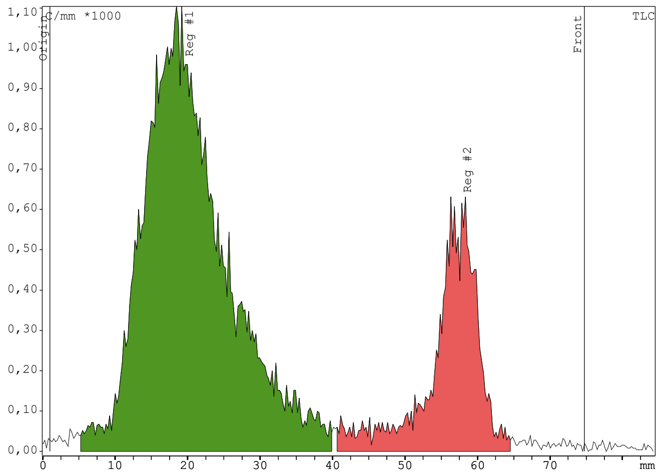

Figure S2. Radio-iTLC chromatogram of [^{123}I]PSMA-p-IB based on the results of radiolabeling optimization (the left peak: [^{123}I]PSMA-p-IB, the right peak: free Iodine-123). The chromatography was performed using iTLC-SG glass fiber sheet with a developing solution of CH_3CN/H_2O, 95/5 (v/v).

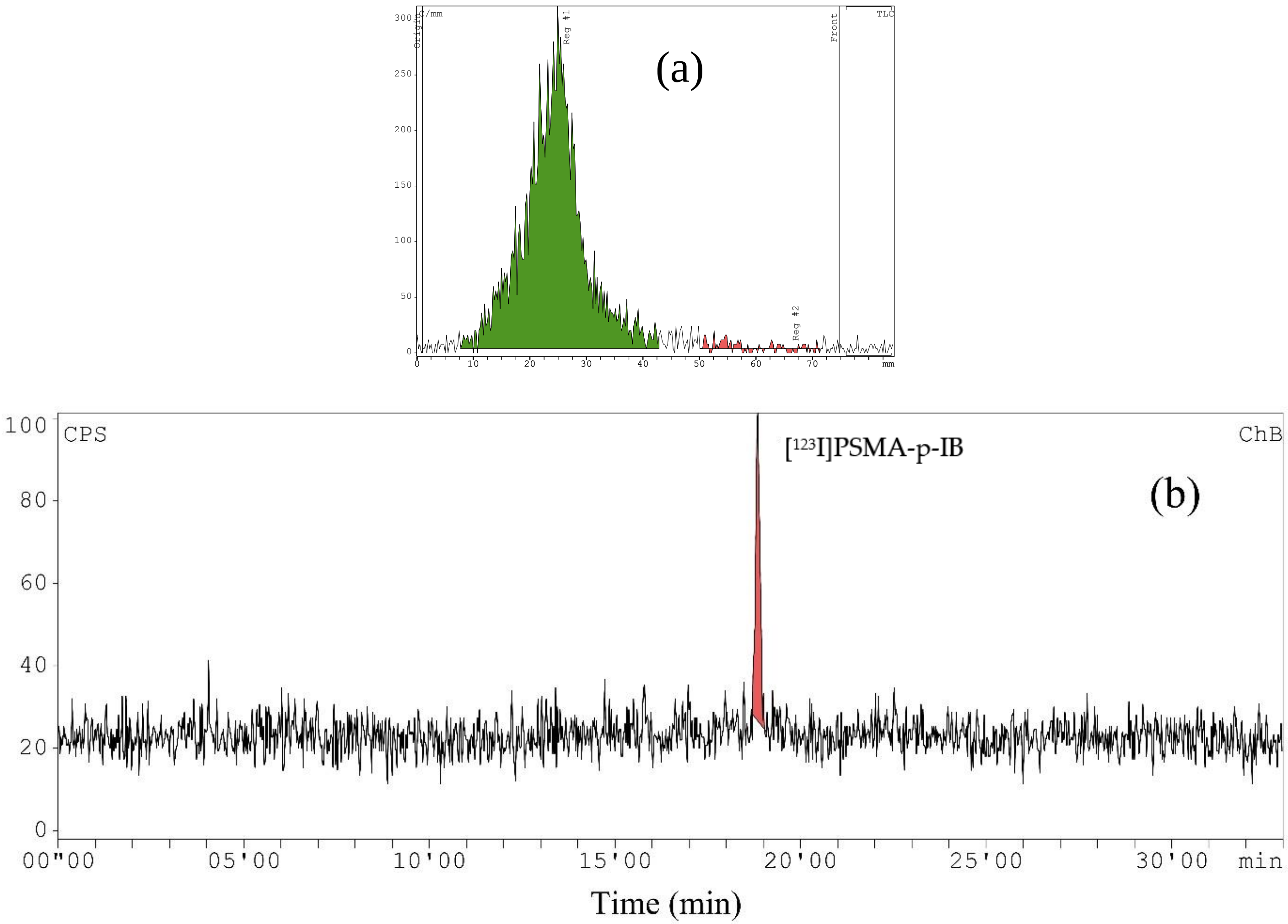

Figure S3. Chromatograms of [^{123}I]PSMA-p-IB after purification analyzed by (a) Radio-TLC-SG 60 F254 and (b) Radio-HPLC

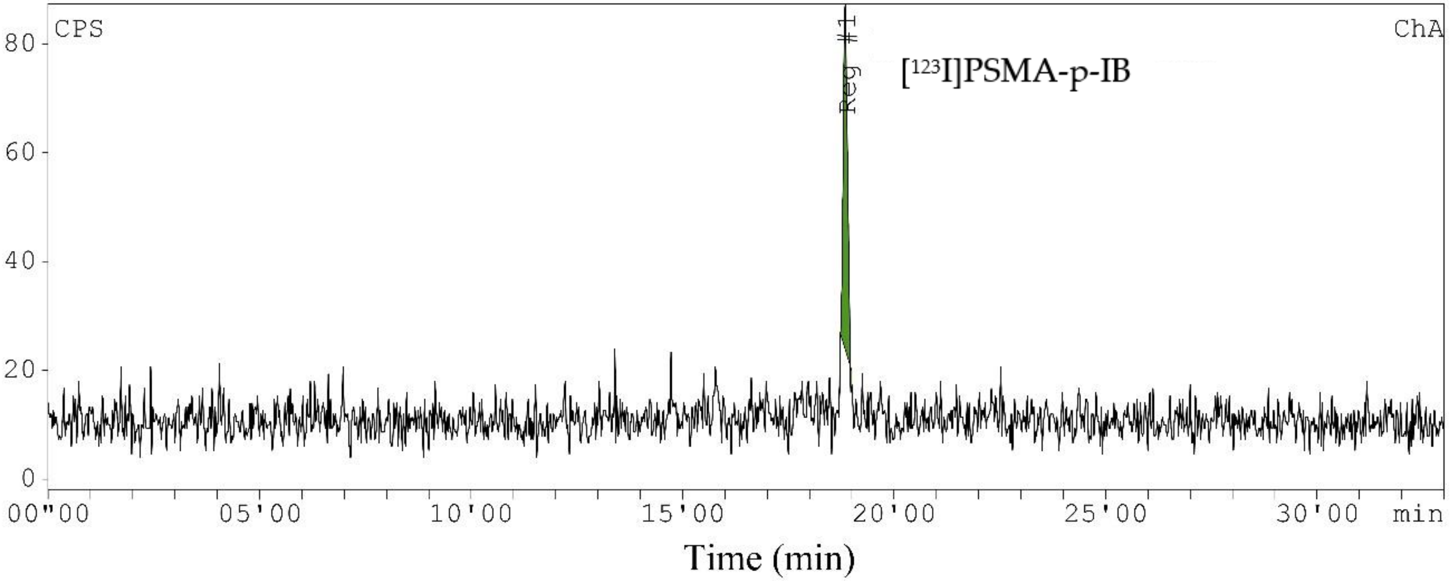

Figure S4. Radio-HPLC chromatogram of [^{123}I]PSMA-p-IB after 3 days storage period

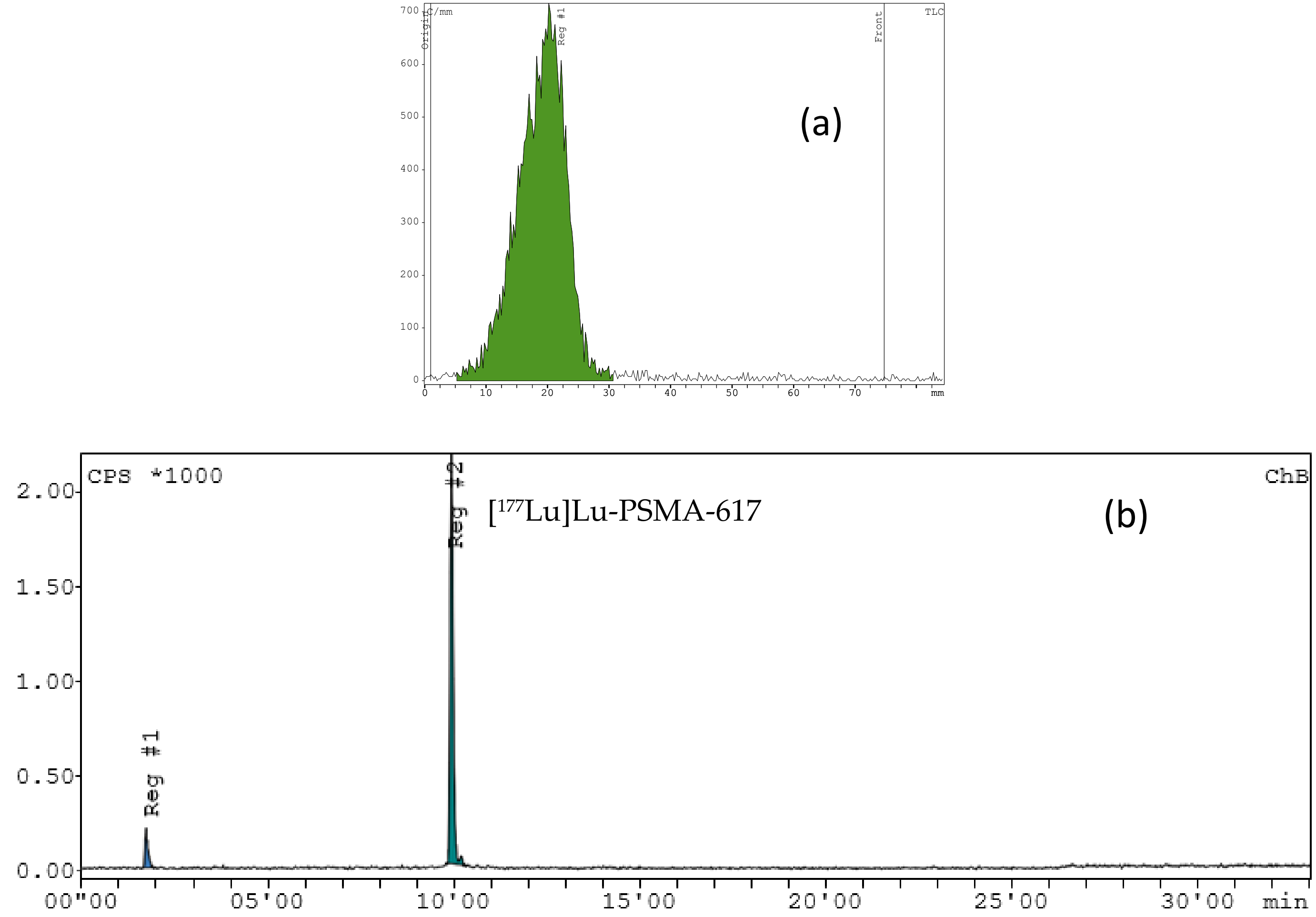

Figure S5. Chromatograms of [^{177}Lu]Lu-PSMA-617 by (a) Radio-iTLC and (b) Radio-HPLC

Compound 6

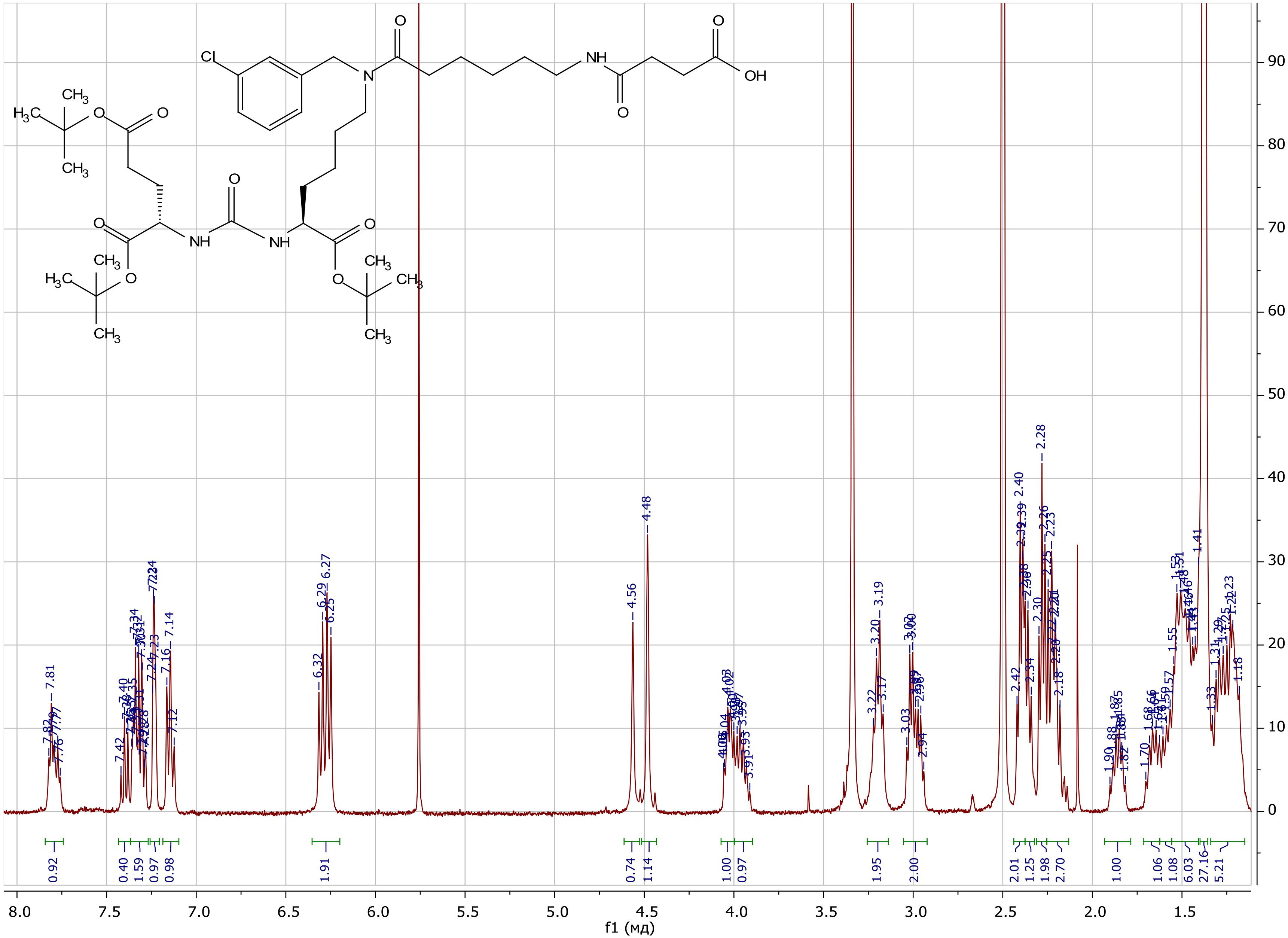

Figure S6. ^{1}H NMR spectrum of compound № **6** in DMSO-d_6.

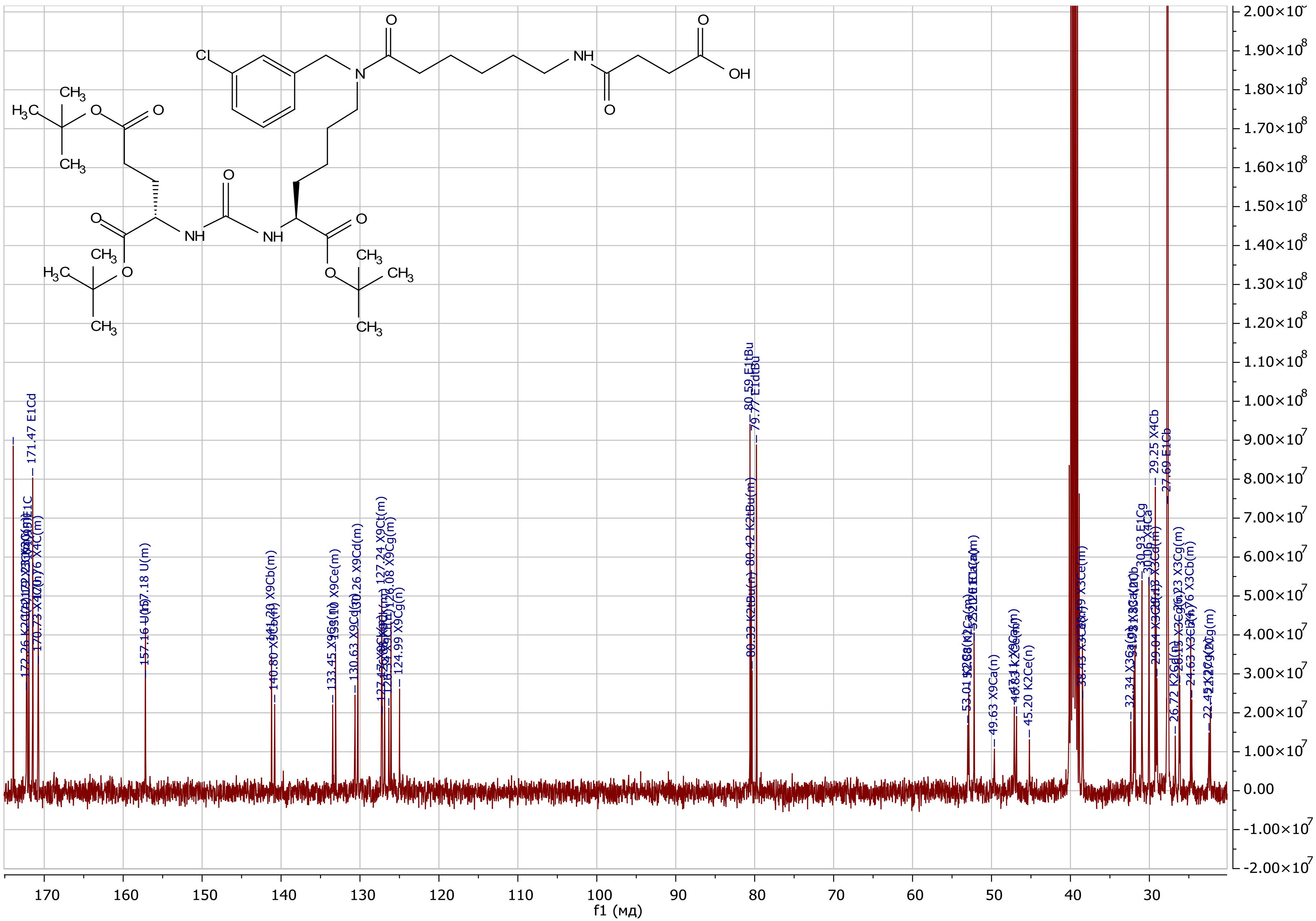

Figure S7. ^{13}C NMR spectrum of compound № **6** in DMSO-d_6.

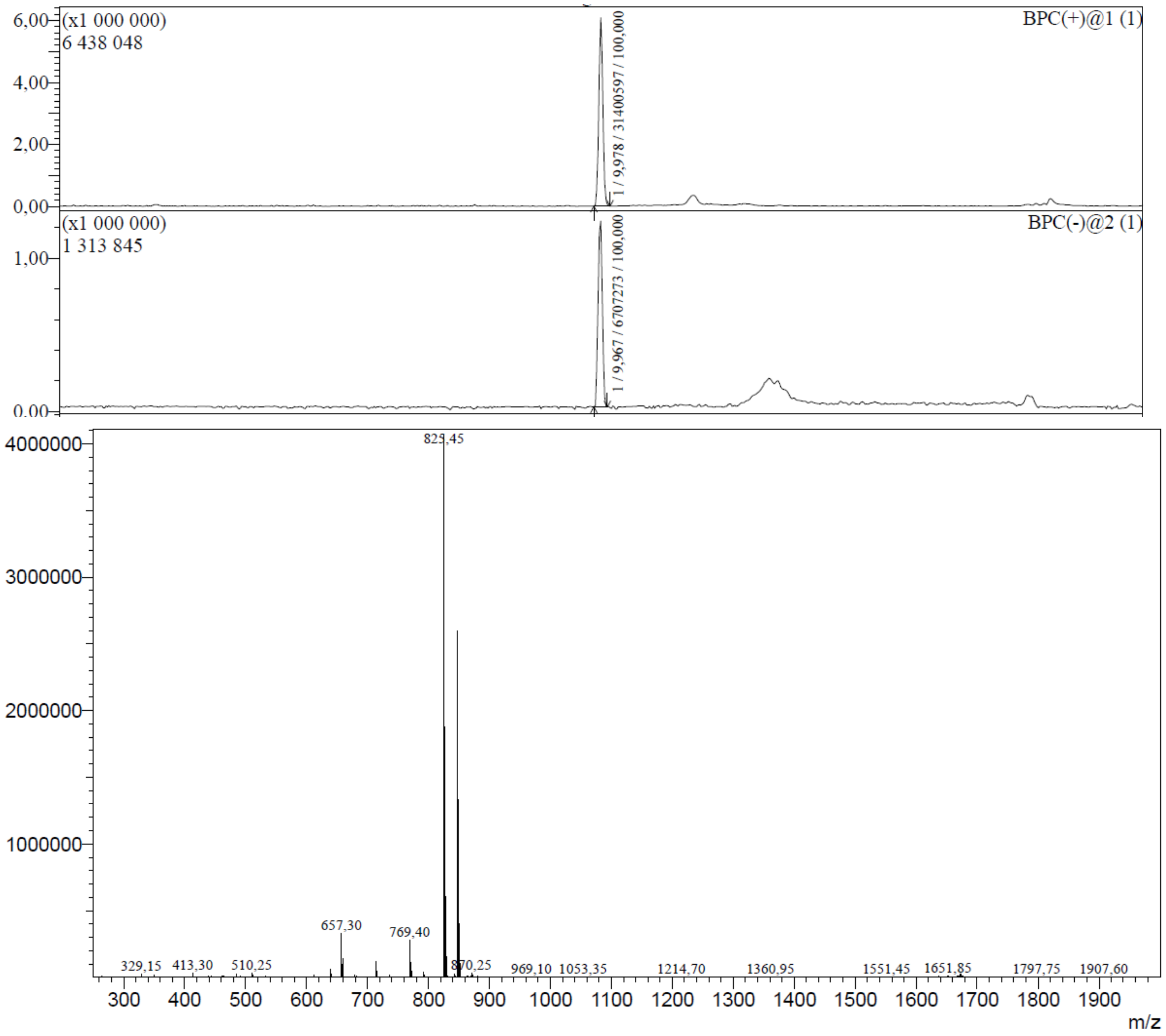

Figure S8. ESI-MS of compound № **6**

Compound 9

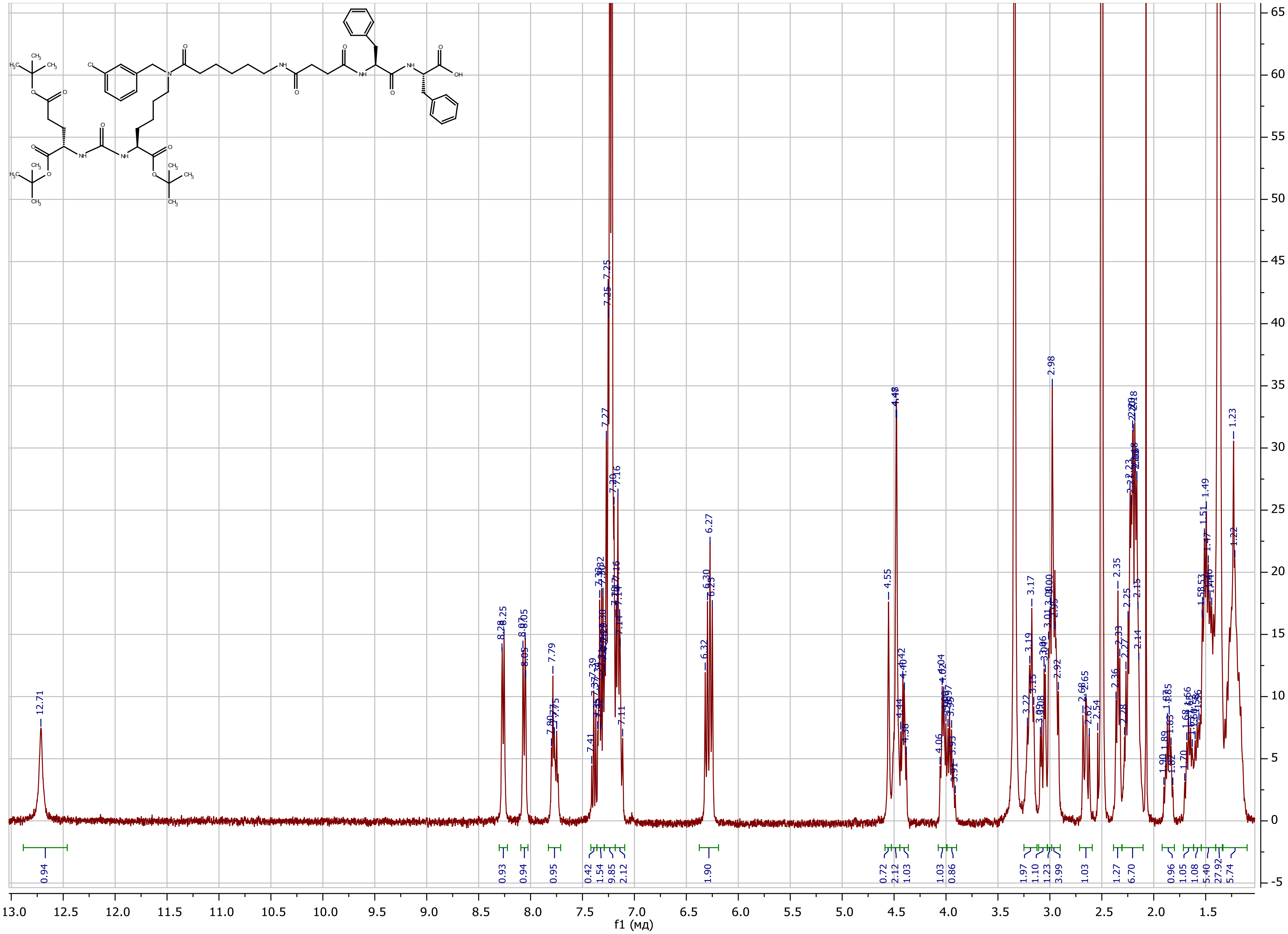

Figure S9. ^{1}H NMR spectrum of compound № **9** in DMSO-*d*6.

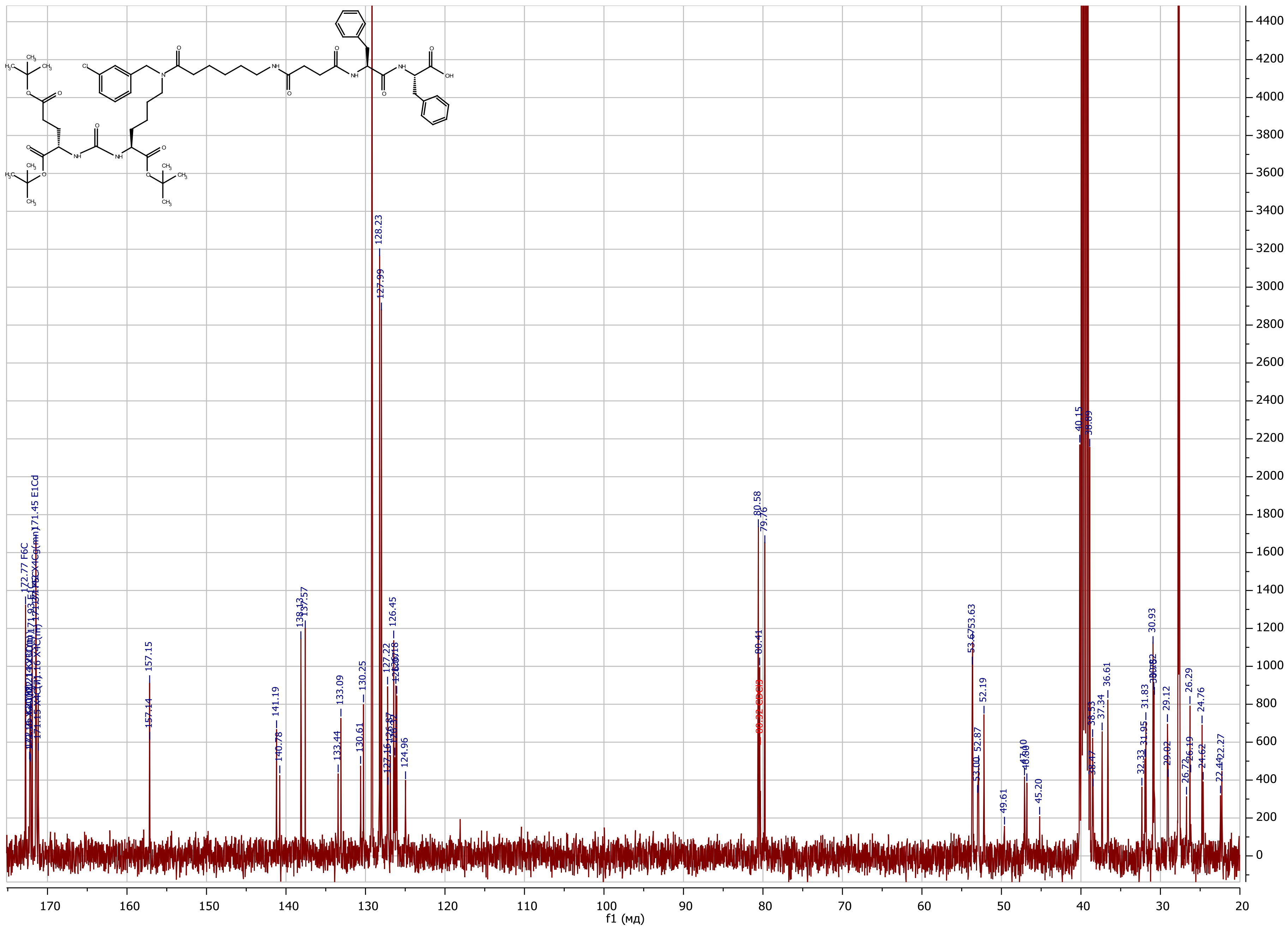

Figure S10. ^{13}C NMR spectrum of compound № **9** in DMSO-d_6.

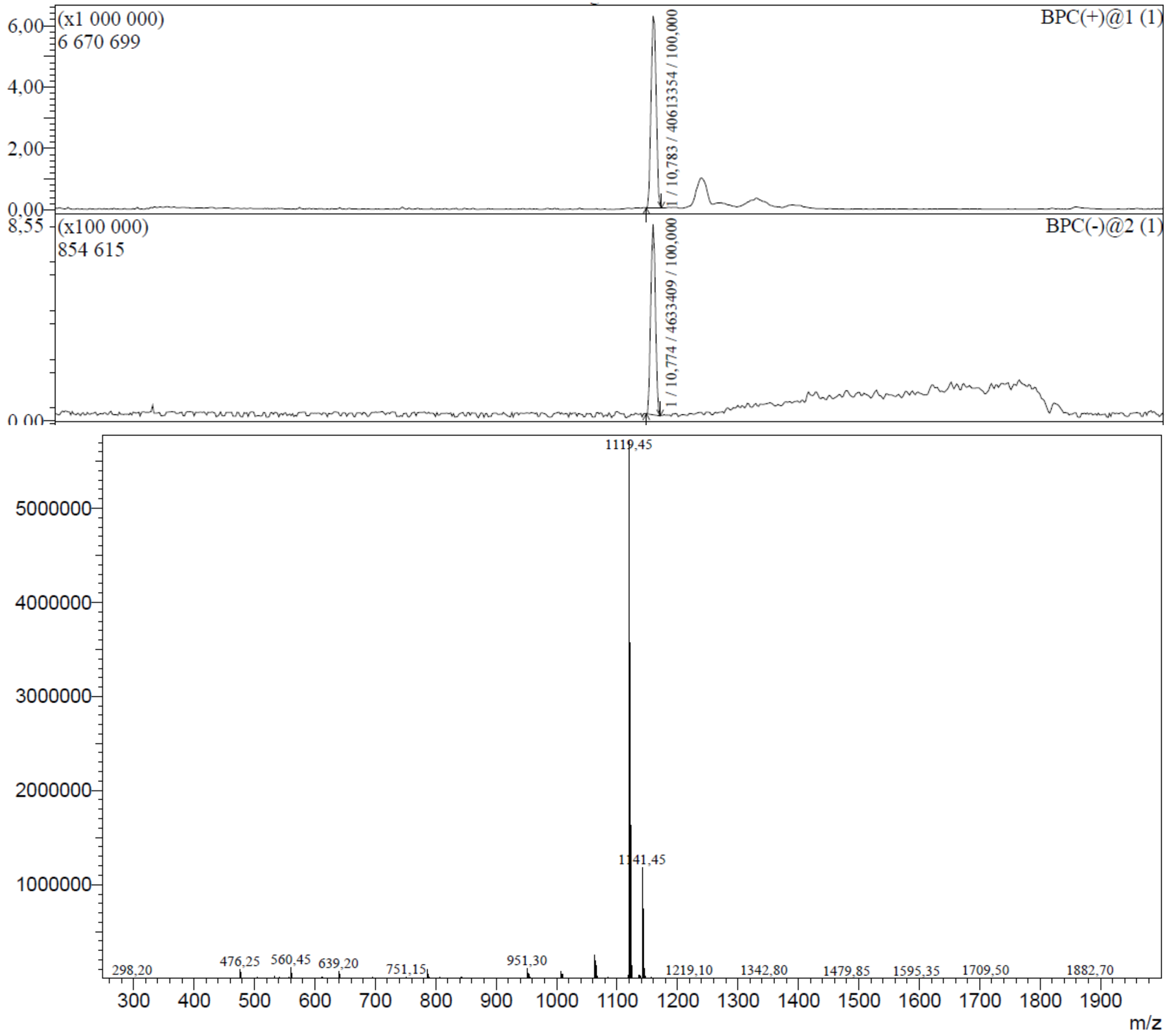

Figure S11. ESI-MS of compound № **9**

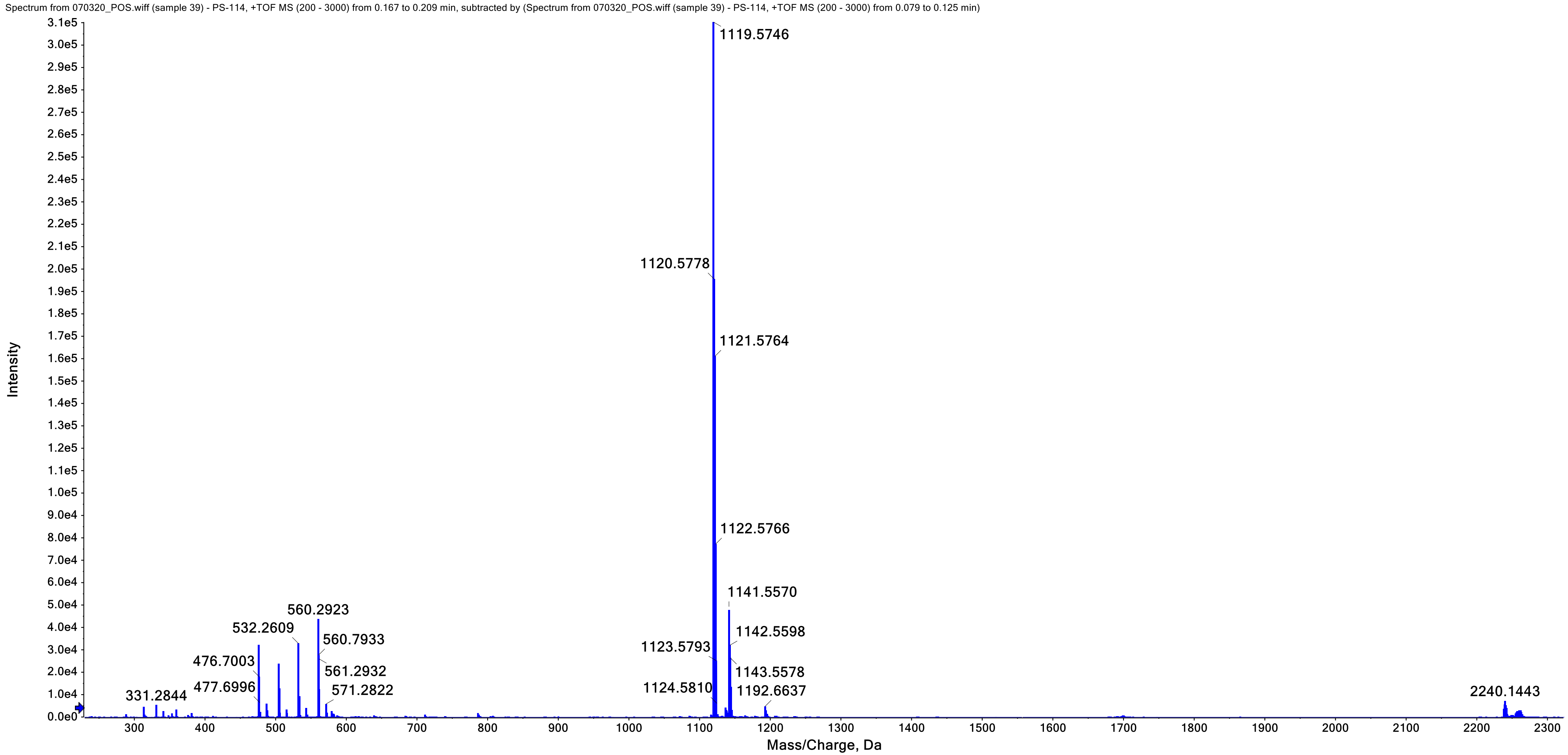

Figure S12. HRMS (m/z, ESI) of compound № **9**

Compound 10

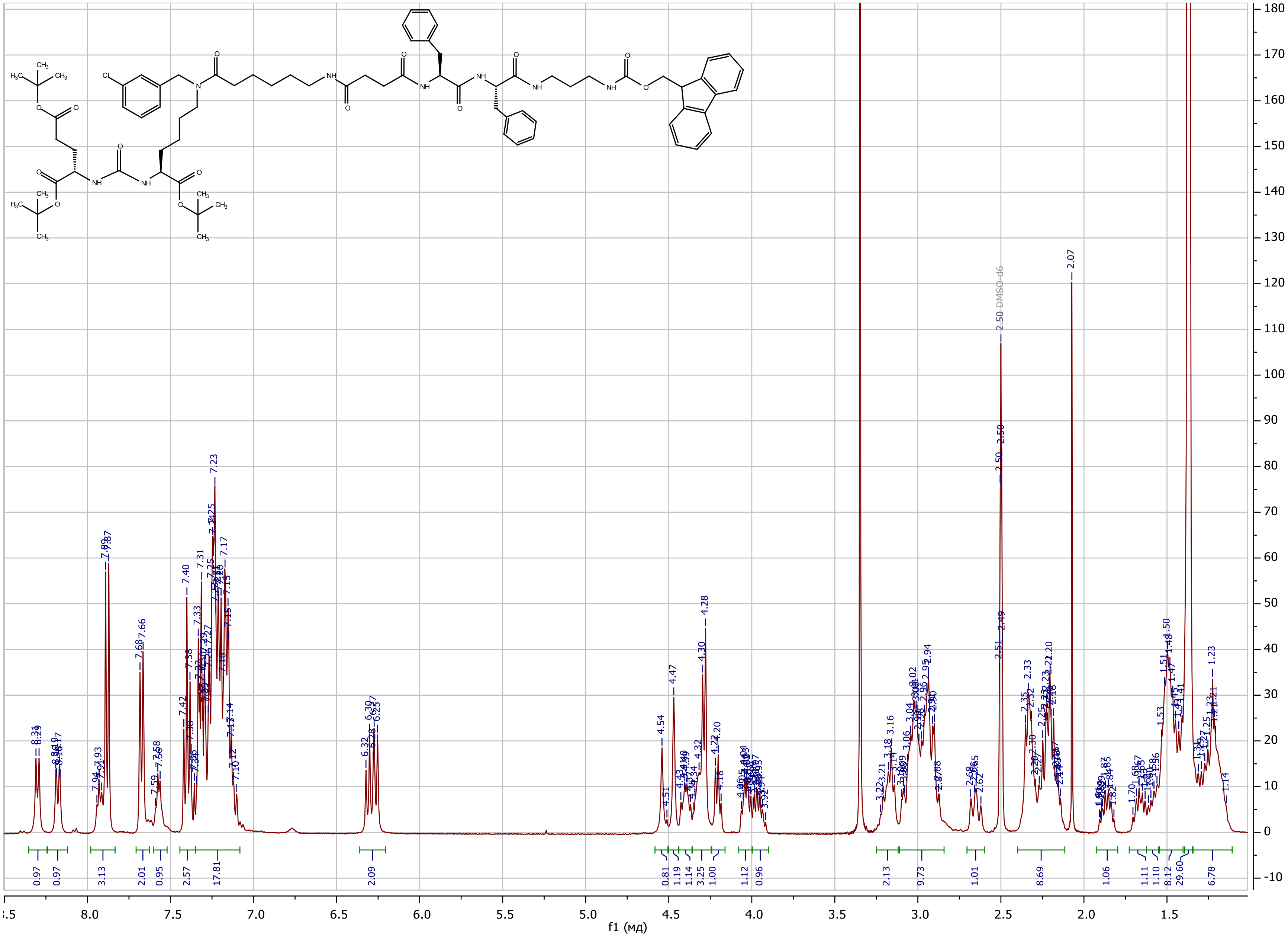

Figure S13. ^{1}H NMR spectrum of compound № **10** in DMSO-d_6.

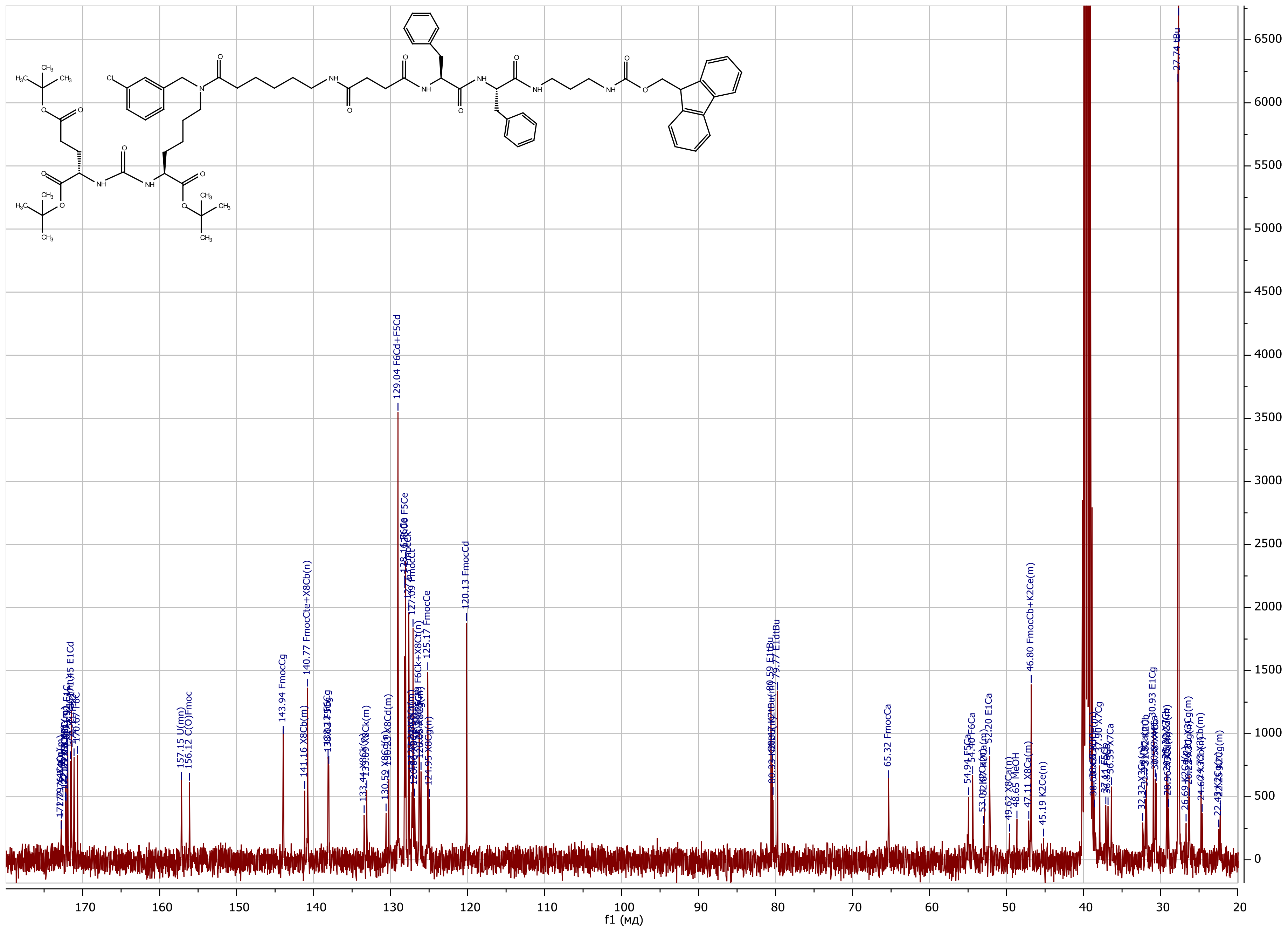

Figure S14. ^{13}C NMR spectrum of compound № **10** in DMSO-d_6.

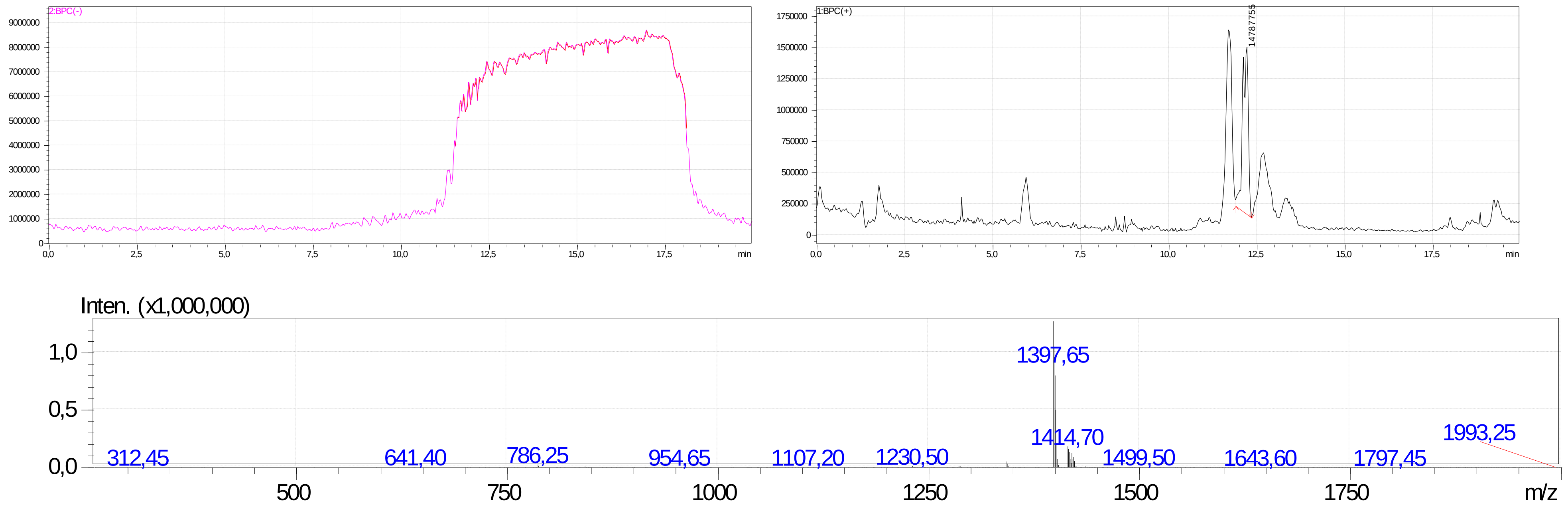

Figure S15. ESI-MS of compound № **10**

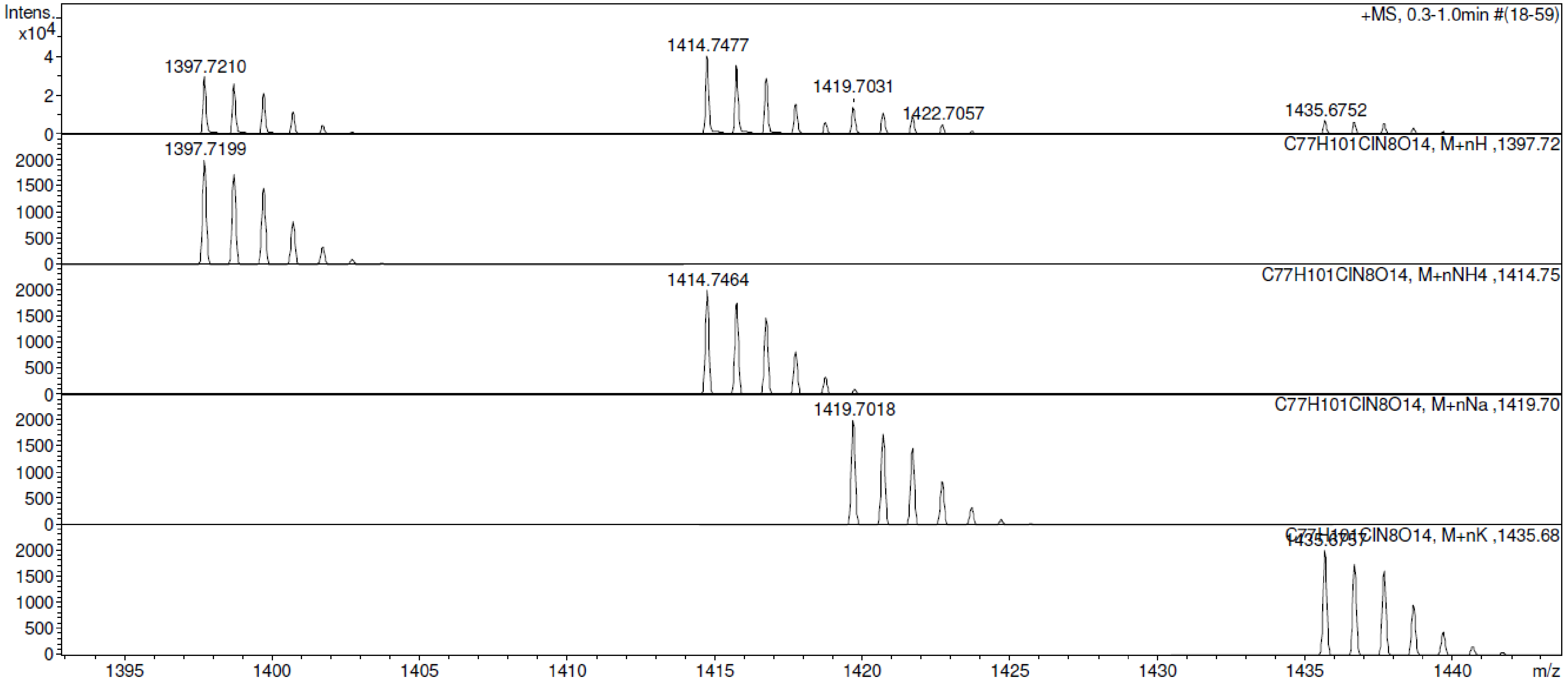

Figure S16. HRMS (m/z, ESI) of compound № **10**

Compound 11

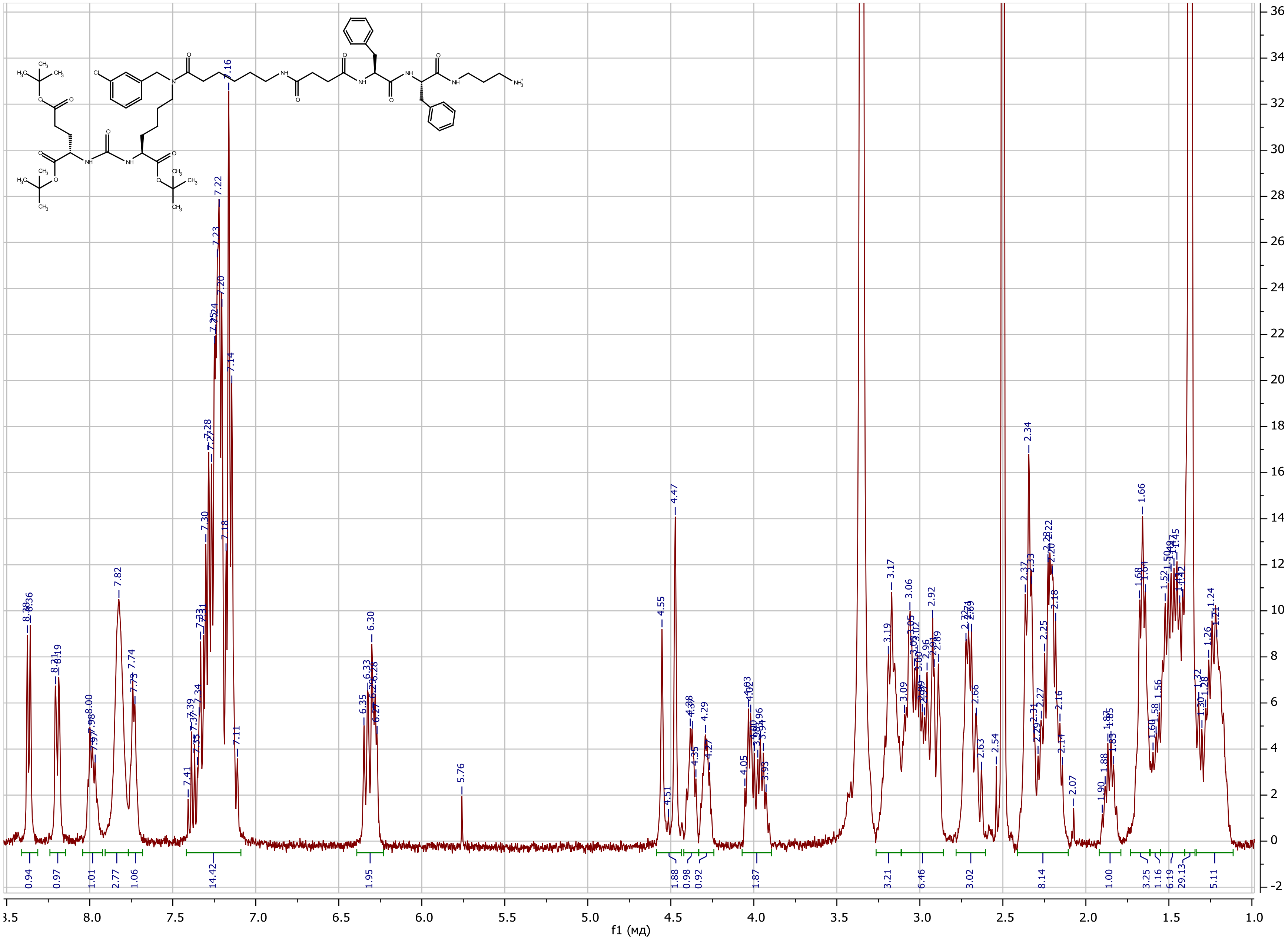

Figure S17. ¹H NMR spectrum of compound № **11** in DMSO-d_6.

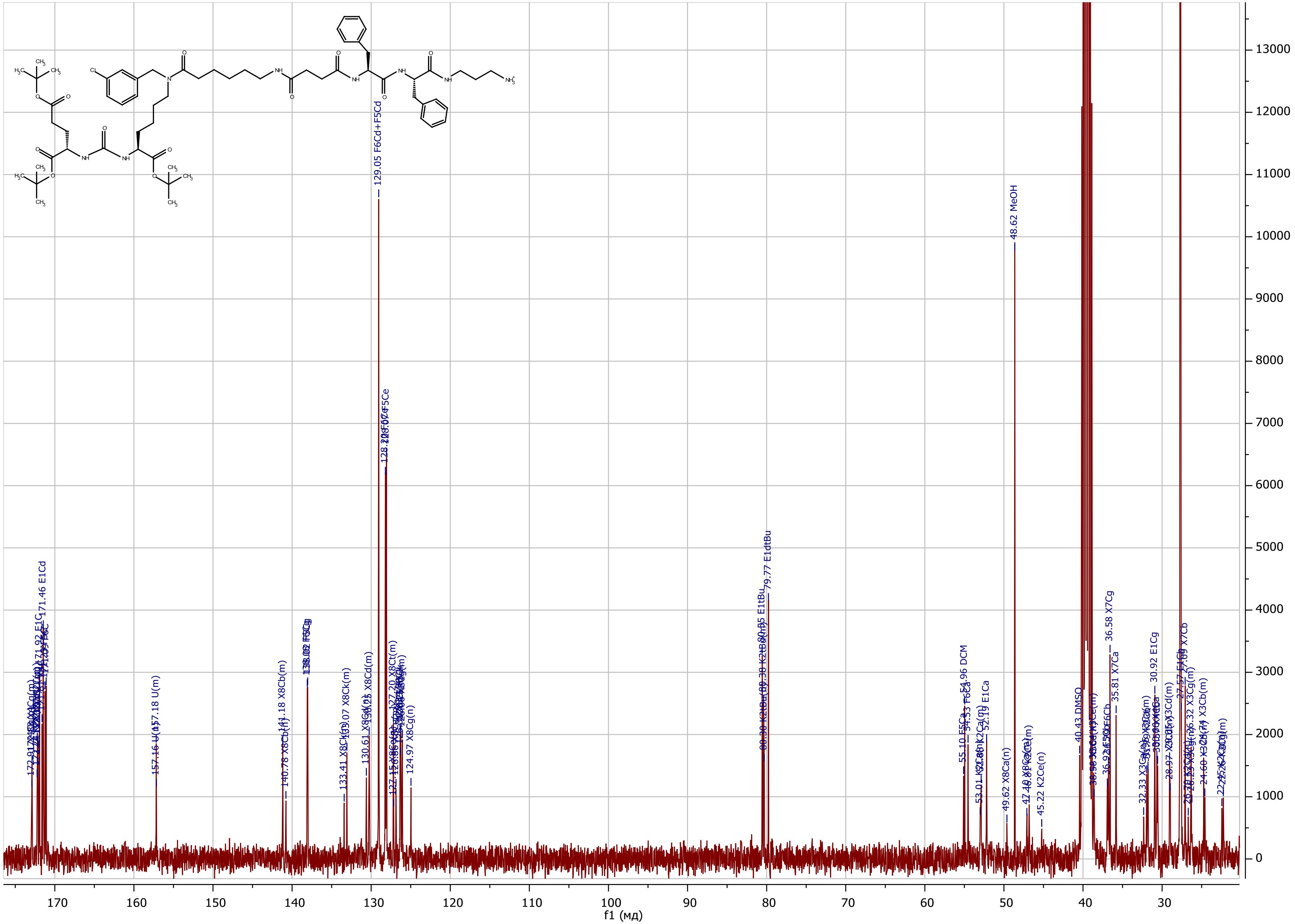

Figure S18. ^{13}C NMR spectrum of compound № **11** in DMSO-d_6.

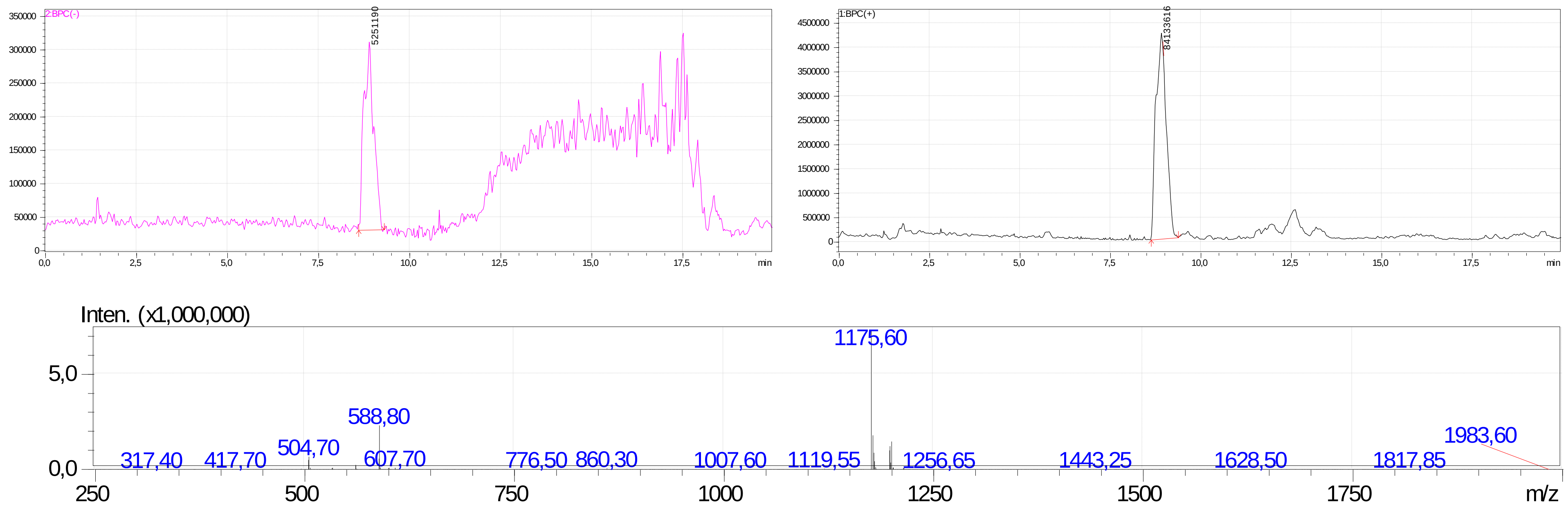

Figure S19. ESI-MS of compound № **11**

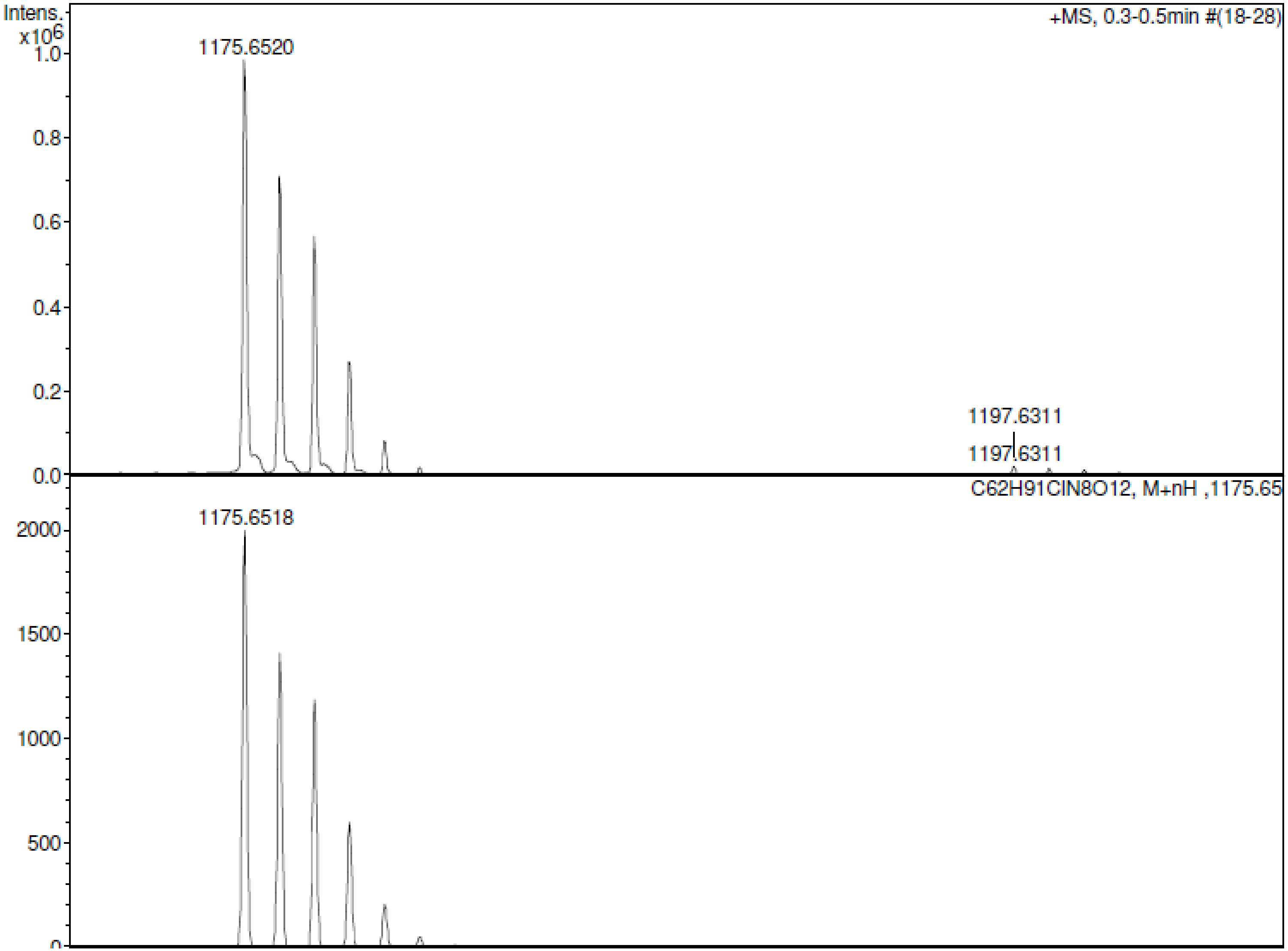

Figure S20. HRMS (m/z, ESI) of compound № **11**

Compound 13

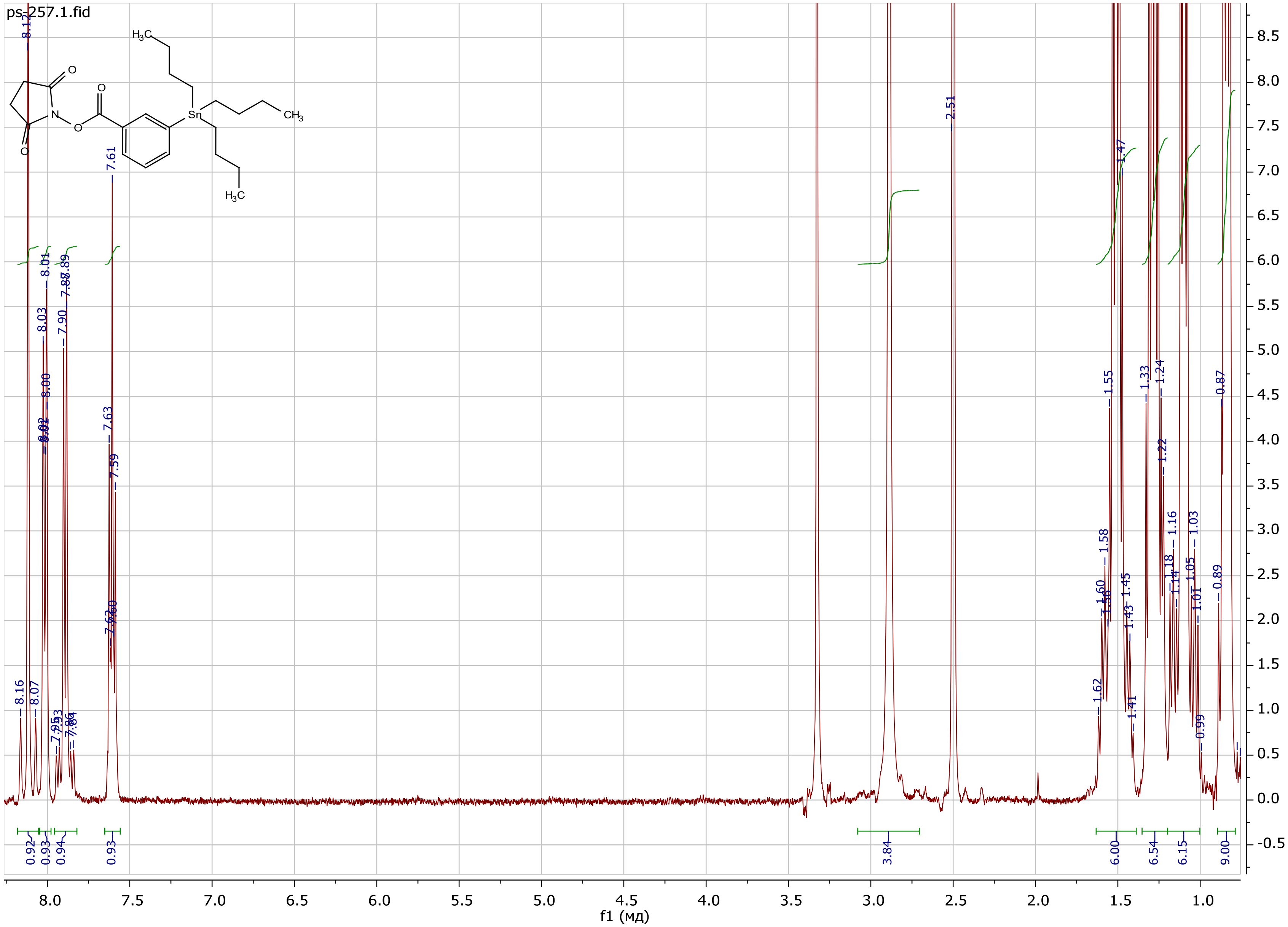

Figure S21. ^{1}H NMR spectrum of compound № **13** in DMSO-d_6.

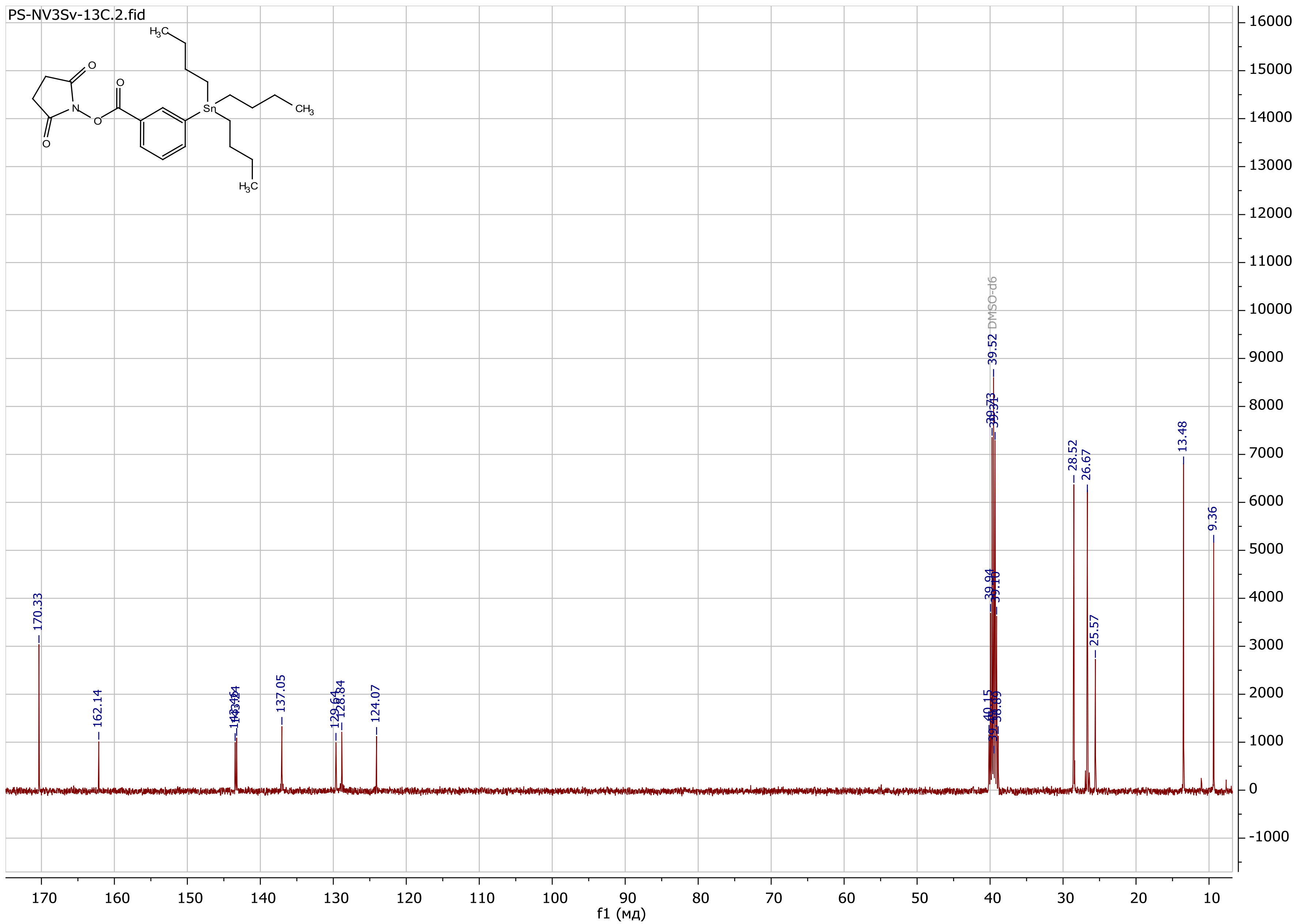

Figure S22. ^{13}C NMR spectrum of compound № **13** in DMSO-d_6.

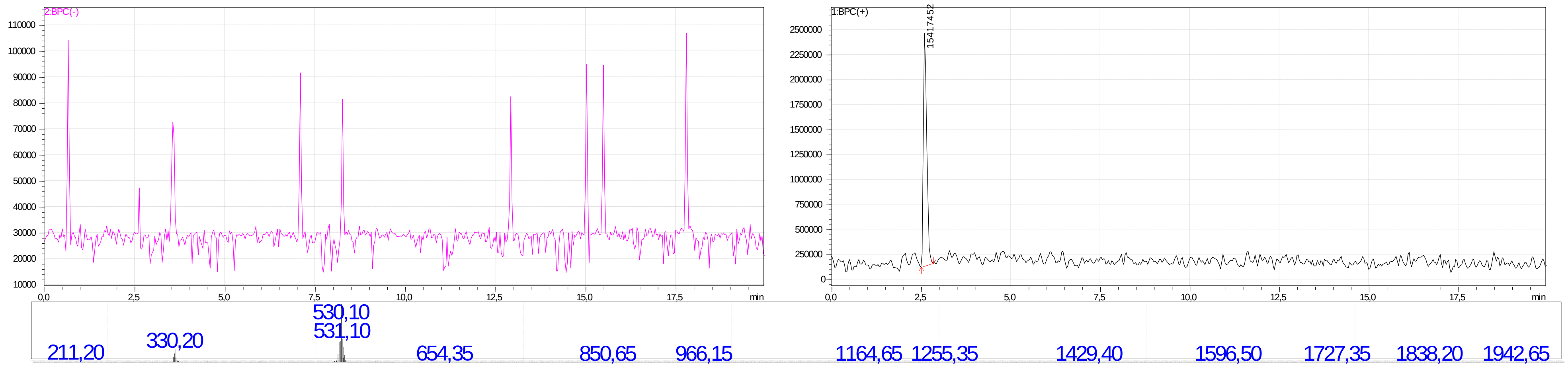

Figure S23. ESI-MS of compound № **13**

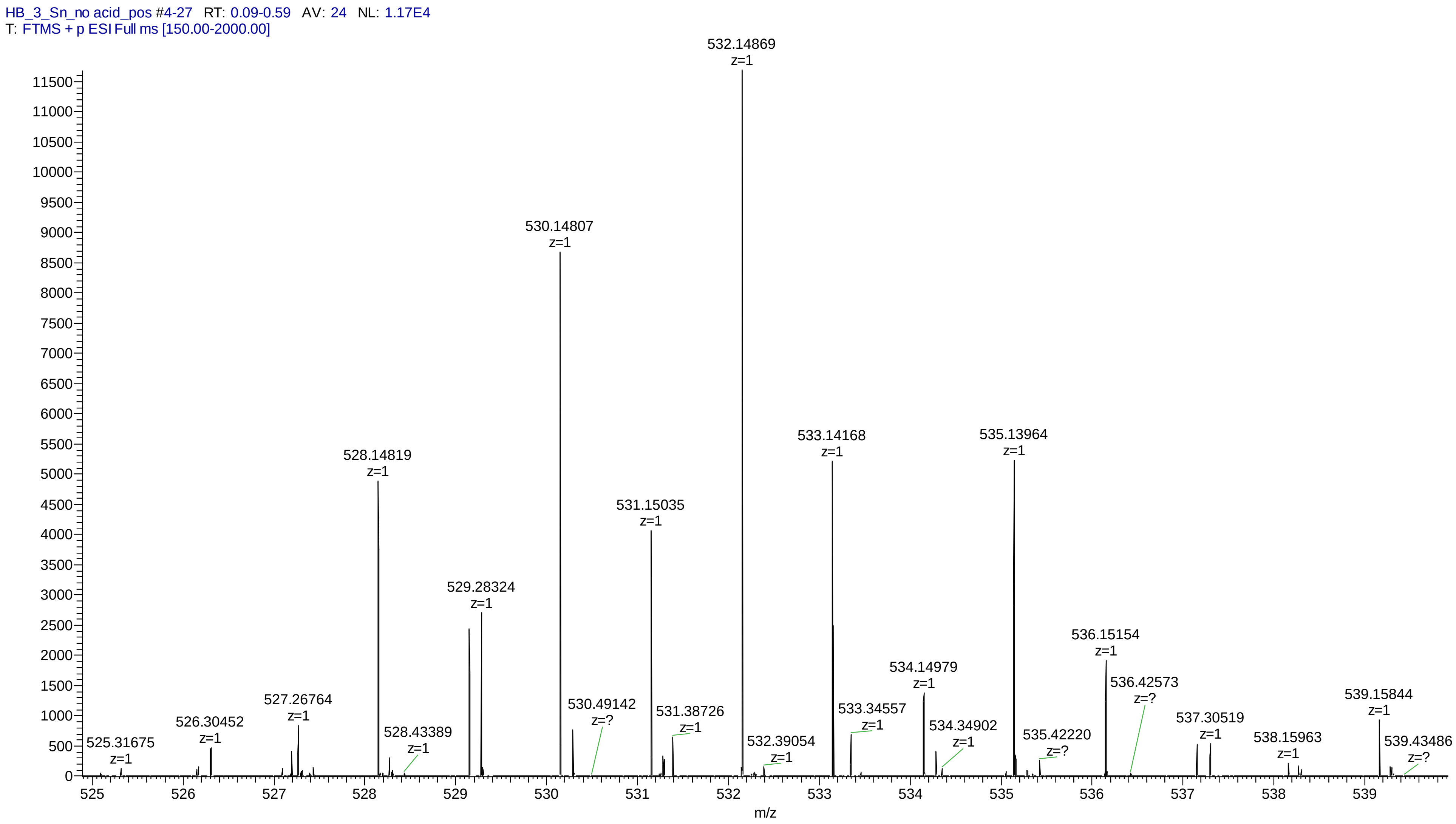

Figure S24. HRMS (m/z, ESI) of compound № **13**

Compound 14

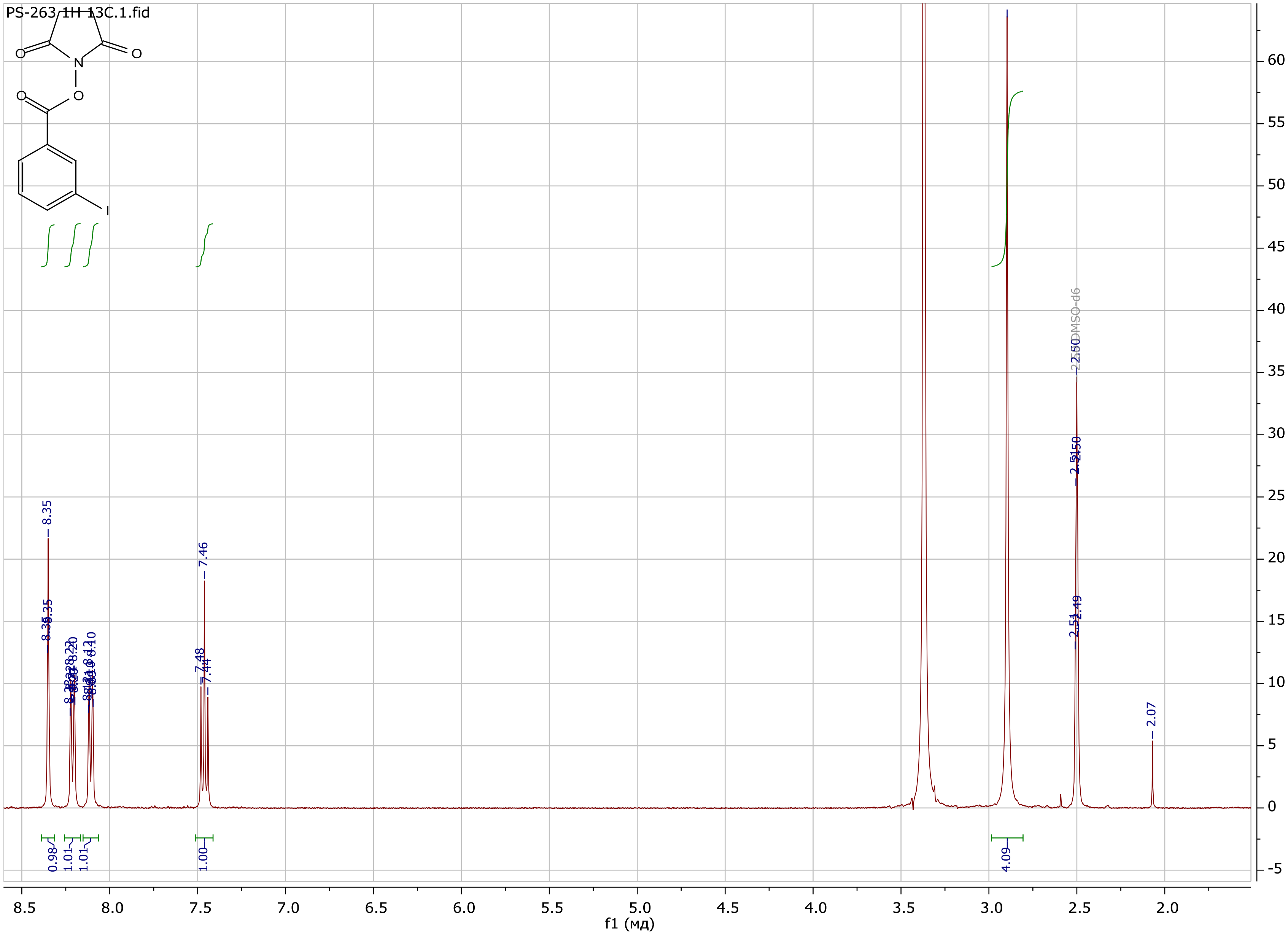

Figure S25. ^{1}H NMR spectrum of compound № **14** in DMSO-d_6.

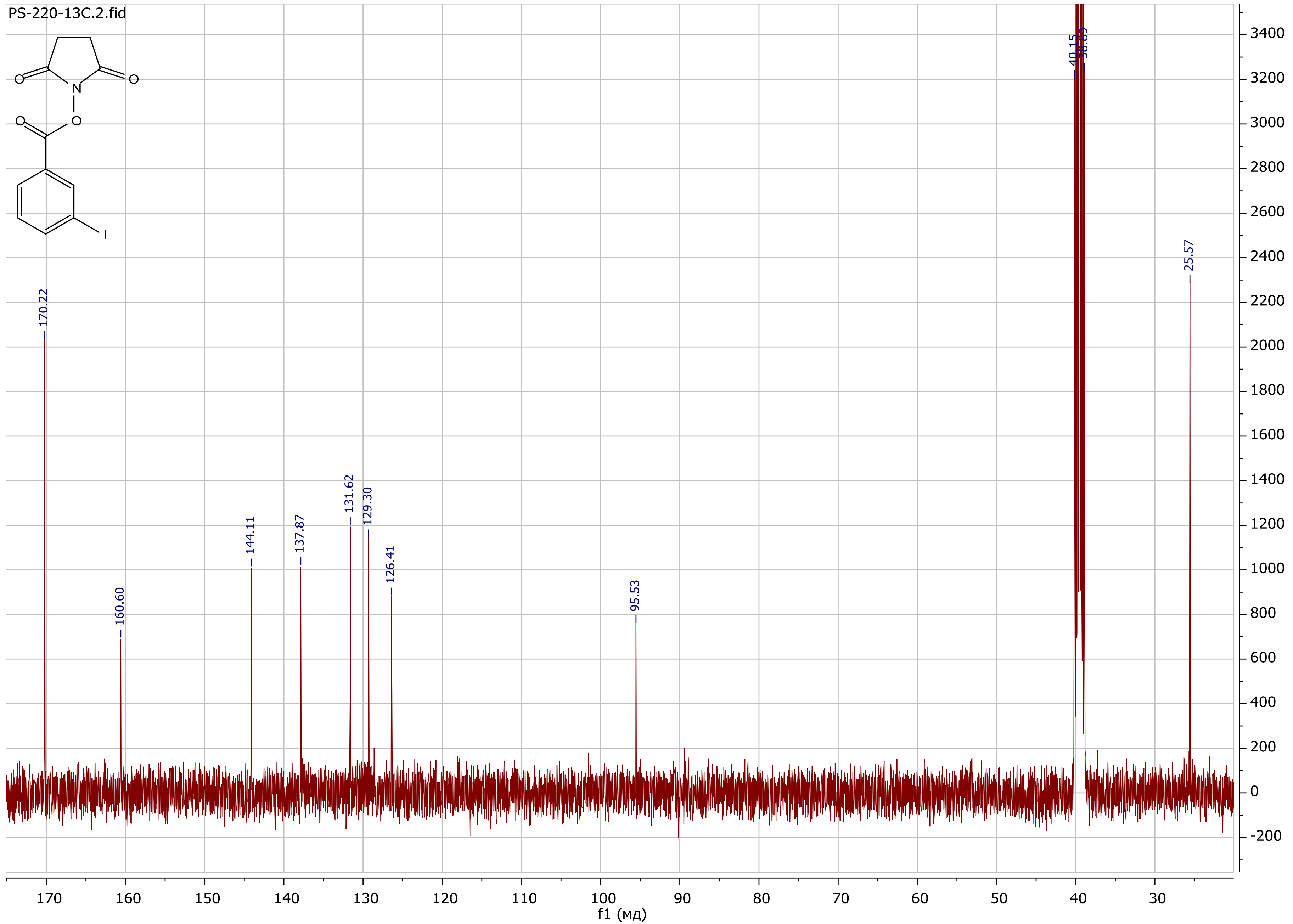

Figure S26. ^{13}C NMR spectrum of compound № **14** in DMSO-d_6.

Compound 16

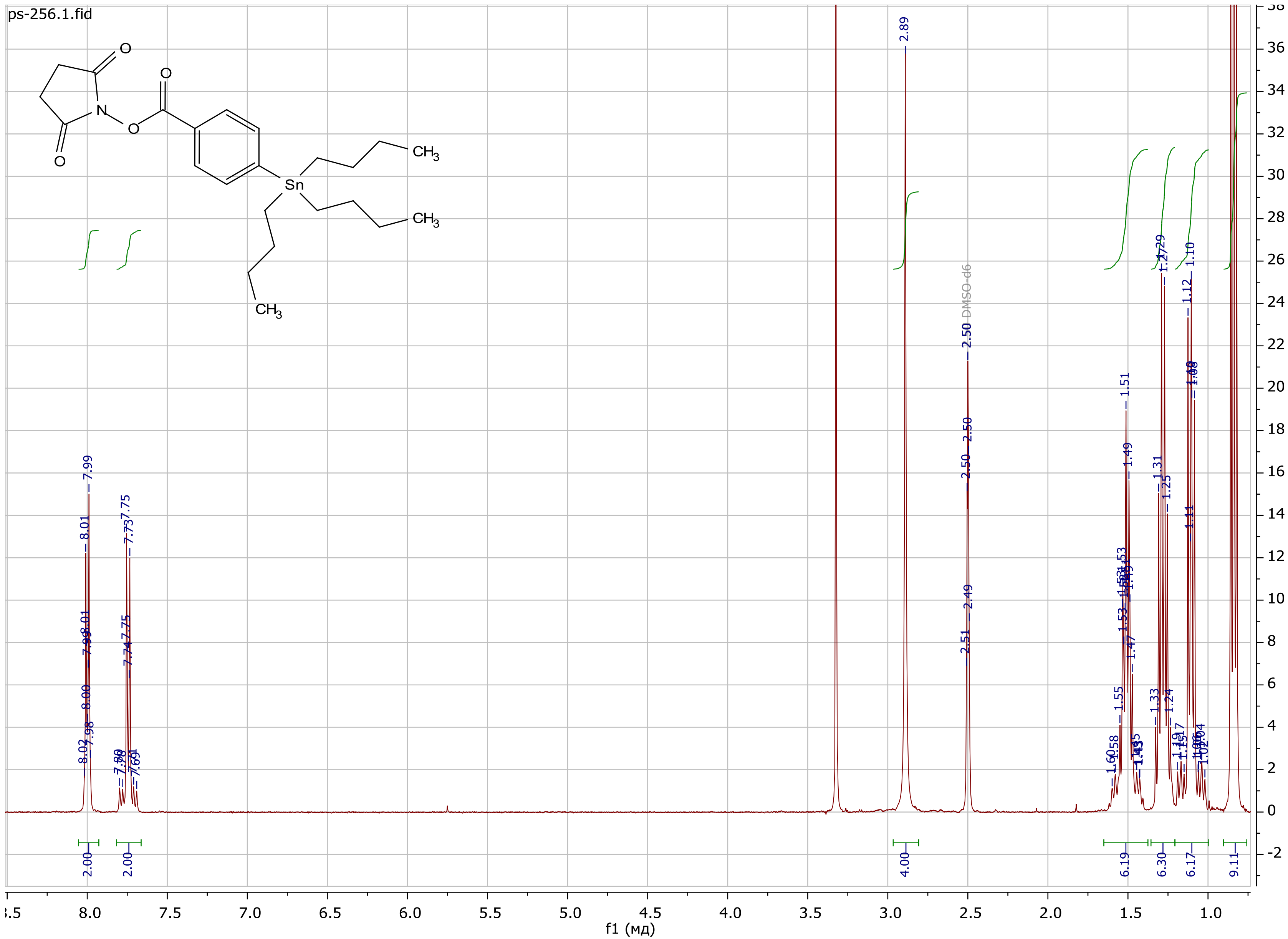

Figure S27. ^{1}H NMR spectrum of compound № **16** in DMSO-d_6.

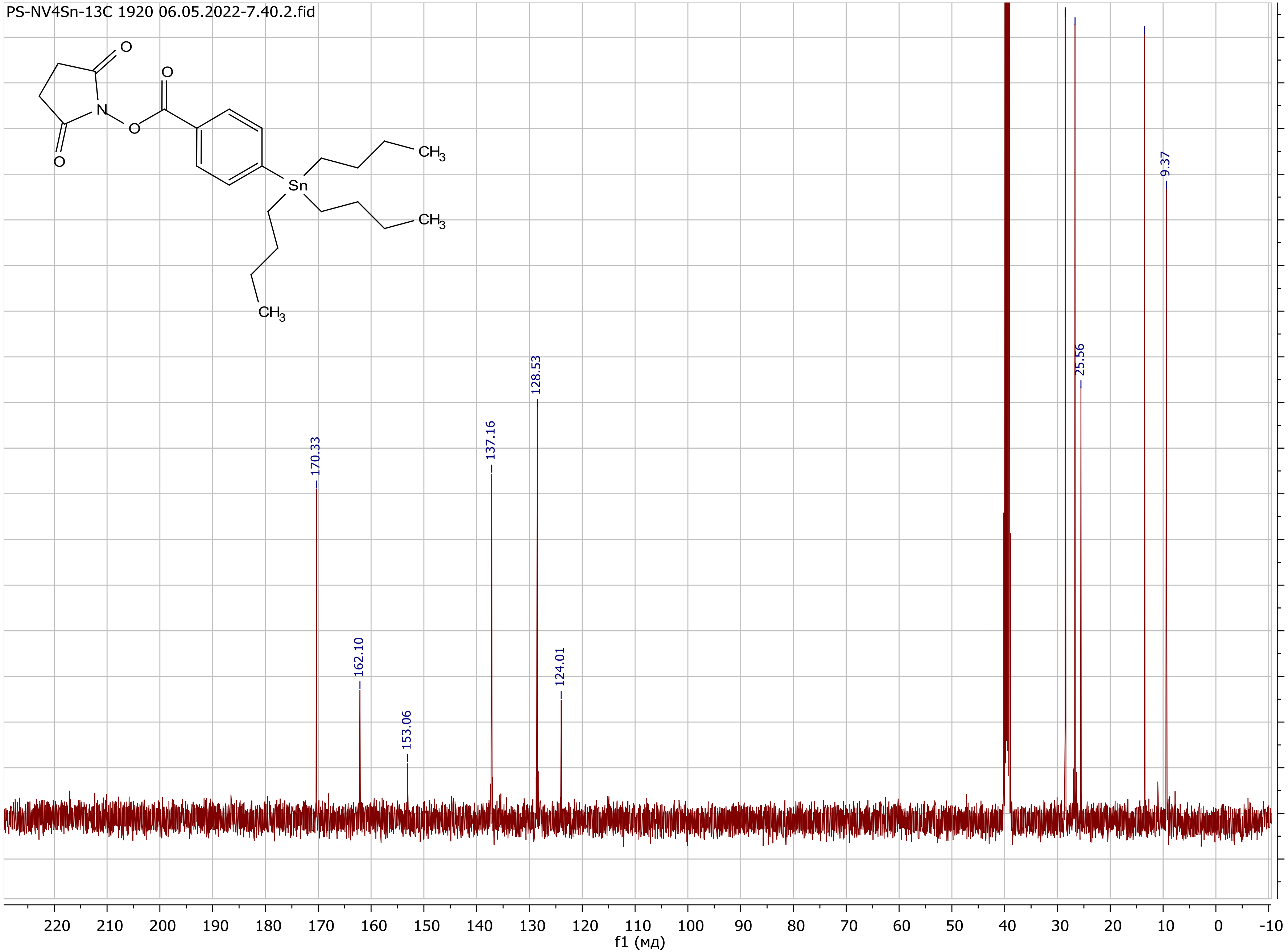

Figure S28. ^{13}C NMR spectrum of compound № **16** in DMSO-*d*$_6$.

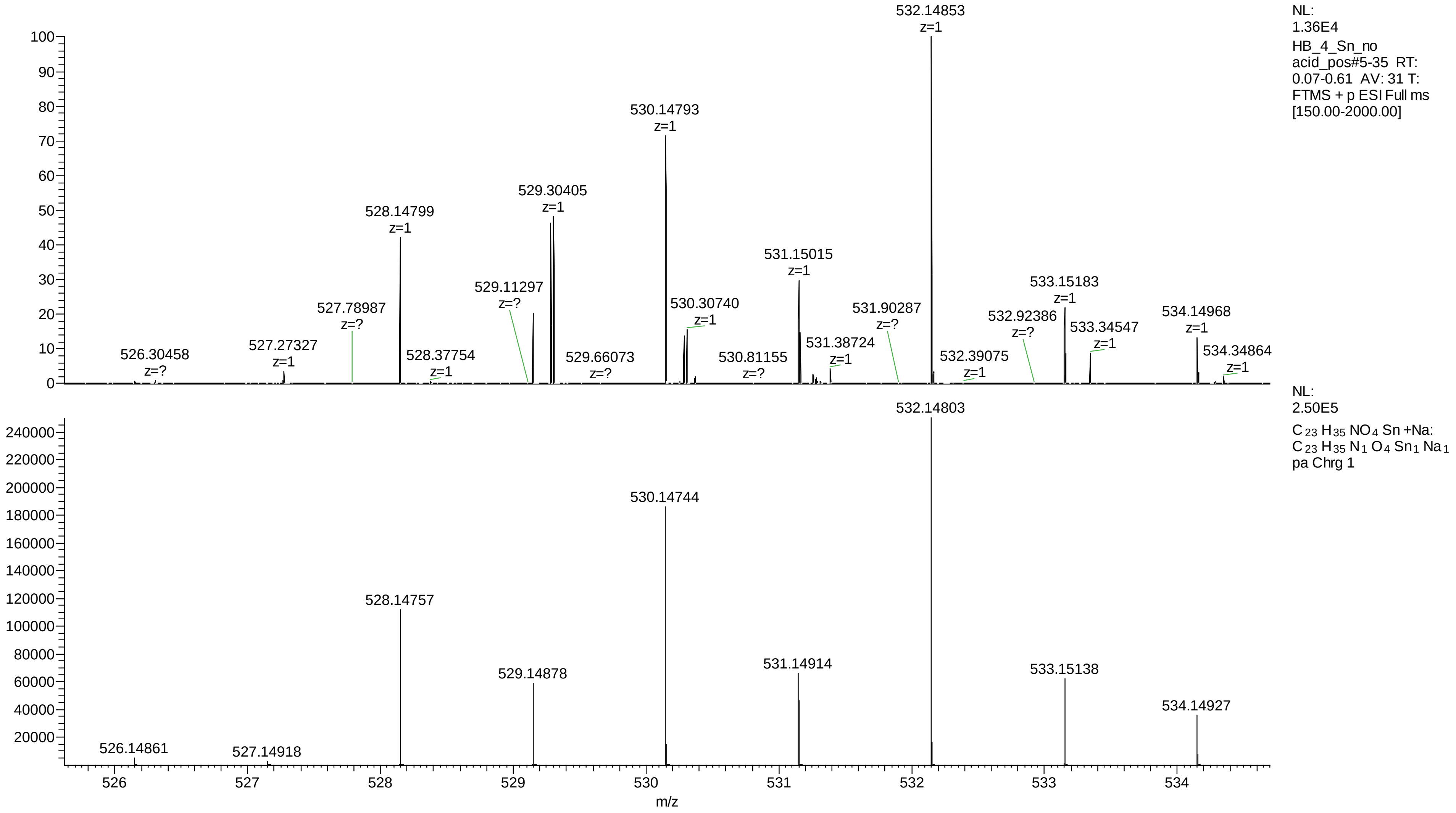

Figure S29. HRMS (m/z, ESI) of compound № **16**

Compound 17

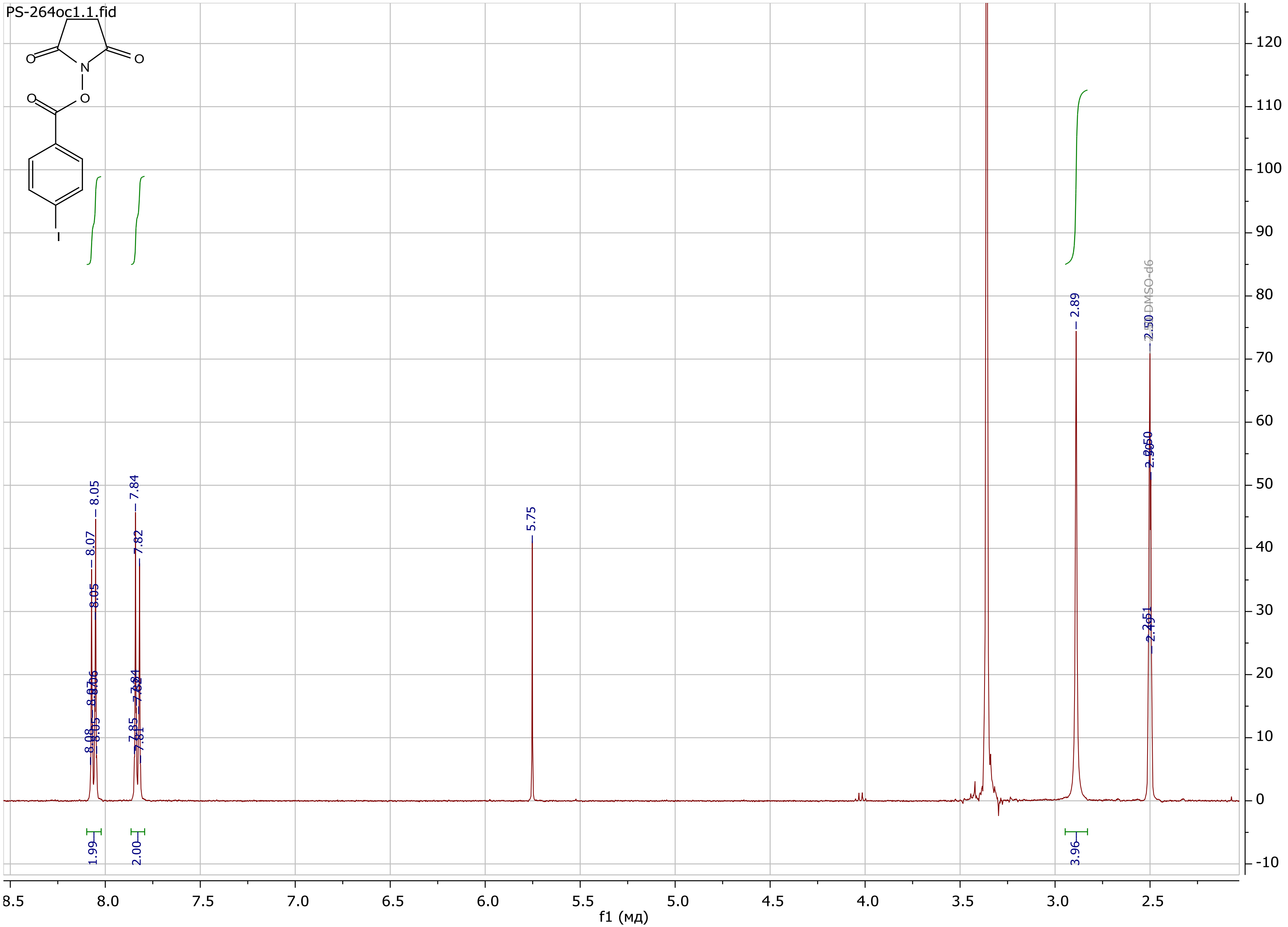

Figure S30. ^{1}H NMR spectrum of compound № **17** in DMSO-d_6.

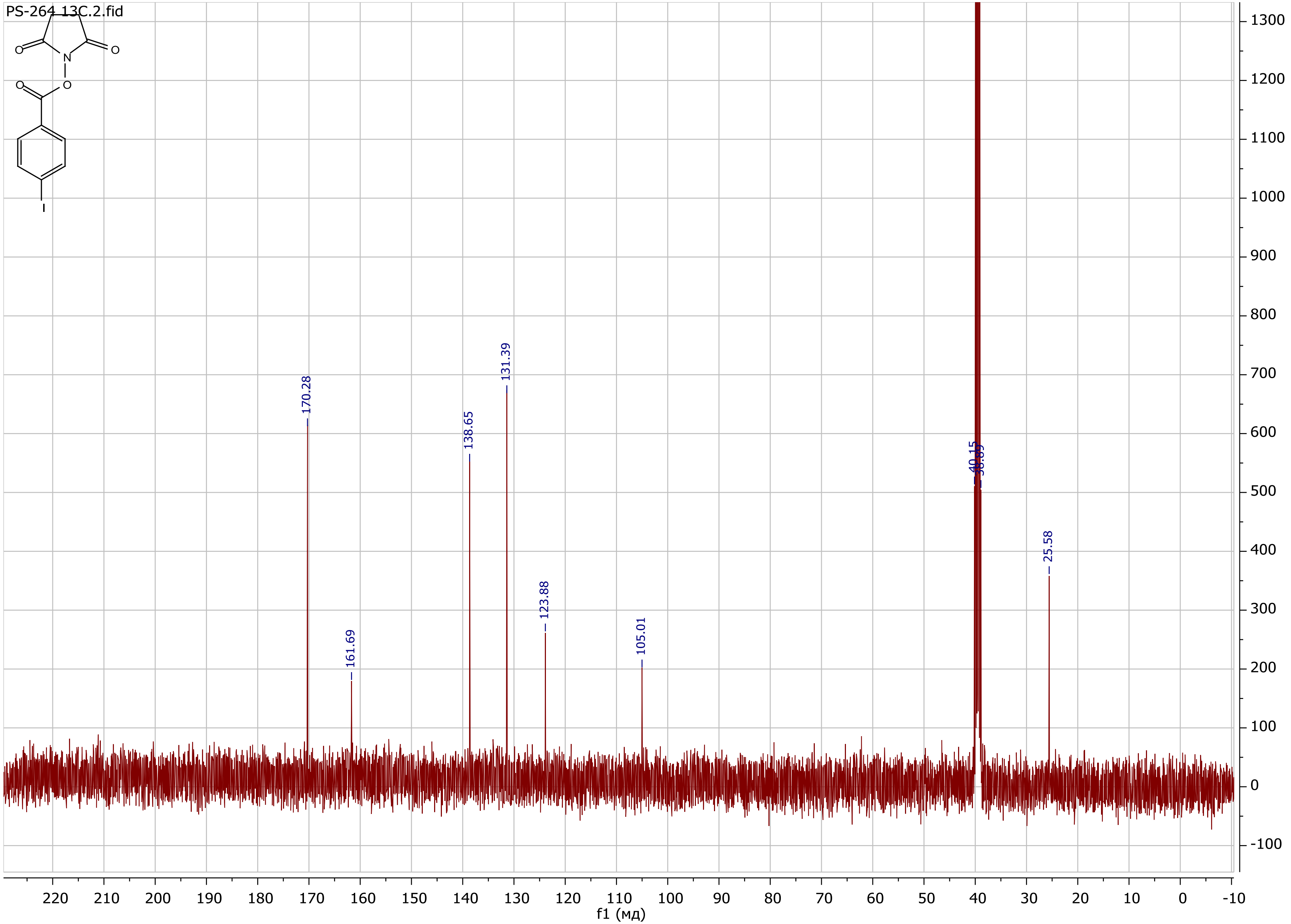

Figure S31. ^{13}C NMR spectrum of compound № **17** in DMSO-d_6.

Compound 18

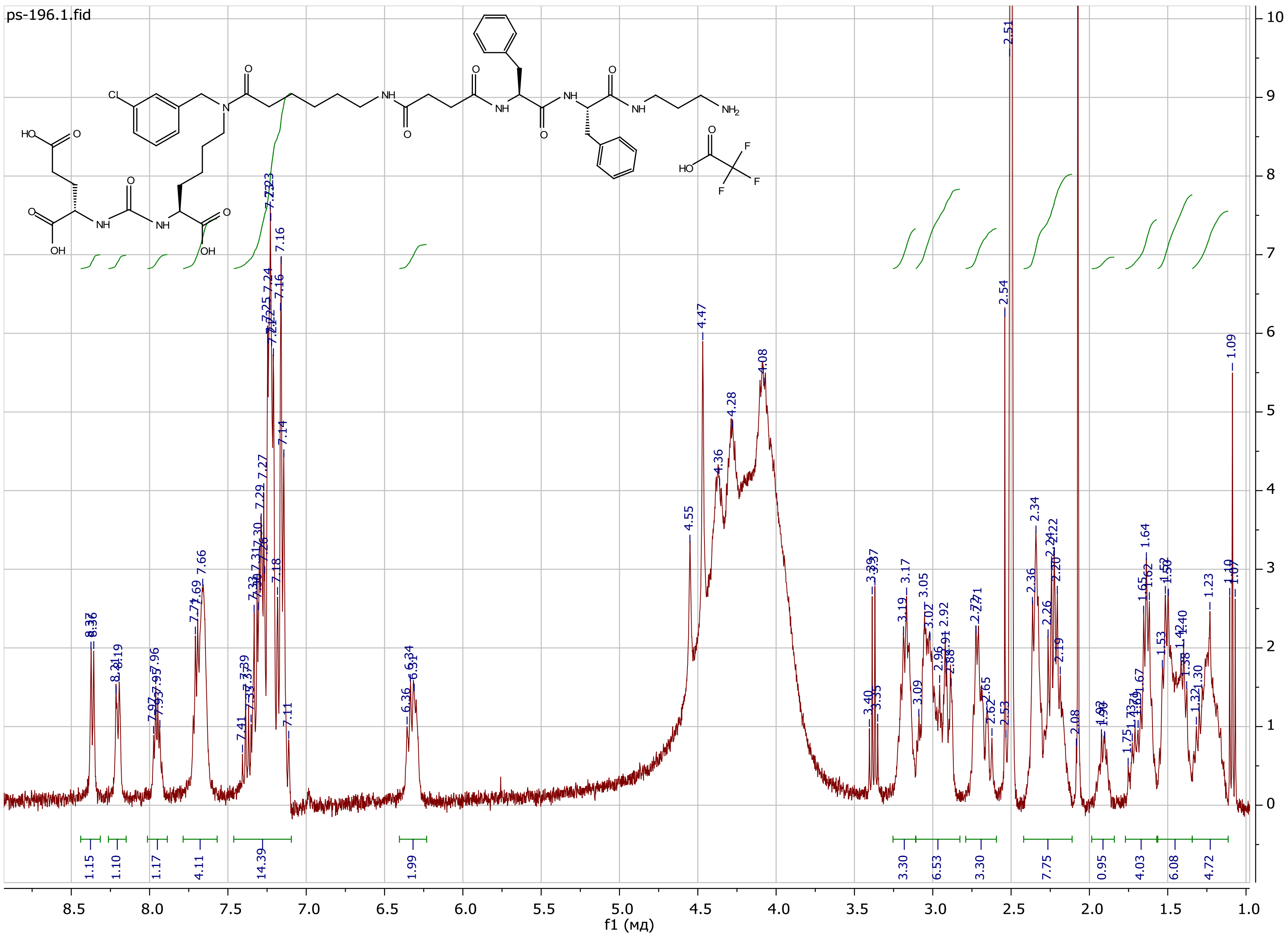

Figure S32. ^{1}H NMR spectrum of compound № **18** in DMSO-*d₆*.

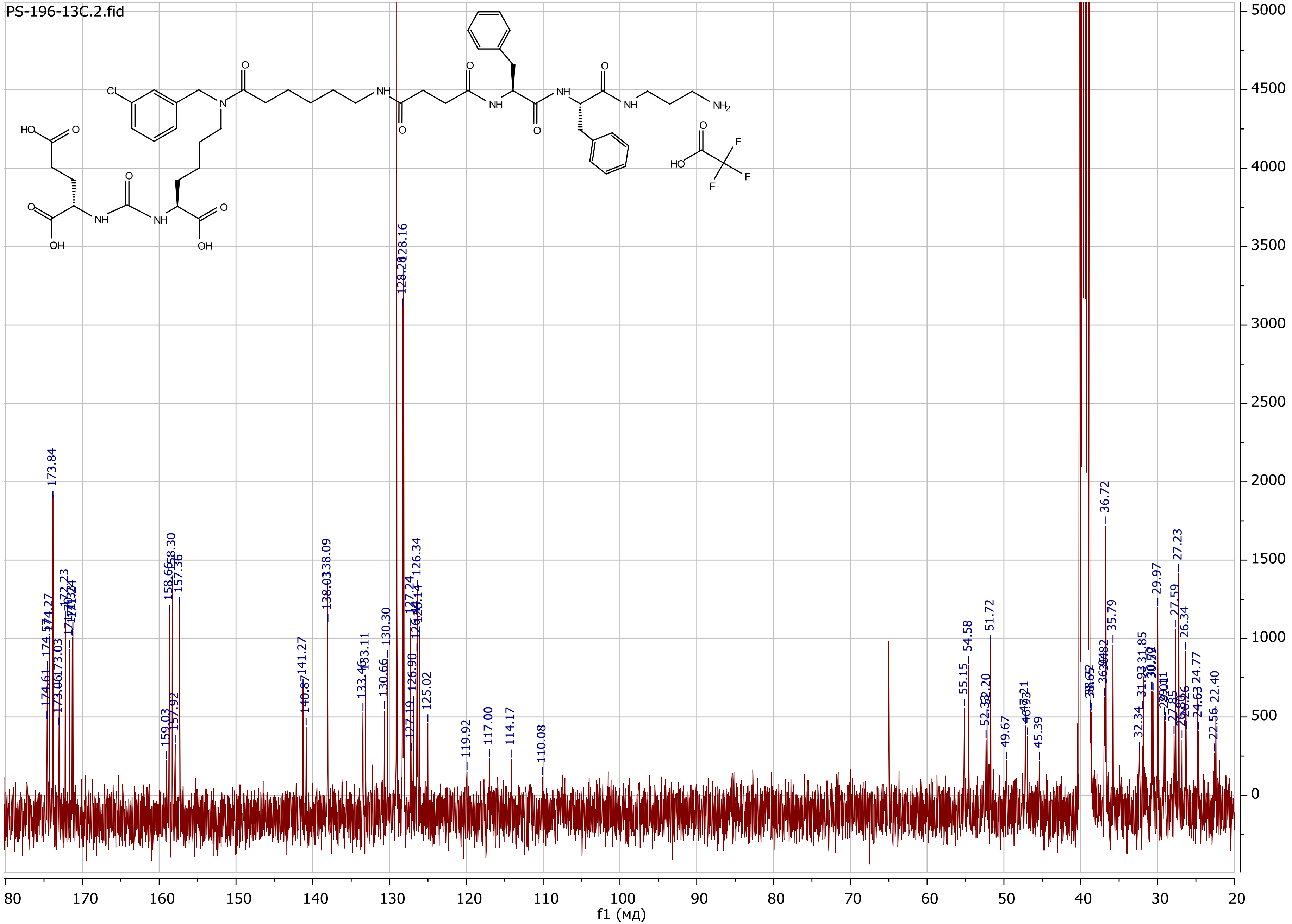

Figure S33. ^{13}C NMR spectrum of compound № **18** in DMSO-d_6.

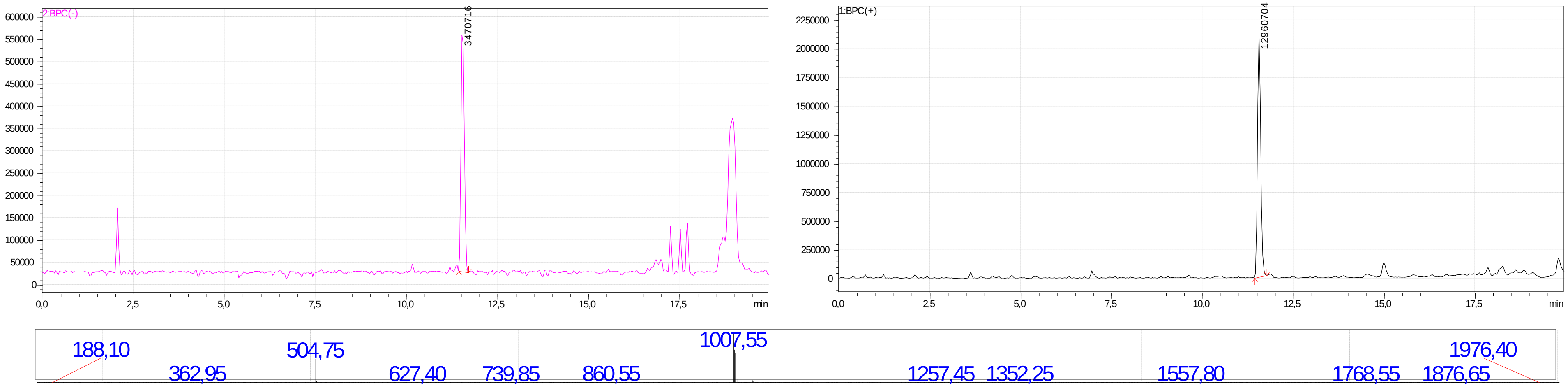

Figure S34. ESI-MS of compound № **18**

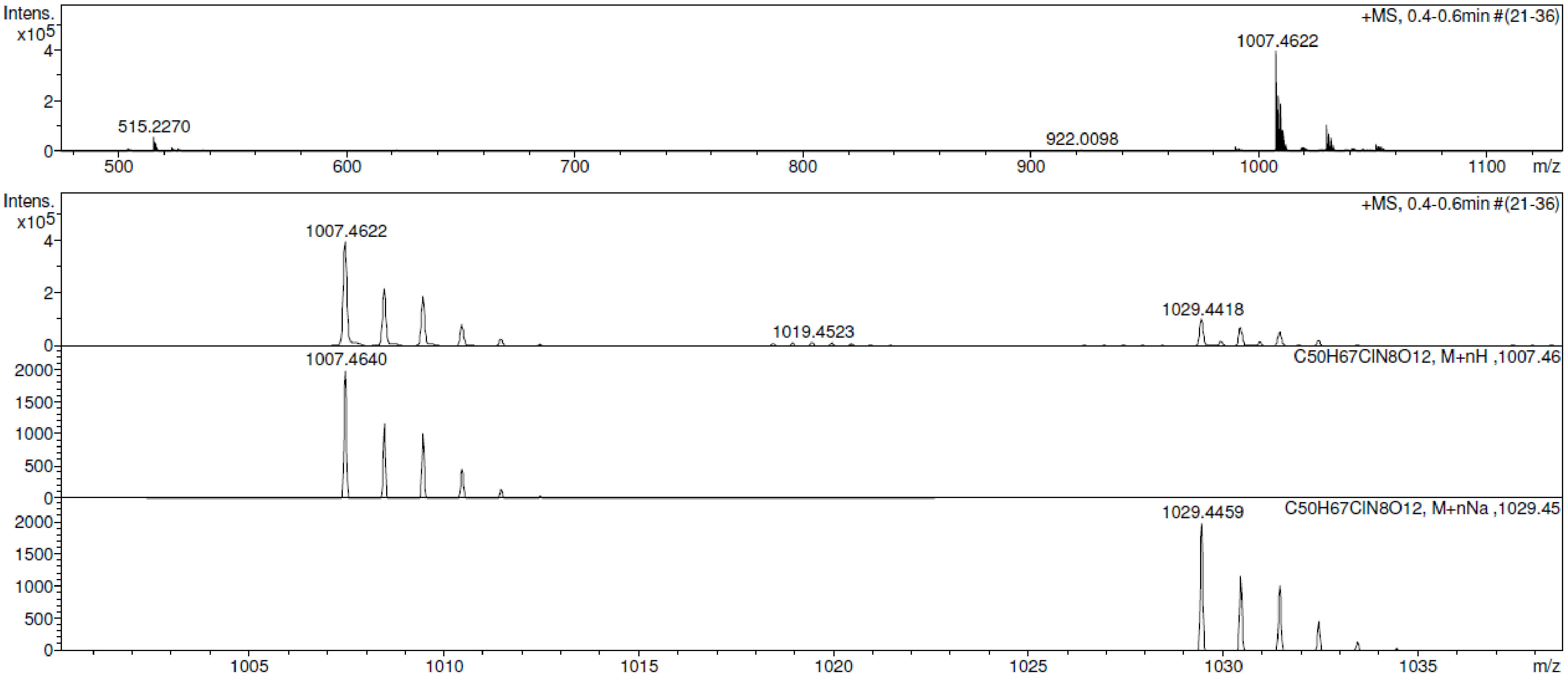

Figure S35. HRMS (m/z, ESI) of compound № **18**

Compound 19

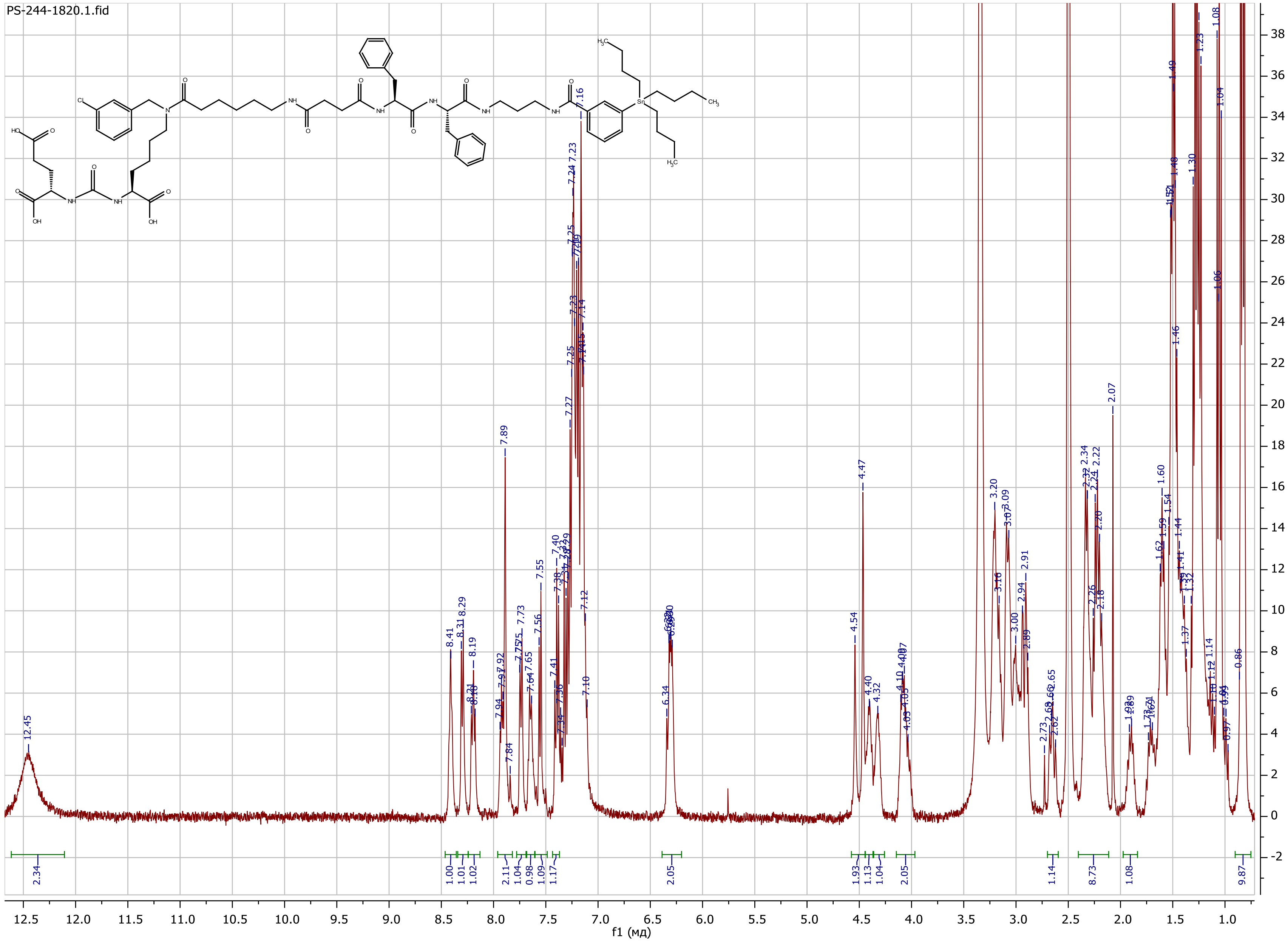

Figure S36. ¹H NMR spectrum of compound № **19** in DMSO-*d*₆.

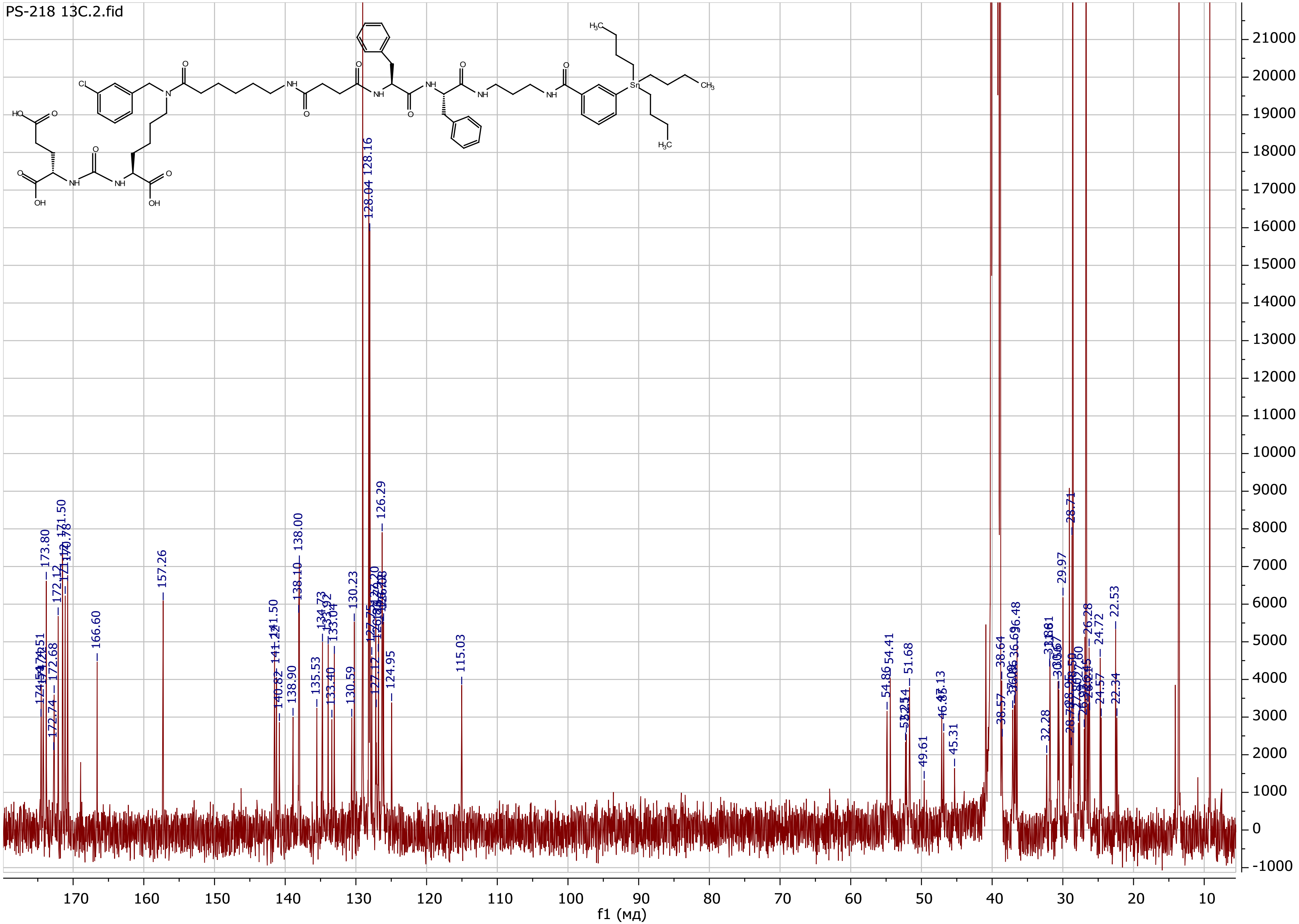

Figure S37. ^{13}C NMR spectrum of compound № **19** in DMSO-d_6.

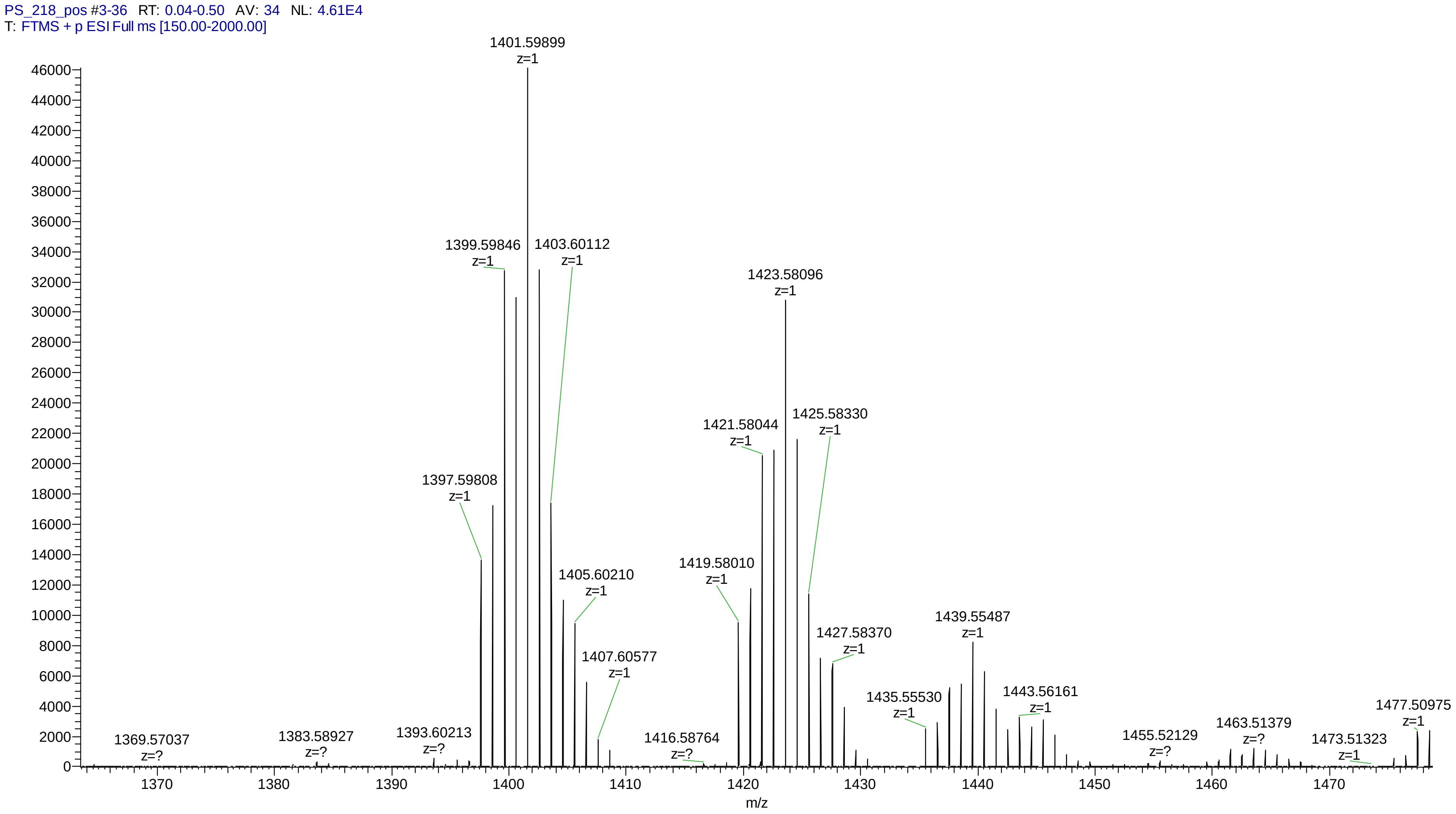

Figure S38. HRMS (m/z, ESI) of compound № **19**

Compound 20

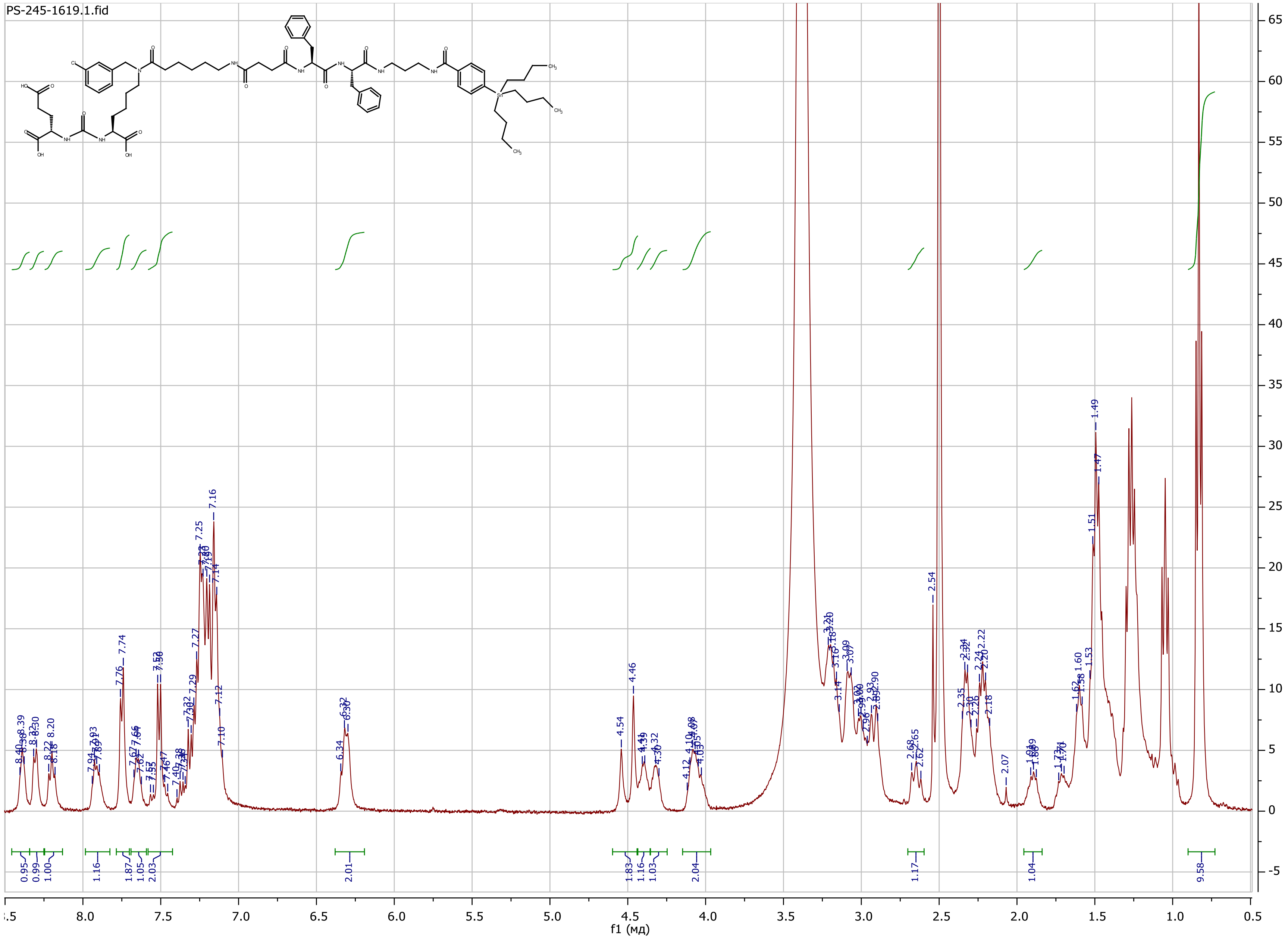

Figure S39. ^{1}H NMR spectrum of compound № **20** in DMSO-d_6.

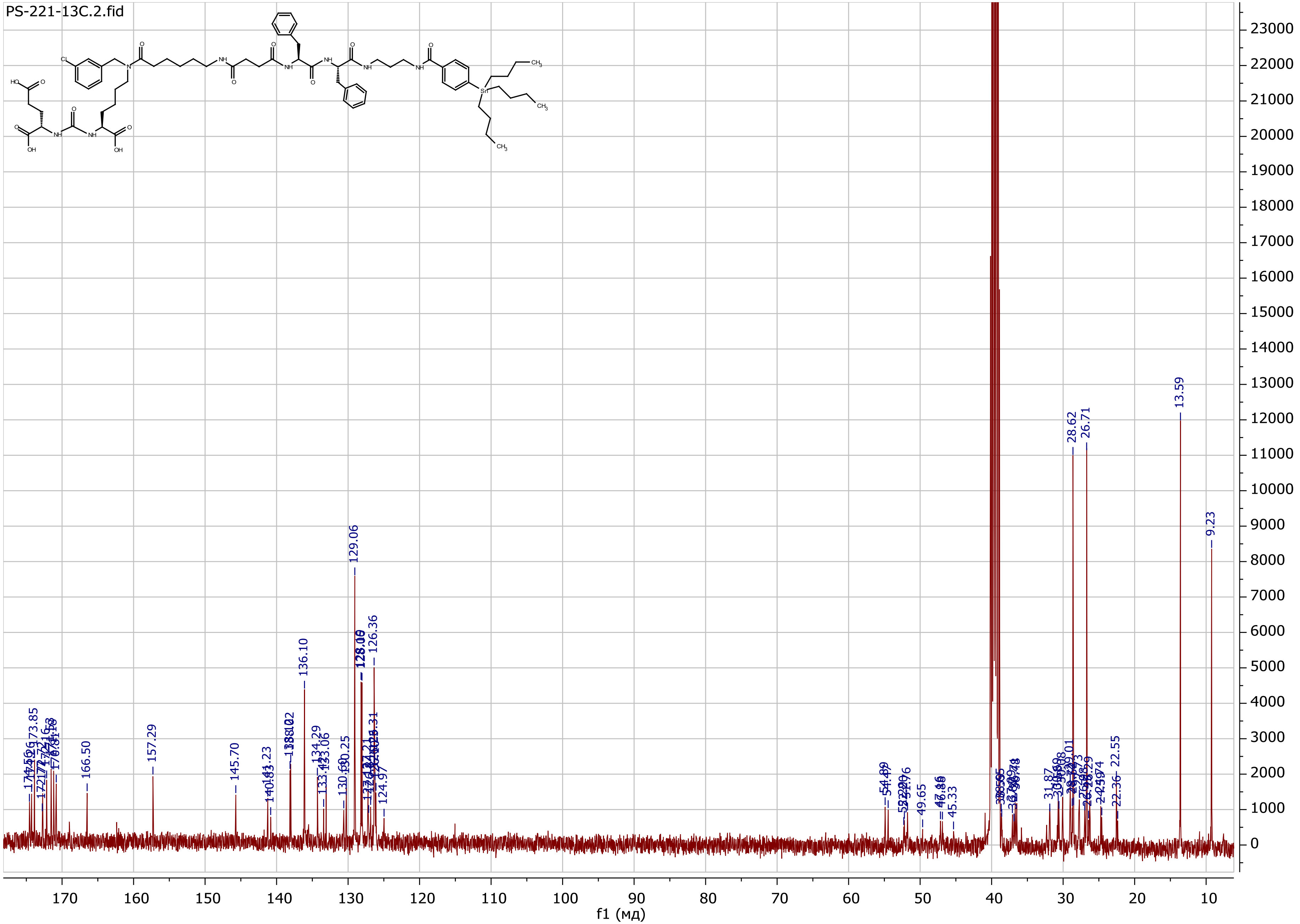

Figure S40. ^{13}C NMR spectrum of compound № **20** in DMSO-*d*$_6$.

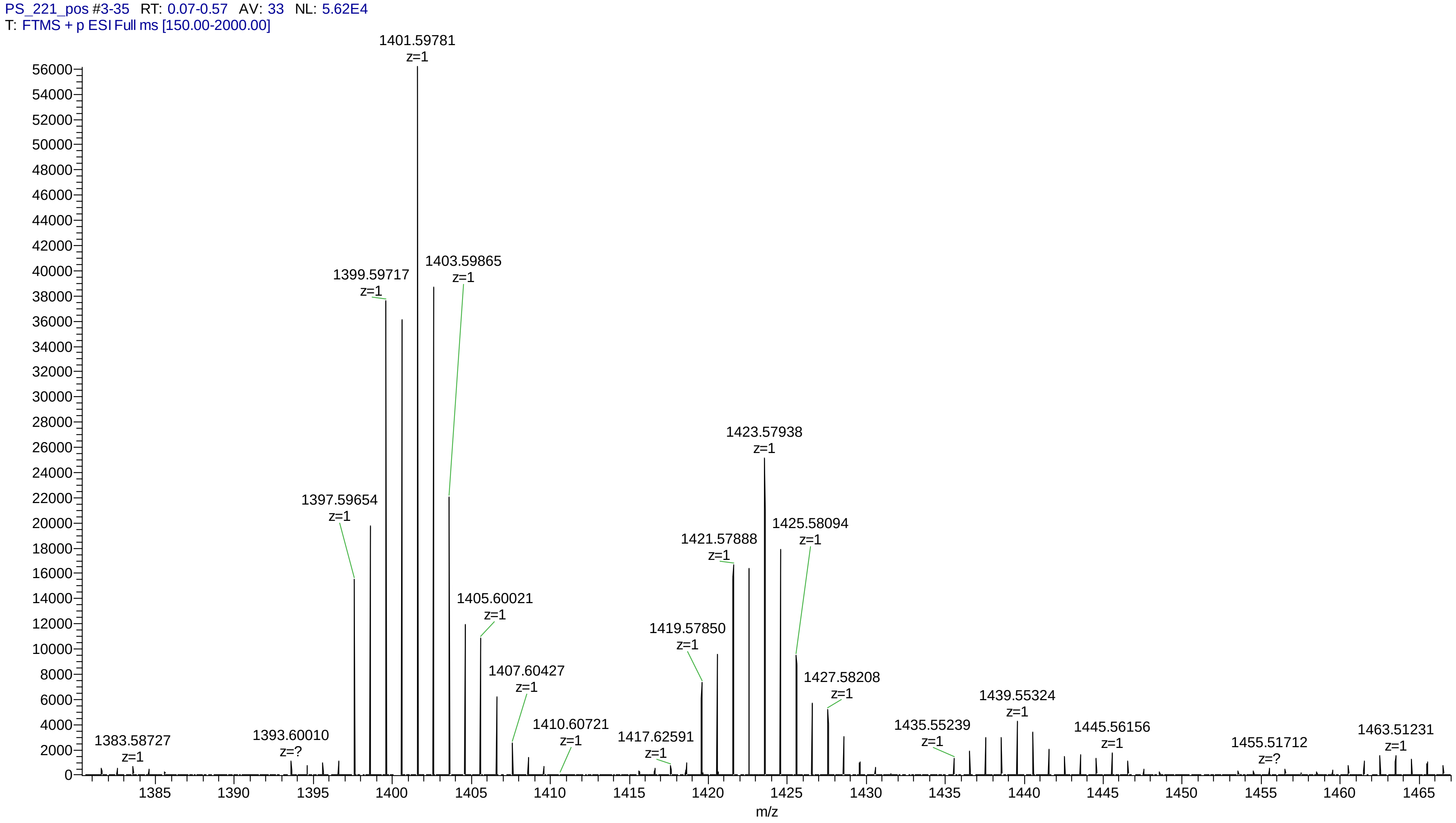

Figure S41. HRMS (m/z, ESI) of compound № **20**

Compound 21

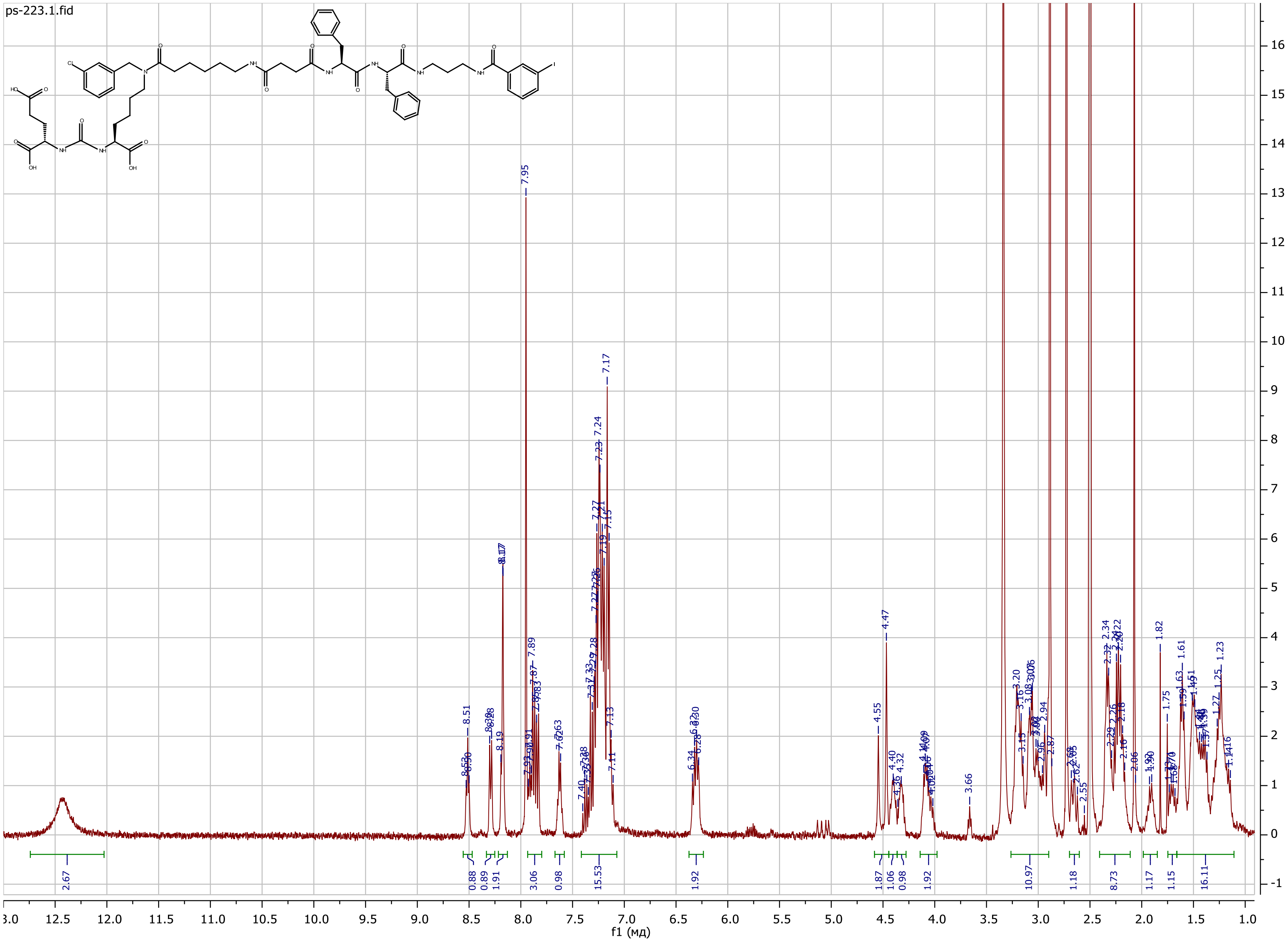

Figure S42. ^{1}H NMR spectrum of compound № **21** in DMSO-d_6.

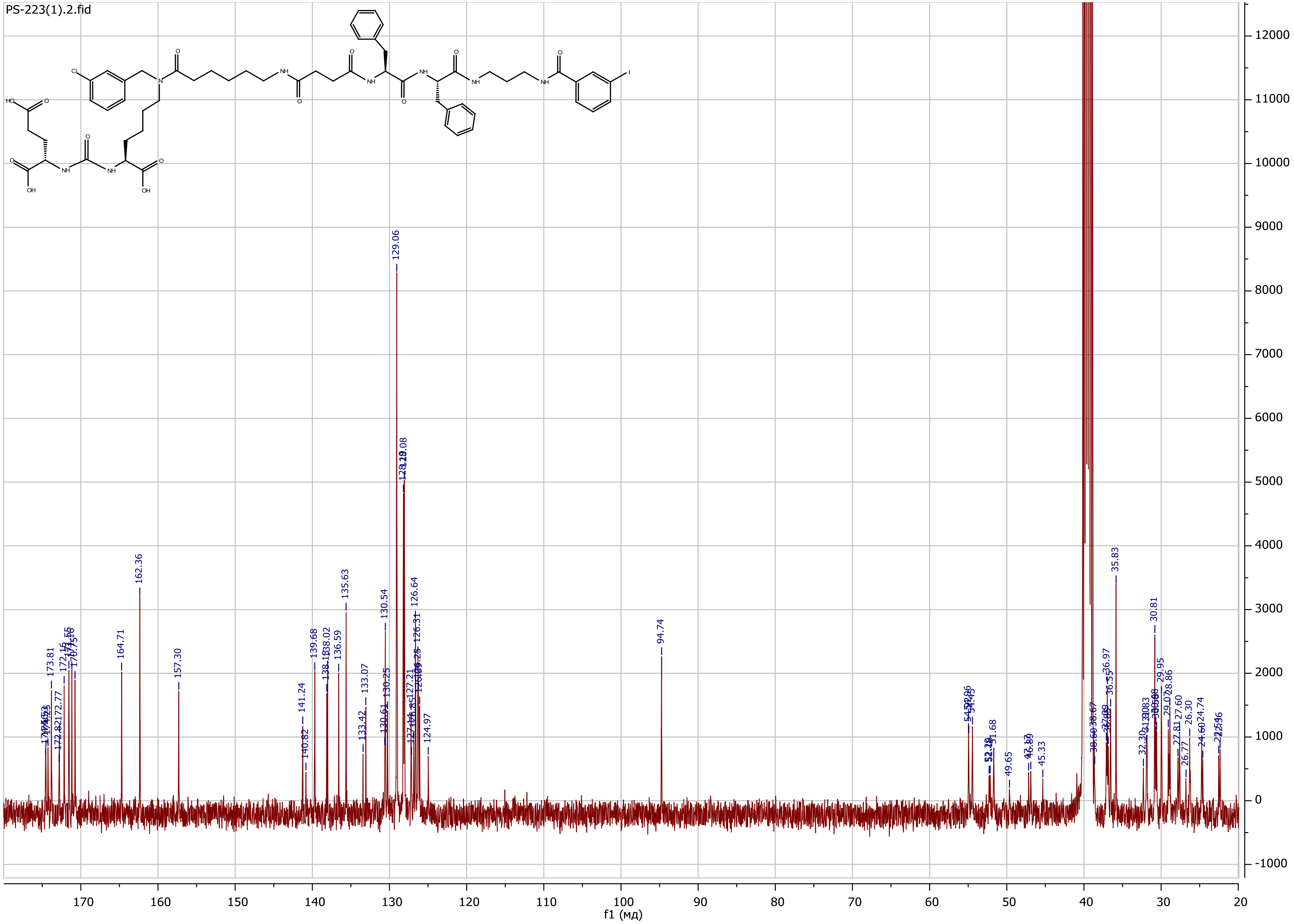

Figure S43. ¹³C NMR spectrum of compound № **21** in DMSO-d_6.

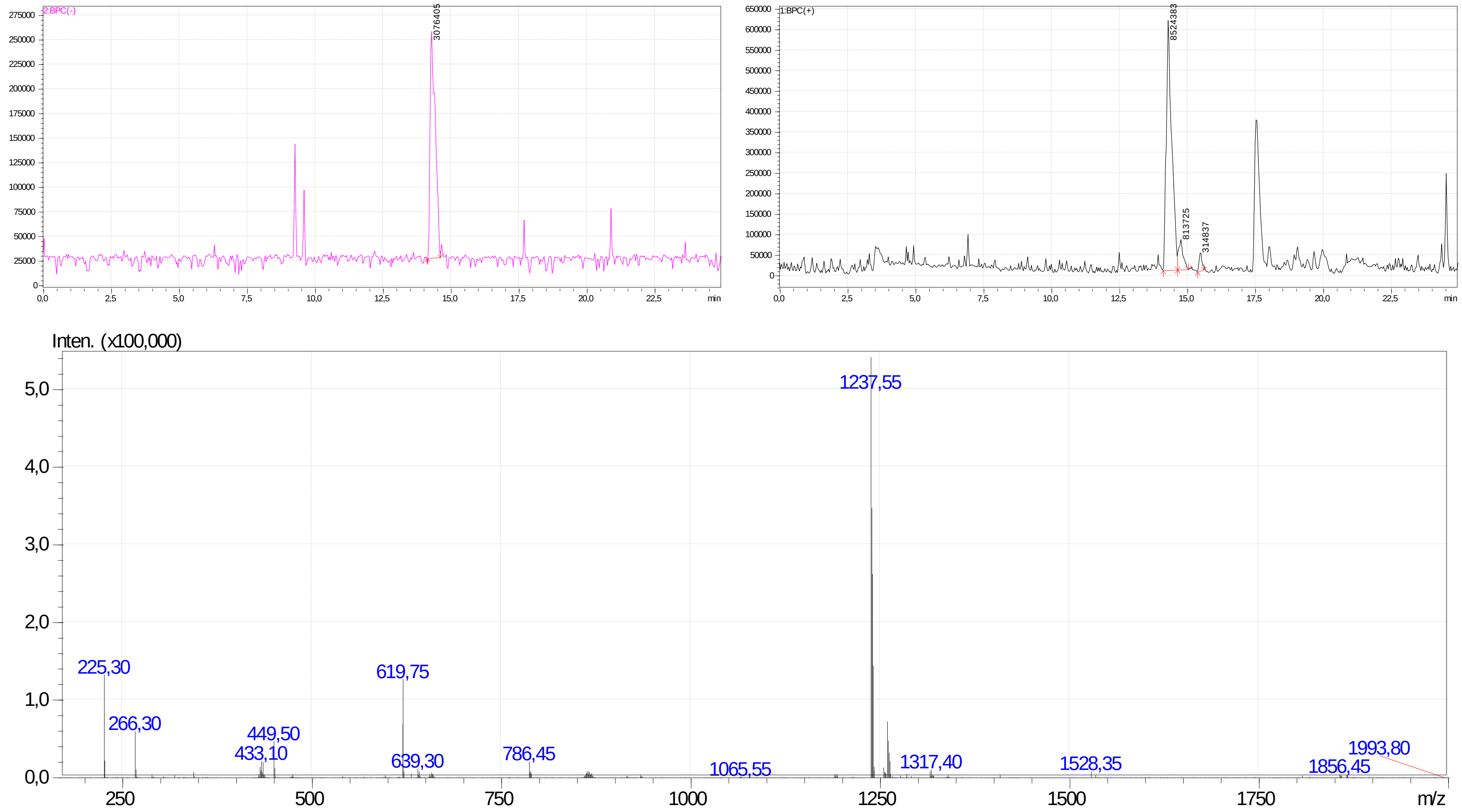

Figure S44. ESI-MS of compound № **21**

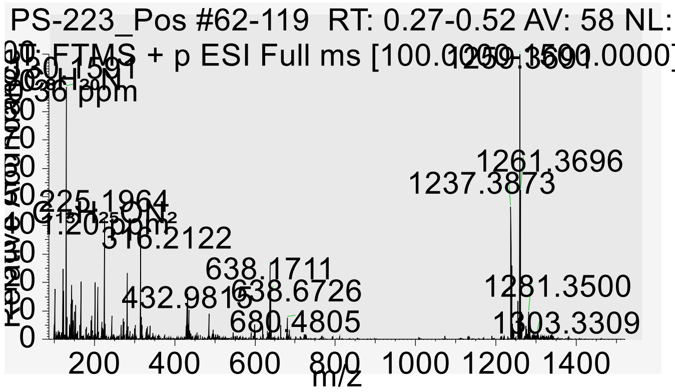

Figure S45. HRMS (m/z, ESI) of compound № **21**

Compound 22

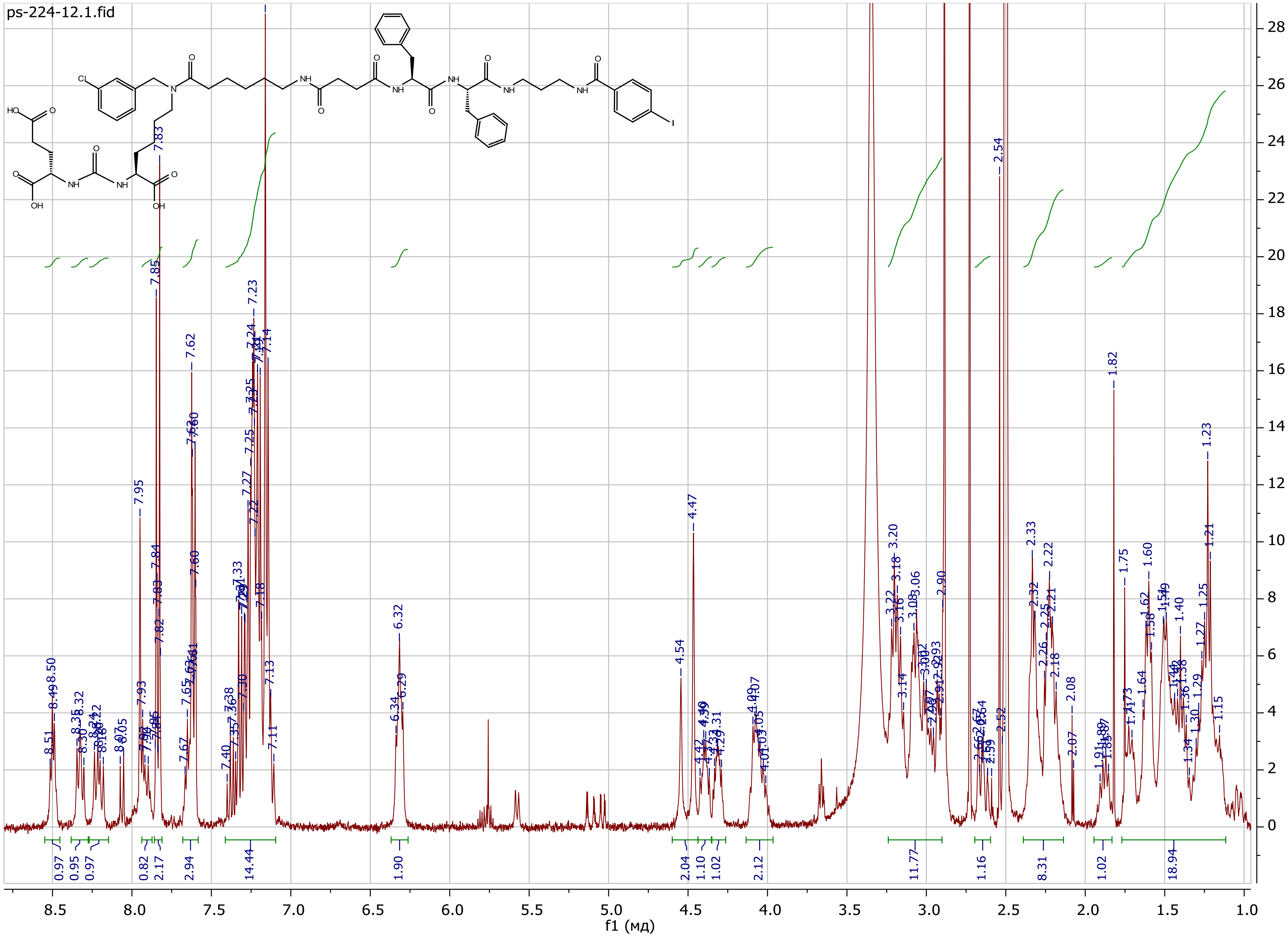

Figure S46. ^{1}H NMR spectrum of compound № **22** in DMSO-d_6.

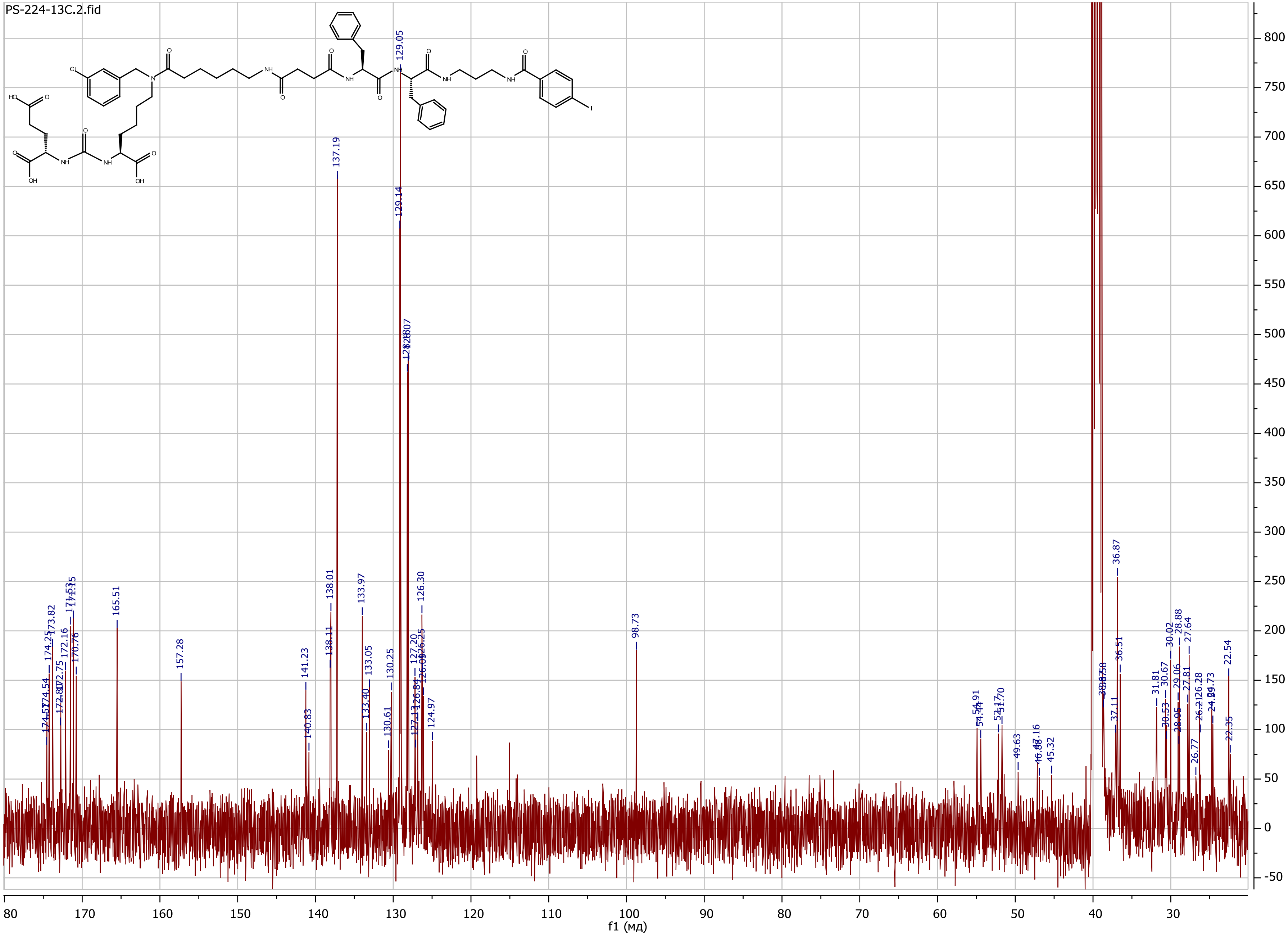

Figure S47. ^{13}C NMR spectrum of compound № **22** in DMSO-d_6.

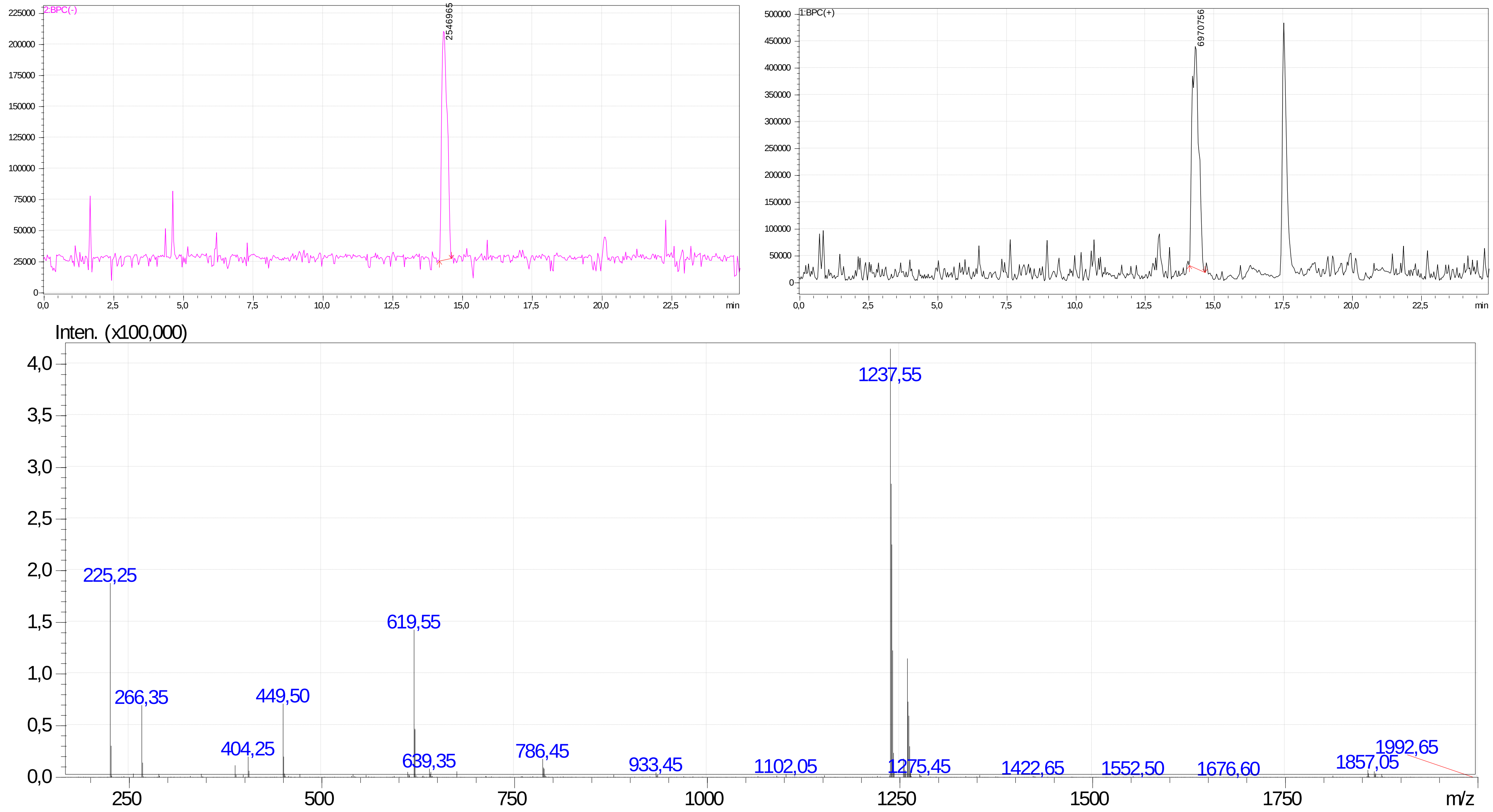

Figure S48. ESI-MS of compound № **22**

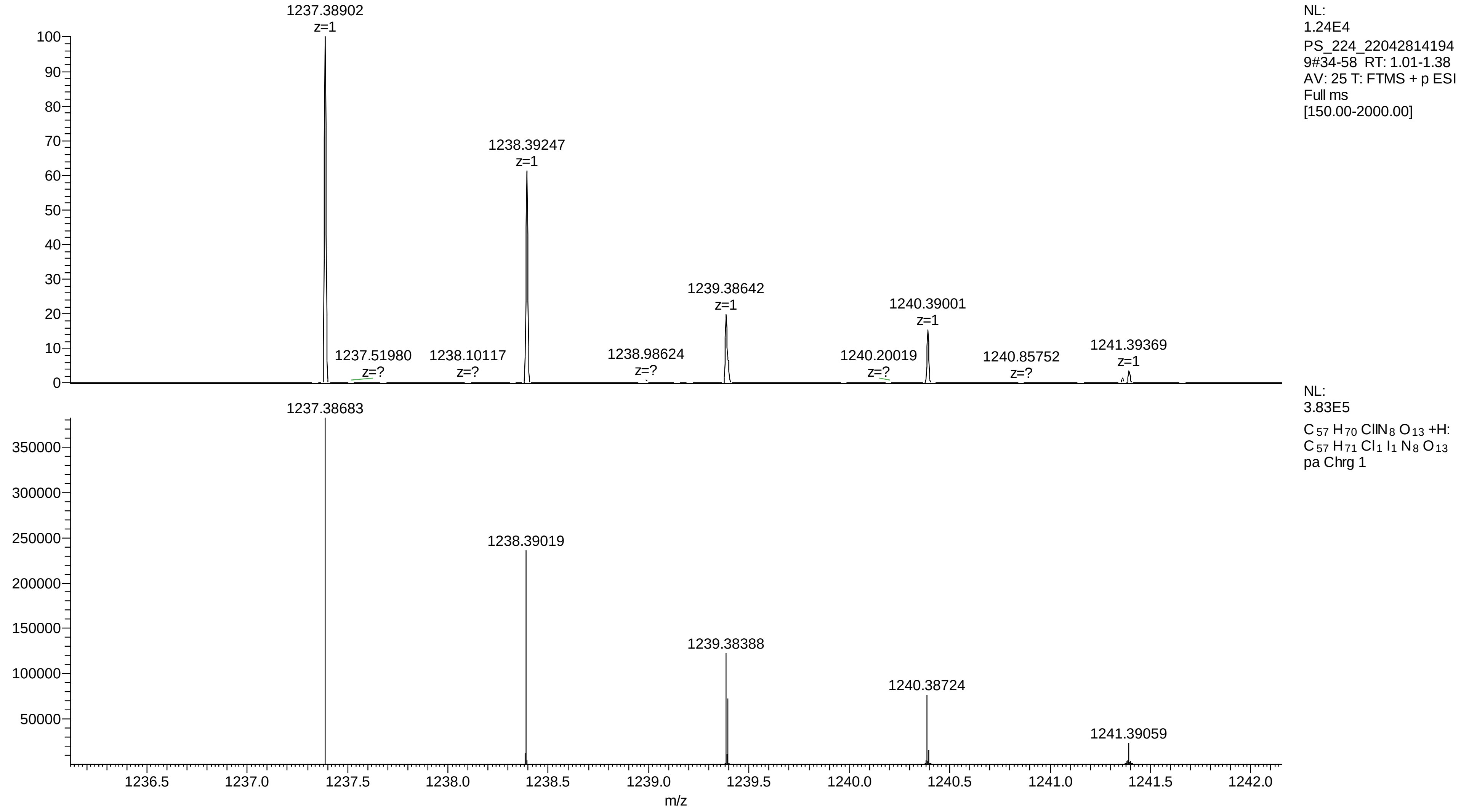

Figure S49. HRMS (m/z, ESI) of compound № **22**
